# Aberrant Expression Profiles of lncRNAs and Their Associated Nearby Coding Genes in the Hippocampus of the SAMP8 Mouse Model with AD

**DOI:** 10.1016/j.omtn.2020.02.008

**Published:** 2020-02-19

**Authors:** Honghai Hong, Yousheng Mo, Dongli Li, Zhiheng Xu, Yanfang Liao, Ping Yin, Xinning Liu, Yong Xia, Jiansong Fang, Qi Wang, Shuhuan Fang

**Affiliations:** 1 Department of Clinical Laboratory, The Third Affiliated Hospital of Guangzhou Medical University, 63 Duobao Road, Guangzhou, Guangdong Province, China; 2 Department of Pathology and Laboratory Medicine, Indiana University School of Medicine, Indianapolis, IN 46202, USA; 3 Science and Technology Innovation Center, Guangzhou University of Chinese Medicine, Guangzhou, Guangdong Province, China; 4 Department of Gastrointestinal Surgery, The First Affiliated Hospital of Guangzhou University of Chinese Medicine, Guangzhou, Guangdong Province, China; 5 DME Center, Institute of Clinical Pharmacology, Guangzhou University of Chinese Medicine, Guangzhou, Guangdong Province, China; 6 Department of Cardiothoracic Surgery, Stanford University School of Medicine, Stanford, CA, USA

**Keywords:** lncRNAs, lncRNA-associated nearby genes, Alzheimer’s disease

## Abstract

The senescence-accelerated mouse prone 8 (SAMP8) mouse model is a useful model for investigating the fundamental mechanisms involved in the age-related learning and memory deficits of Alzheimer’s disease (AD), while the SAM/resistant 1 (SAMR1) mouse model shows normal features. Recent evidence has shown that long non-coding RNAs (lncRNAs) may play an important role in AD pathogenesis. However, a comprehensive and systematic understanding of the function of AD-related lncRNAs and their associated nearby coding genes in AD is still lacking. In this study, we collected the hippocampus, the main area of AD pathological processes, of SAMP8 and SAMR1 animals and performed microarray analysis to identify aberrantly expressed lncRNAs and their associated nearby coding genes, which may contribute to AD pathogenesis. We identified 3,112 differentially expressed lncRNAs and 3,191 differentially expressed mRNAs in SAMP8 mice compared to SAMR1 mice. More than 70% of the deregulated lncRNAs were intergenic and exon sense-overlapping lncRNAs. Gene Ontology (GO) and pathway analyses of the AD-related transcripts were also performed and are described in detail, which imply that metabolic process reprograming was likely related to AD. Furthermore, six lncRNAs and six mRNAs were selected for further validation of the microarray results using quantitative PCR, and the results were consistent with the findings from the microarray. Moreover, we analyzed 780 lincRNAs (also called long “intergenic” non-coding RNAs) and their associated nearby coding genes. Among these lincRNAs, AK158400 had the most genes nearby (n = 13), all of which belonged to the histone cluster 1 family, suggesting regulation of the nucleosome structure of the chromosomal fiber by affecting nearby genes during AD progression. In addition, we also identified 97 aberrant antisense lncRNAs and their associated coding genes. It is likely that these dysregulated lncRNAs and their associated nearby coding genes play a role in the development and/or progression of AD.

## Introduction

Alzheimer’s disease (AD) is considered an age-related neurodegenerative disease with a progressive impairment in cognitive function that is characterized by the presence of senile plaques and neurofibrillary tangles.^1^^,^^2^ The hippocampus is one of the most important brain regions for learning and memory and is the main impaired region of AD.^3^ The progressive memory deterioration of AD results in the loss of autonomy, and patients ultimately require full-time medical care.^4^ Unfortunately, there are no effective therapeutic strategies to prevent the progression of AD currently.^5^ Therefore, revealing the molecular mechanism of AD is necessary for developing effective therapy. The debate over whether senile plaques and neurofibrillary tangles are causative or merely markers of the disease has been ongoing for most of the past century. Hence, despite considerable research, including studies of various genes and proteins in this area, the detailed mechanism of AD is still limited, and we should focus on molecules other than genes and proteins that may play an important role in AD.

Long non-coding RNAs (lncRNAs), a novel kind of non-coding RNA that ranges from 200 nt to more than 100 kb and usually lacks an obvious open reading frame, have received increasing attention for their involvement in the pathogenesis of many diseases.^6^, ^7^, ^8^ Emerging data strongly suggest that lncRNAs are important for the basal regulation of protein coding genes at the transcriptional and posttranscriptional levels.^9^ Abnormalities among lncRNAs, in regard to their sequence, spatial structure, expression, and interaction with proteins, have been found to play important roles in AD pathogenesis.^10^^,^^11^ Research has shown that β-site amyloid precursor protein (APP)-cleaving enzyme-1 (BACE1) antisense transcript (BACE1-AS) regulates BACE1 expression at both the mRNA and protein levels, which could enhance APP processing and Aβ_1–42_ production as well as plaque deposition.^12^^,^^13^ 51A is a novel lncRNA that is overexpressed in *in vitro* models and the brain of individuals with AD, which was shown to regulate the expression of alternatively spliced SORL1 variants and subsequently increase amyloid formation.^14^^,^^15^ 17A, NDM29, and NAT-Rad18 were also reported to be involved in the mechanism of AD.^16^, ^17^, ^18^ However, until now, only a few studies have examined the roles of lncRNAs in AD, and our understanding of AD-associated lncRNAs has been limited to preliminary explorations. Thus, the identification of the genome-wide expression and the functional significance of AD-associated lncRNAs and their associated nearby coding genes is necessary.

In the present study, we utilized microarray technology to analyze the expression profiles of lncRNAs and mRNAs in the hippocampus of 8-month-old senescence-accelerated mouse (SAM) prone 8 (SAMP8) mice with AD and age-matched SAM/resistant 1 (SAMR1) mice. The aim of this study was to systematically explore the lncRNA and mRNA expression profiles, the related pathways, and the associated nearby coding genes of the lncRNAs, all of which may contribute to the understanding of AD pathogenesis and provide a valuable resource for the diagnosis and therapy of AD in the clinic.

## Results

### Learning and Memory Abilities of SAMP8 Mice

**Figure 1:**
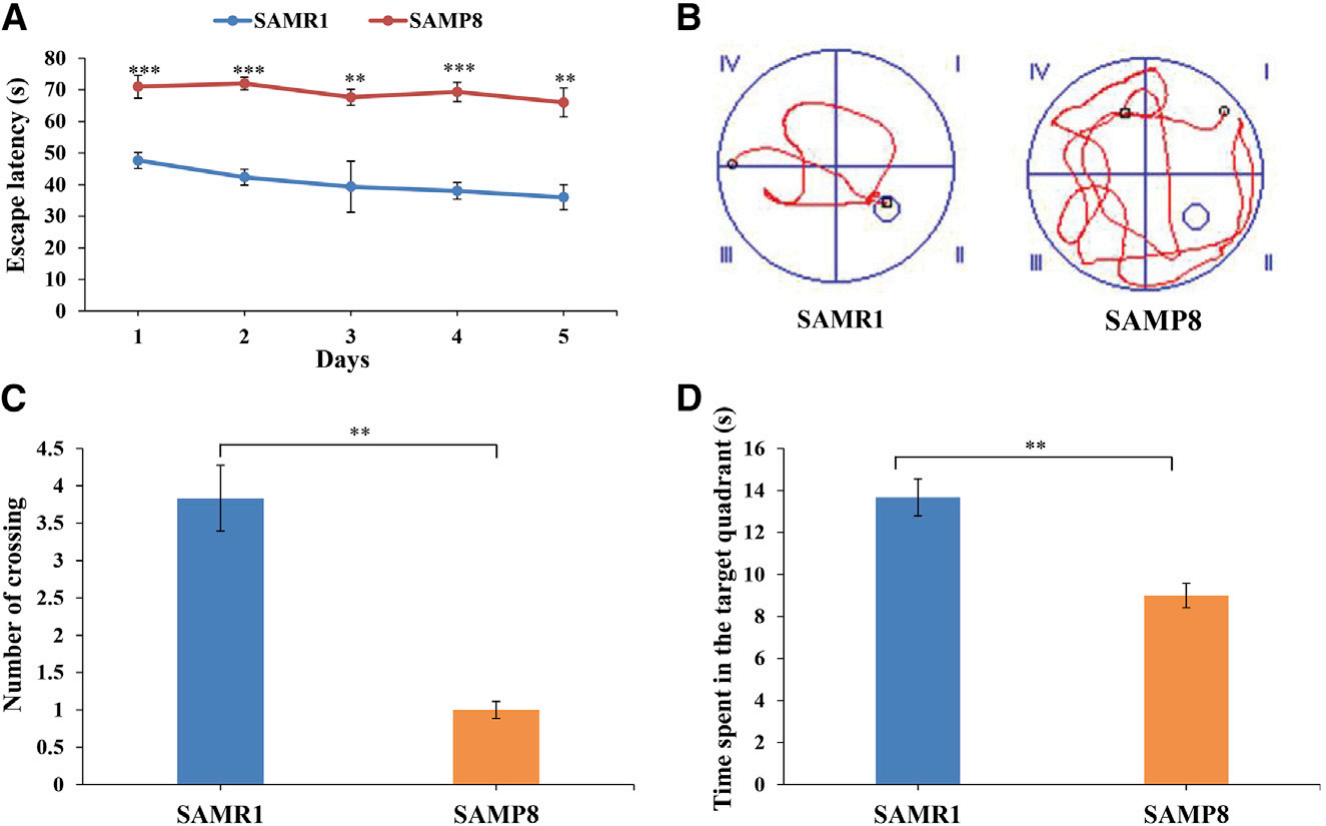
Learning and Memory Abilities of SAMP8 Mice (A) Escape latency of SAMP8 (n = 10) mice and SAMR1 (n = 10) littermates in the Morris water maze test. ∗∗p < 0.01, ∗∗∗p < 0.001. (B) Drawings are representations of single-mouse distances to the platform location. (C) Number of crossings in the probe trial test. ∗∗p < 0.01. (D) Time spent in the target quadrant in the probe trial test. ∗∗p < 0.01.

To evaluate the learning and memory abilities of 8-month-old SAMP8 mice, we performed the Morris water maze test. Compared to age-matched SAMR1 mice, 8-month-old SAMP8 mice exhibited obviously increased escape latencies and traveled a greater distance before finding the hidden platform (Figures 1A and 1B), implying that the AD model mice had worse learning performances. Additionally, in the probe test, the number of platform crossings and time spent in the target quadrant of the SAMP8 mice were significantly reduced compared to SAMR1 mice (Figures 1C and 1D). Taken together, these results indicated that 8-month-old SAMP8 mice exhibited severe learning and cognitive impairments and spontaneously developed AD, which was also consistent with our previous studies.^19^

### Overview of the lncRNA Expression Profiles in Hippocampal Tissues of 8-Month-Old SAMP8 Mice Compared to Age-Matched SAMR1 Mice

**Figure 2:**
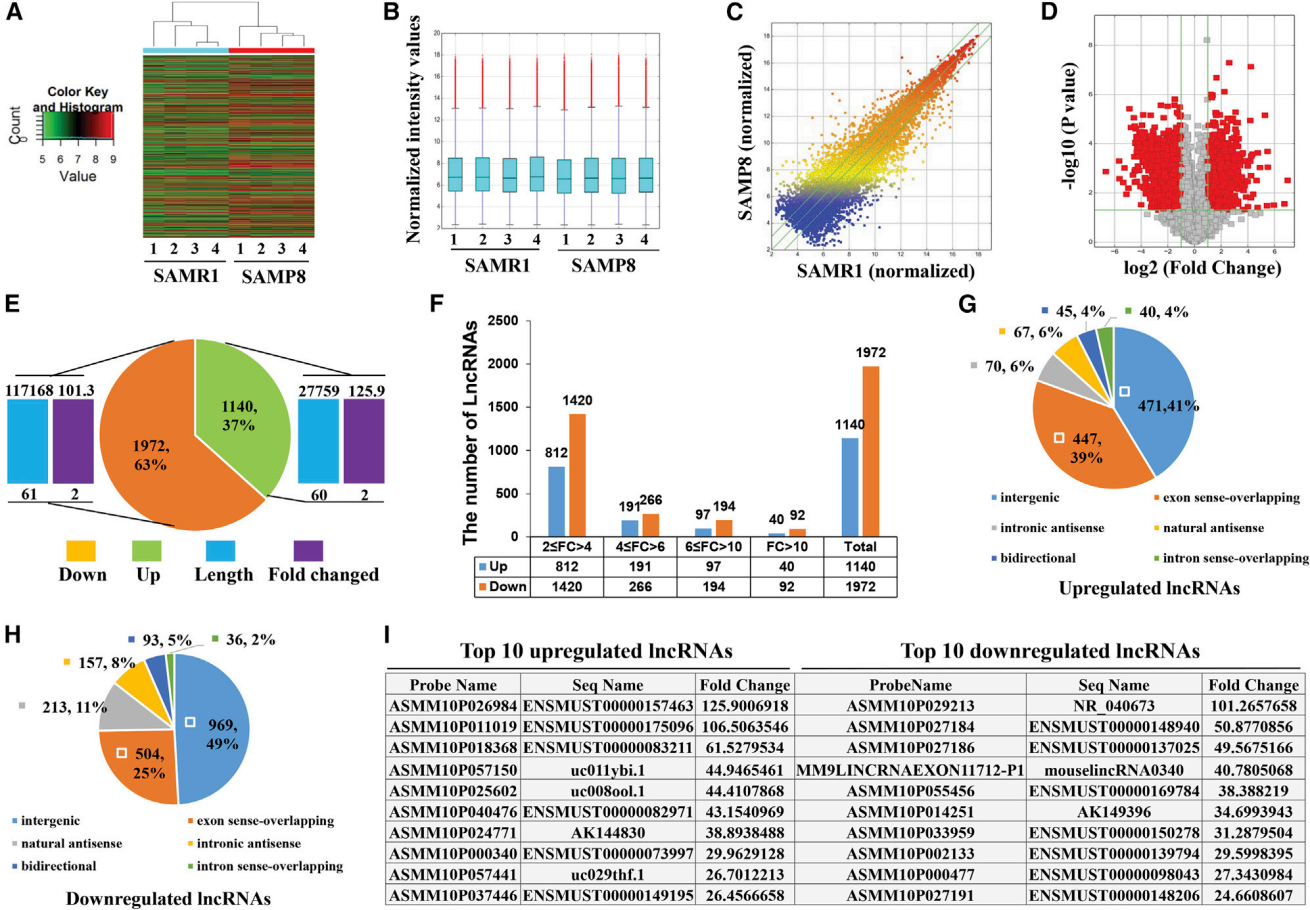
Overview of lncRNA Expression Profiles in Hippocampal Tissues of 8-Month-Old SAMP8 Mice Compared with Age-Matched SAMR1 Mice (A) Hierarchical clustering of differentially expressed lncRNAs. Green indicates low intensity, black indicates medium intensity, and red indicates strong intensity. (B) Boxplot of differentially expressed lncRNAs in each group. (C) Scatterplot of lncRNA signal values visualizing the variation (or reproducibility) between the two groups. The green lines represent the fold change lines. The lncRNAs above the top green line and below the bottom green line demonstrated more than a 2-fold change of lncRNA expression between the two compared samples. (D) Volcano plot of the differential expression of lncRNAs. The vertical lines correspond to 2-fold upregulation and downregulation, respectively. The horizontal line represents a p value of 0.05, and the red points on the plot represent the differentially expressed lncRNAs with statistical significance. (E) Pie chart shows the number of upregulated and downregulated lncRNAs with the length and fold change. Green indicates upregulated lncRNA, orange indicates downregulated lncRNA, blue bar indicates length of lncRNA, and brown bar indicates fold change of lncRNA. (F) Number of lncRNAs in the different subgroups classified by fold change (FC). Blue and orange bars indicate the number of upregulated and downregulated lncRNAs, respectively. (G and H) Pie chart shows the classification of the lncRNAs. According the genomic positions, upregulated (G) and downregulated (H) lncRNAs were classified as bidirectional, exon sense-overlapping, intergenic, intron sense-overlapping, intronic antisense, and natural antisense. (I) Top 10 significantly upregulated and downregulated lncRNAs in the microarray data.

We used an Arraystar mouse lncRNA microarray v3.0 to analyze the expression profiles of lncRNAs in four pairs of hippocampal tissues from SAMP8 and SAMR1 mice. The lncRNA expression patterns of the hippocampal tissues of the two groups of mice were classified by using hierarchical clustering and boxplots, as illustrated in Figures 2A and 2B. A scatterplot and volcano plot depicting the variation in lncRNA expression in the two groups are shown in Figures 2C and 2D. The results showed that a total of 21,314 aberrantly expressed lncRNAs were identified in the hippocampal tissues of SAMP8 mice compared with age-matched SAMR1, of which 9,639 lncRNAs had upregulated expression and 11,675 displayed downregulated expression (Tables S1 and S2) in the SAMP8 mice. When utilizing a fold change and p value cutoff >2.0 and <0.05, respectively, it was found that 1,140 lncRNAs (the length and fold change ranged from 60 to 27,759 and 2 to 125.9, respectively) had upregulated expression, and 1,972 lncRNAs (the length and fold change ranged from 61 to 117,168 and 2 to 101.3, respectively) displayed downregulated expression, among the 3,112 differentially expressed lncRNAs (Figure 2E; Table S3). The lncRNAs were carefully identified with the use of the most authoritative databases, such as RefSeq, UCSC_knowngene, Ensembl, UCR, lincRNA, and many related studies (Figure S1). The number of dysregulated lncRNAs varied among the subgroups with different fold changes, with most of the lncRNAs falling in the 2 ≤ fold change < 4 subgroup (Figure 2F). According to the various genomic positions of the lncRNAs with respect to other genes, all of the aberrantly expressed lncRNAs were classified as one of the following six types: bidirectional, exon sense-overlapping, intergenic, intron sense-overlapping, intronic antisense, and natural antisense. We found that more than 70% of the dysregulated lncRNAs belonged to the intergenic and exon sense-overlapping groups, regardless of whether the lncRNAs were upregulated (Figure 2G) or downregulated (Figure 2H). In addition, the most upregulated lncRNA, ENSMUST00000157463 (fold change, 125.9), and the most downregulated lncRNA, ASMM10P029213 (fold change, 101.3), as well as the top 10 distinctly regulated lncRNAs, are shown in Figure 2I. These lncRNAs may contribute to AD pathogenesis.

### Expression Profiles of mRNAs in Hippocampal Tissues of 8-Month-Old SAMP8 Mice Compared to Age-Matched SAMR1 Mice

**Figure 3:**
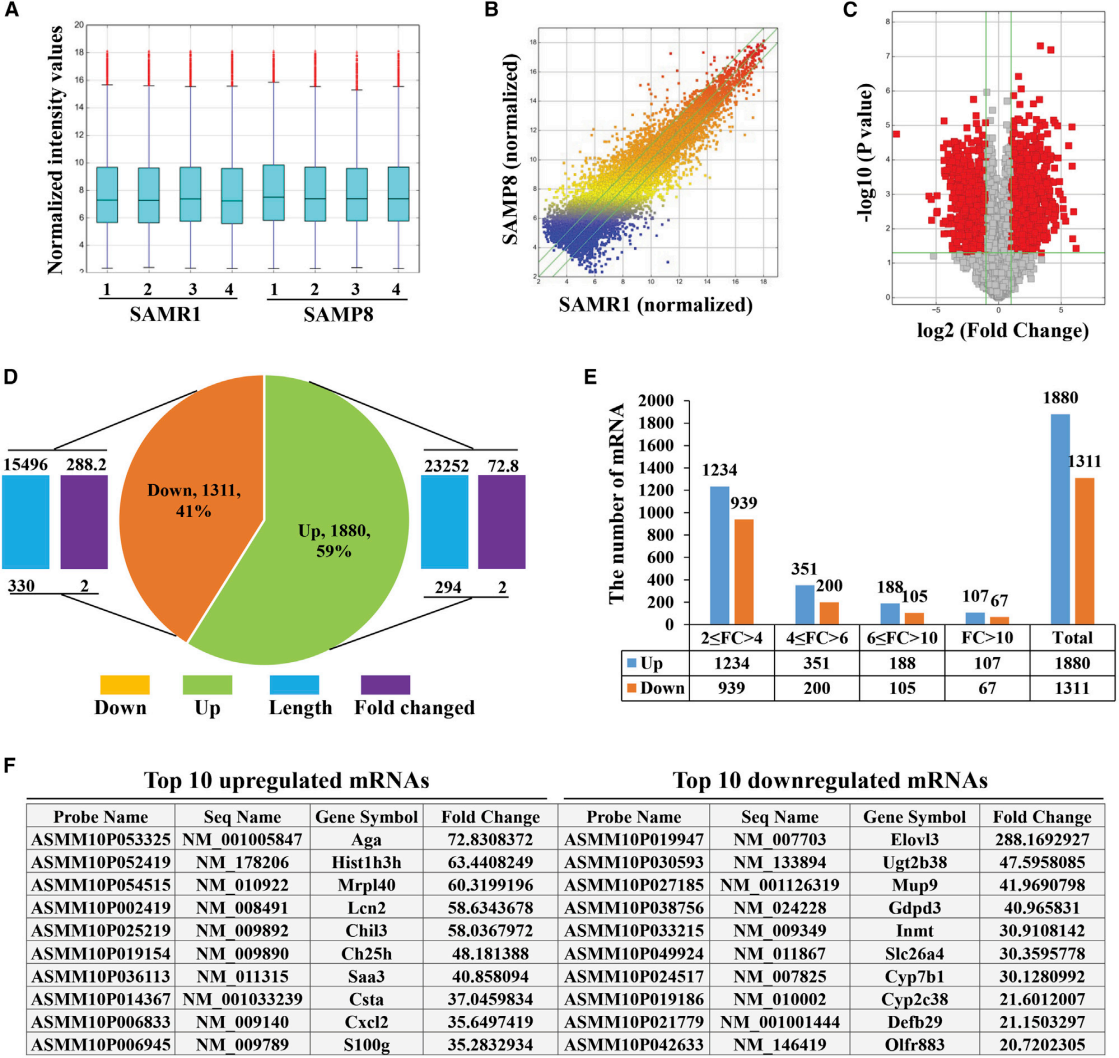
Differential Expression Profiles of mRNAs (A–C) Boxplot (A), scatterplot (B), and volcano plot (C) of the differentially expressed mRNAs in the two groups that were compared. (D) The pie chart shows the number of upregulated and downregulated mRNAs with the length and fold change. Green indicates upregulated mRNA, orange indicates downregulated mRNA, blue bar indicates length of mRNA, and brown bar indicates fold change of mRNA. (E) Number of mRNAs in the different subgroups classified by fold change (FC). Blue and orange bars indicate the number of upregulated and downregulated mRNAs, respectively. (F) Top 10 significantly upregulated and downregulated mRNAs in the microarray data.

A boxplot was generated to show the differential mRNA expression patterns among the hippocampal samples of SAMP8 and SAMR1 mice (Figure 3A). A scatterplot and volcano plot were used to visually assess the variation between the two groups (Figures 3B and 3C). The mRNA expression profile data from the microarray analysis contained a total of 17,922 mRNAs that were differentially expressed in the hippocampal tissues, of which 9,309 were upregulated and 8,613 were downregulated (Tables S4 and S5). When a fold change cutoff >2.0 and p value cutoff <0.05 were used, 1,880 mRNAs (the length and fold change ranged from 294 to 23,252 and 2 to 72.8, respectively) had upregulated expression, and 1,311 mRNAs (the length and fold change ranged from 330 to 15,496 and 2 to 228.2, respectively) displayed downregulated expression, among the 3,191 differentially expressed genes in SAMP8 mice compared with SAMR1 mice (Figure 3D; Table S6). Similar to the lncRNAs, most dysregulated mRNAs were in the 2 ≤ fold change < 4 subgroup (Figure 3E). In addition, the top 10 differentially regulated mRNAs were identified. The most obviously upregulated mRNA was Aga (NM_001005847; fold change, 72.8), while the most downregulated mRNA was Elovl3 (NM_007703; fold change, 288.2) (Figure 3F).

### GO Analysis: The Differential Expression of mRNAs in the Hippocampus of SAMP8 Mice Compared to SAMR1 Mice

**Figure 4:**
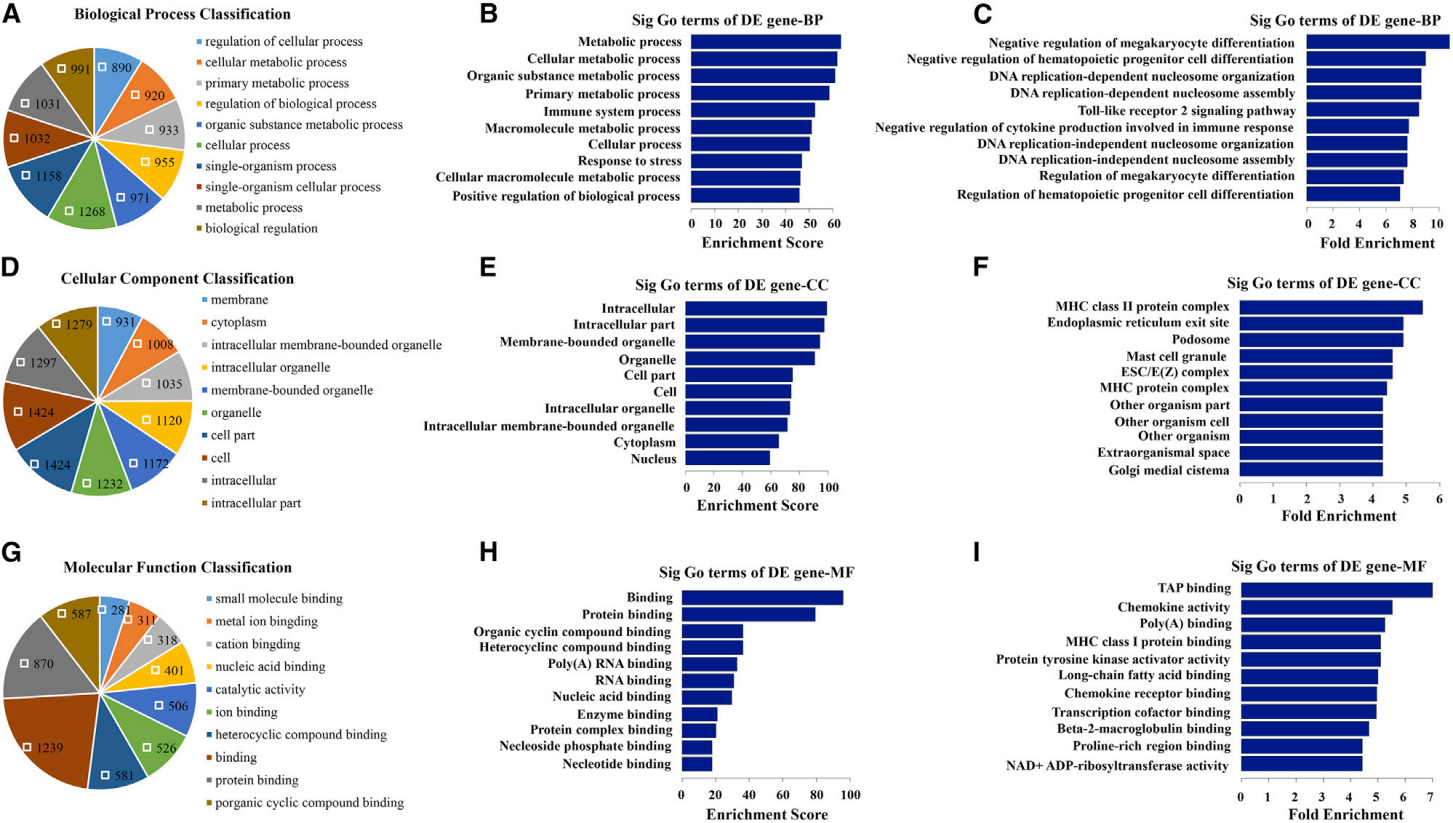
GO Analysis of Differentially Upregulated Expression of mRNAs (A, D, and G) Pie chart shows the top 10 significant enrichment terms. (B, E, and H) Bar plot shows the top 10 enrichment scores (−log_10_ (p value)). (C, F, and I) Bar plot shows the top 10 fold enrichment values ((count/pop. hits)/(list. total/pop. total)). “Count” stands for the number of DE genes associated with the listed ID of gene ontology term; “Pop.Hits” stands for the number of background population genes associated with the listed ID of gene ontology term; “List.Total” stands for the total number of DE genes; “Pop.Total” stands for the total number of background population genes. (A–C) biological process (BP). (D–F) Cellular component (CC). (G–I) Molecular function (MF).

**Figure 5:**
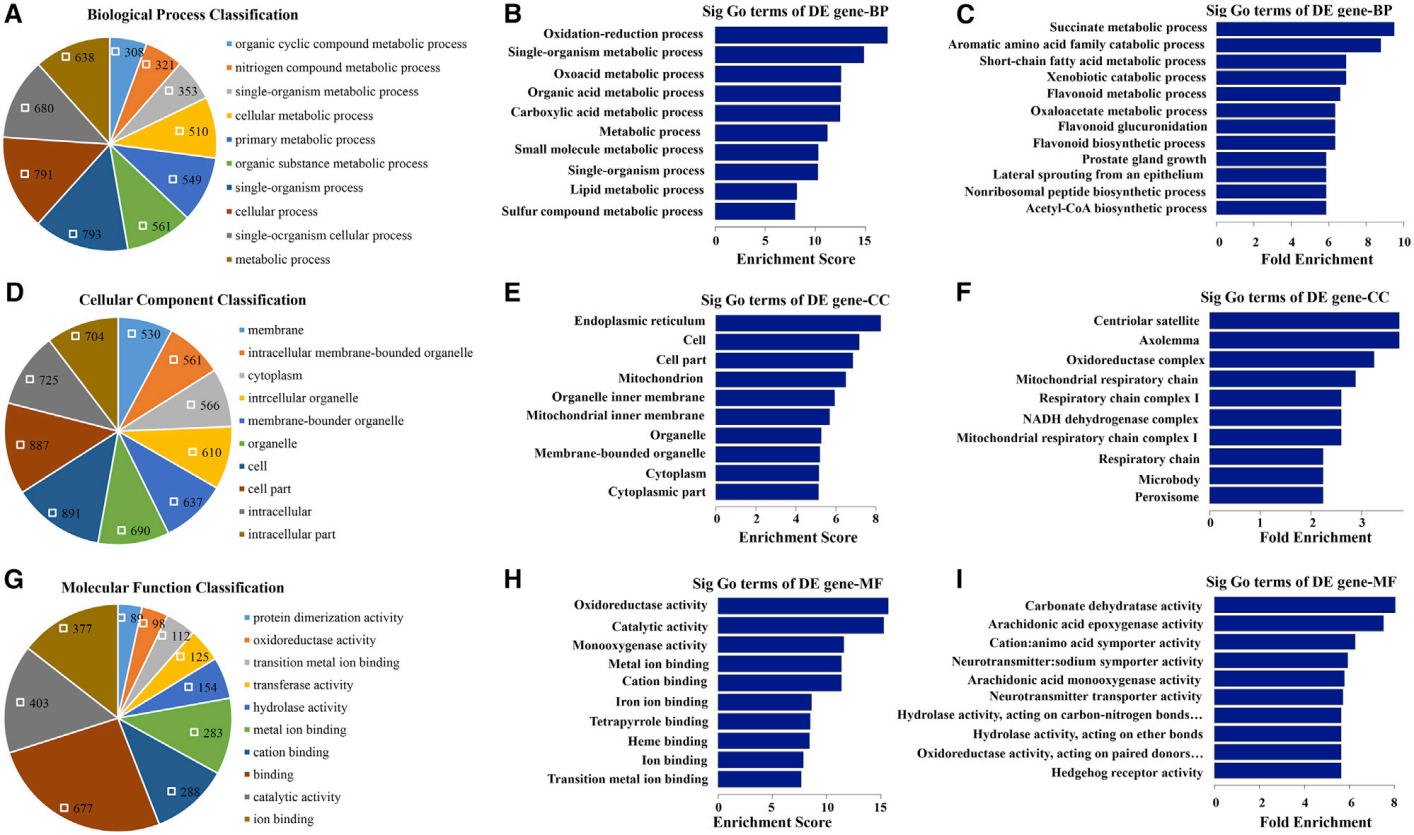
GO Analysis of Differentially Downregulated mRNAs (A, D, and G) Pie chart shows the top 10 significant enrichment terms. (B, E, and H) Bar plot shows the top 10 enrichment scores (−log_10_ (p value)). (C, F, and I) Bar plot shows the top 10 fold enrichment values ((count/pop. hits)/(list. total/pop. total)). “Count” stands for the number of DE genes associated with the listed ID of gene ontology term; “Pop.Hits” stands for the number of background population genes associated with the listed ID of gene ontology term; “List.Total” stands for the total number of DE genes; “Pop.Total” stands for the total number of background population genes. (A–C) Biological process (BP). (D–F) Cellular component (CC). (G–I) Molecular function (MF).

The Gene Ontology (GO) analysis covers three domains: biological process, cellular component, and molecular function. A Fisher’s exact test was used to determine whether there was more overlap between the differentially expressed gene list and the GO annotation list than would be expected by chance. The p value denotes the significance of GO term enrichment among the differentially expressed genes. As the p value decreases, the enrichment of the GO term becomes more significant (p ≤ 0.05 is recommended). GO analysis, including biological process, cellular component, and molecular function, for upregulated and downregulated mRNAs is shown in Tables S7, S8, S9, S10, S11, and S12. The pie chart shows the top 10 significant enrichment terms (Figures 4A, 4D, 4G, 5A, 5D, and 5G). The bar plot shows the top 10 enrichment scores (Figures 4B, 4E, 4H, 5B, 5E, and 5H) and fold enrichment (Figures 4C, 4F, 4I, 5C, 5F, and 5I) values of the enrichment terms. In our survey of existing data, we found that the GO terms of the upregulated mRNAs in the biological process category were mainly involved in (1) metabolic process, (2) cellular metabolic process, (3) organic substance metabolic process, (4) primary metabolic process, and (5) immune system process (Figure 4B). The GO terms of the downregulated mRNAs under biological process were mainly involved in (1) oxidation-reduction process, (2) single-organism metabolic process, (3) oxoacid metabolic process, (4) organic acid metabolic process, and (5) carboxylic acid metabolic process (Figure 5B). These results indicated that metabolic process reprogramming was likely related to AD.

### Pathway Analysis: Differentially Expressed mRNAs in the Hippocampus of SAMP8 Mice Compared to SAMR1 Mice

**Figure 6:**
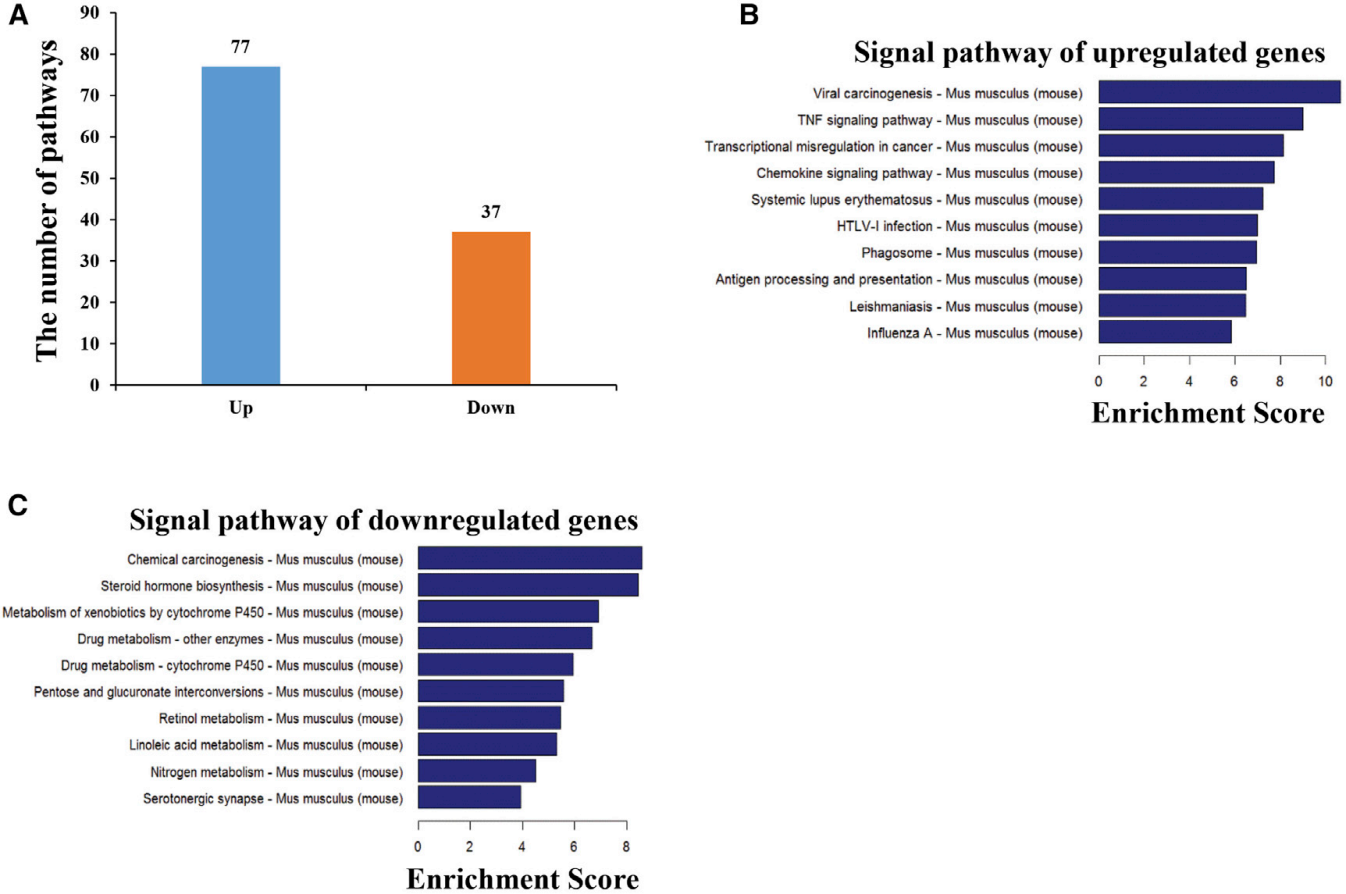
Pathway Analysis of mRNAs with Dysregulated Expression (A) Number of pathways of mRNAs with dysregulated expression. Pathway analysis mapped the genes to KEGG pathways. A p value ≥0.05 denotes the significance correlation of the pathway to the conditions. (B and C) Pathway analysis using KEGG for the differentially expressed transcripts and schematic diagrams of the two gene categories. Pathways corresponding to the upregulated transcripts (B) and pathways corresponding to the downregulated transcripts (C) are shown. The x and y axes represent the top 10 significantly enriched pathways and their scores (−log_10_ (p value)), respectively.

Pathway analysis is a functional analysis that maps genes to KEGG (Kyoto Encyclopedia of Genes and Genomes) pathways. The pathway analysis for upregulated and downregulated mRNAs is shown in Tables S13 and S14. The results showed that the numbers of upregulated and downregulated pathways were 77 and 37, respectively (Figure 6A; Tables S13 and S14). The bar plot shows the top 10 enrichment scores (−log_10_ (p value)) of the significantly enriched pathways (Figures 6B and 6C). Overall, we found that the upregulated mRNAs were mainly involved in the following pathways: (1) viral carcinogenesis, (2) tumor necrosis factor (TNF) signaling pathway, (3) transcriptional misregulation in cancer, (4) chemokine signaling pathway; and (5) systemic lupus erythematosus (Figure 6B). The downregulated mRNAs were mainly involved the following pathways: (1) chemical carcinogenesis, (2) steroid hormone biosynthesis, (3) metabolism of xenobiotics by cytochrome P450, (4) drug metabolism (other enzymes), and (5) drug metabolism (cytochrome P450) (Figure 6C), indicating that carcinogenesis, inflammation, and metabolism may be involved in AD pathogenesis. In addition, the upregulated and downregulated pathway maps of the pathways with the top 10 enrichment scores are shown in Figures S2–21.

### qRT-PCR Validates the Microarray Data of lncRNAs and mRNAs

**Figure 7:**
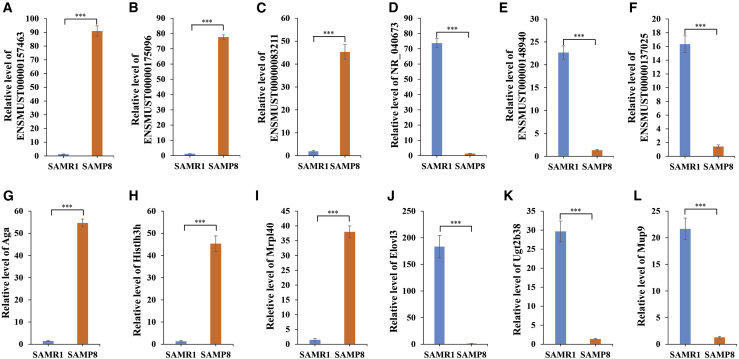
Validation of lncRNAs and mRNAs Using qRT-PCR (A–F) Comparison of the expression levels of lncRNAs between the microarray and qRT-PCR results. Three differentially upregulated (A–C) and downregulated (D–F) lncRNAs were validated by qRT-PCR. ∗∗∗p < 0.001. (G–L) Comparison of the expression levels of mRNAs between the microarray and qRT-PCR results. Three differentially upregulated (G–I) and downregulated (J–L) mRNAs were detected by qRT-PCR. The y axis represents the relative fold changes in expression across eight samples (SAMR1 = 4; SAMP8 = 4). ∗∗∗p < 0.001.

We confirmed the differential expression of lncRNAs and mRNAs that were identified in the microarray using qRT-PCR (Figure 7). For example, ENSMUST00000157463, and ENSMUST00000175096, ENSMUST00000083211 and NR_040673, ENSMUST00000148940, and ENSMUST00000137025 were the top three upregulated and downregulated lncRNAs, respectively, in the microarray data on the hippocampus of SAMP8 mice compared with SAMR1 mice (Table S3; Figure 2I). The qRT-PCR assay demonstrated the same expression patterns for the lncRNAs as for the microarray data (Figures 7A–7F). Similarly, we verified the expression patterns of the top three upregulated and downregulated mRNAs (upregulated, Aga, Hist1h3h, and Mrpl40; downregulated, Elovl3, Ugt2b38, and Mup9) from the differentially expressed mRNAs (Table S6; Figure 3F). The qRT-PCR results showed that the expression of Aga, Hist1h3h, and Mrpl40 was upregulated, and the expression of Elovl3, Ugt2b38, and Mup9 was downregulated, in the hippocampal samples of SAMP8 mice compared with SAMR1 mice (Figures 7G–7L). Thus, the results from the qRT-PCR assay and the microarray data analysis were consistent, supporting a strong correlation between the qRT-PCR results and the microarray data.

### Analysis of lincRNAs and Their Associated Nearby Coding Genes

**Figure 8:**
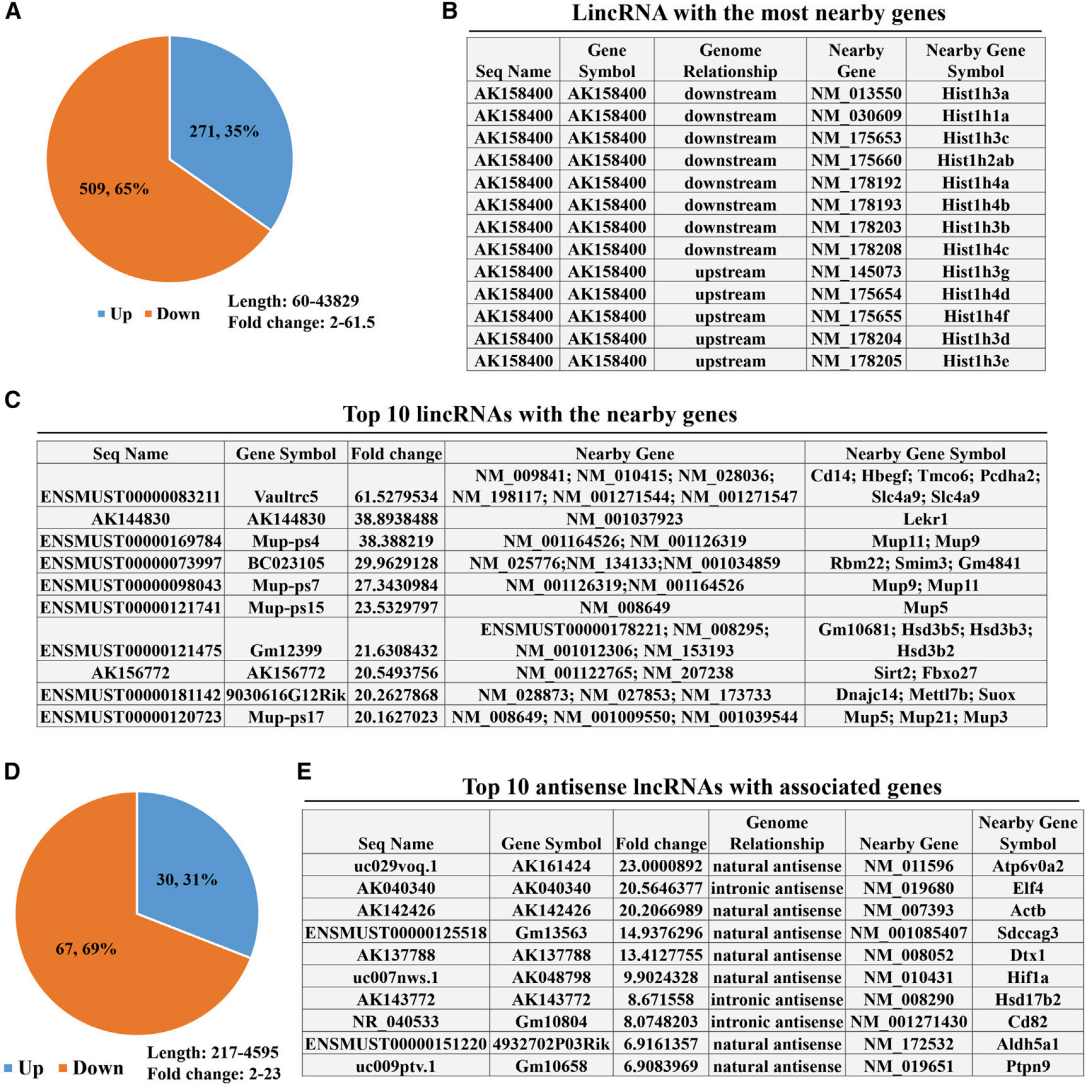
Analysis of lncRNAs and Their Nearby Coding Genes (A) Pie chart shows the number of lincRNAs (the length and fold change ranged from 60 to 43,829 and 2 to 61.5, respectively) that had nearby coding genes (<300 kb). The lincRNAs with nearby coding genes (distance between the lncRNA and coding gene <300 kb) were identified. Blue indicates upregulated lincRNAs, and orange indicates downregulated lincRNAs. (B) AK158400 had the most nearby coding genes. AK158400 had 13 nearby coding genes. (C) Top 10 lincRNAs (according to fold change) with their nearby coding genes. (D) Pie chart shows the number of antisense lncRNAs (the length and fold change ranged from 217 to 4,595 and 2 to 23, respectively) with associated coding genes. Blue indicates upregulated antisense lncRNAs, and orange indicates downregulated antisense lncRNAs. (E) Top 10 antisense lncRNAs (according to fold change) with their associated coding genes.

Because lincRNAs (long “intergenic” non-coding RNAs) play a key role in the regulation of nearby genes,^20^ we next analyzed the differentially expressed lncRNAs and their associated nearby coding genes (<300 kb). We found that a total of 780 lincRNAs (the length and fold change ranged from 60 to 43,829 and 2 to 61.5, respectively) had nearby associated coding genes, of which 271 lincRNAs were upregulated and 509 lincRNAs were downregulated (Figure 8A; Table S15). Among these lincRNAs, AK158400 had the most genes nearby (Figure 8B), all of which belonged to the histone cluster 1 family, suggesting that AK158400 may regulate the nucleosome structure of the chromosomal fiber by affecting nearby genes during AD progression. Furthermore, Figure 8C shows the top 10 lincRNAs (according to the fold change) and their associated nearby genes. Among lncRNAs, antisense lncRNAs have been studied in depth, and more than 30% of annotated human transcripts have corresponding antisense lncRNAs; these lncRNAs regulate the corresponding sense mRNA at the transcriptional or post-transcriptional level through a variety of mechanisms to exert their biological functions.^21^ We also focused on the differentially expressed antisense lncRNAs and their associated coding genes. We identified 97 differentially expressed human antisense lncRNAs (30 lncRNAs that were upregulated and 67 lncRNAs that were downregulated; the length and fold change ranged from 217 to 4,595 and 2 to 23, respectively) and their associated coding genes (Figures 8D and 8E; Table S16). In addition, the top 10 antisense lncRNAs and their associated coding genes are displayed in Figure 8E. All of these data will provide us with a new approach to understand AD pathogenesis in terms of AD-related lncRNAs and their associated nearby coding genes.

### E230001N04Rik Regulates Its Nearby Coding Genes Srpk1 and Fkbp5 Levels and Tau Level in AD

**Figure 9:**
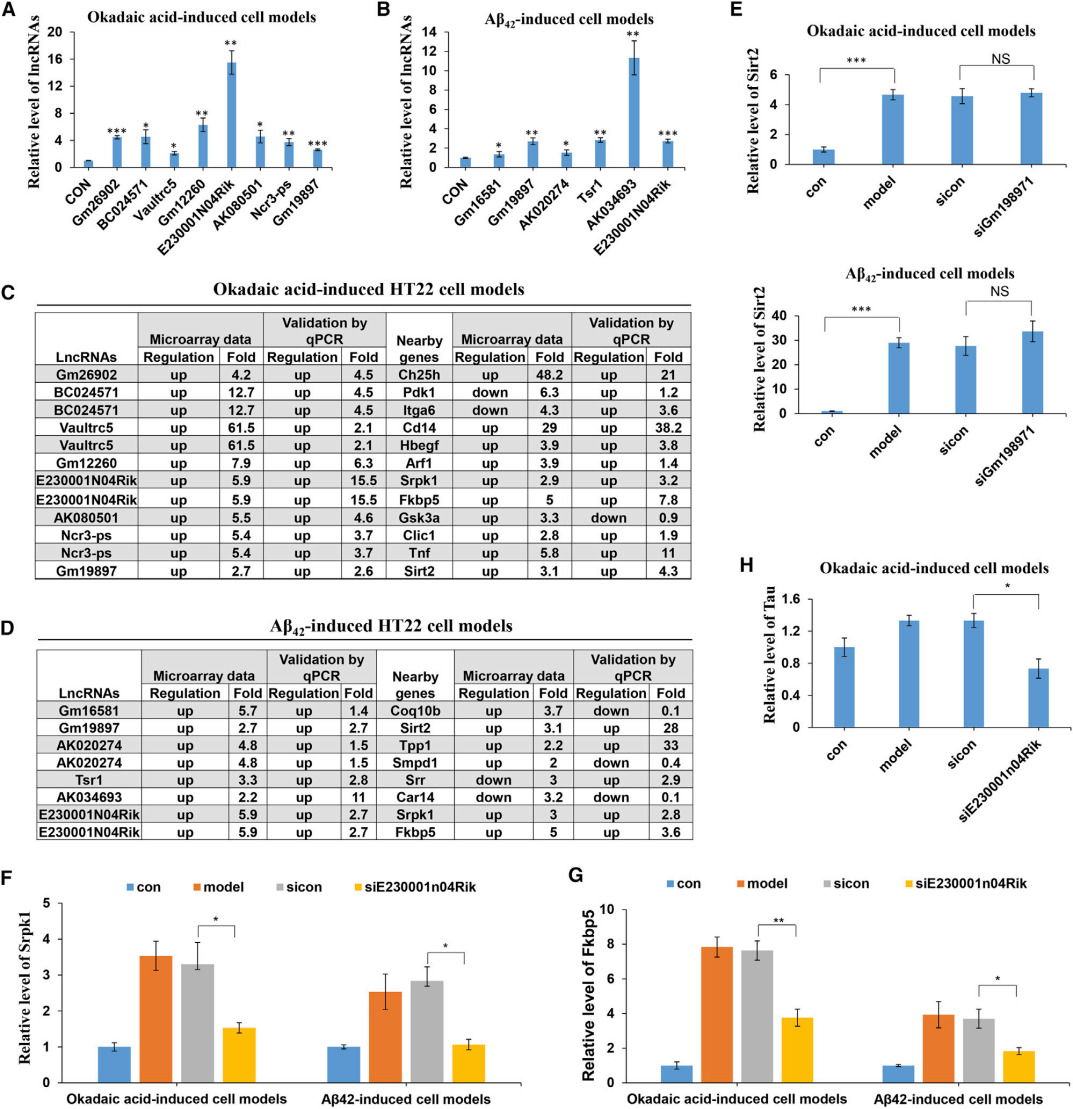
E230001N04Rik Regulates Its Nearby Coding Genes Srpk1 and Fkbp5 Levels and Tau Level in Alzheimer’s Disease (A and B) Expression patterns of lncRNAs in okadaic acid-induced (A) and Aβ42-induced (B) HT22 cell models. (C and D) Expression patterns of lncRNAs and their associated nearby coding genes in microarray data and two AD cell models by qRT-PCR validation. (C) Okadaic acid-induced HT22 cell models. (D) Aβ42-induced HT22 cell models. (E) The expression of Sirt2 in AD cell models by silence of Gm19897. (F and G) Expression of Srpk1 (F) and Fkbp5 (G) in AD cell models by silence of E230001N04Rik. (H) Expression of tau in okadaic acid-induced HT22 cell models by silence of E230001N04Rik. ∗p < 0.05, ∗∗p < 0.01, ∗∗∗p < 0.001. NS, not significant.

To evaluate the mechanism of specific lncRNAs and their associated nearby coding genes in AD progression, we first detected the expression patterns of lncRNAs and their associated nearby coding genes in two AD cell models (okadaic acid and Aβ_42_-induced HT22 cell models). As a result, the lncRNAs Gm26902, BC024571, Vaultrc5, Gm12260, E230001N04Rik, AK080501, and Ncr3-ps were upregulated in okadaic acid-induced HT22 cell models (Figure 9A). In Aβ_42_-induced HT22 cell models, the lncRNAs Gm16581, AK020274, Tsr1, AK034693, Gm19897, and E230001N04Rik were increased compared to control group (Figure 9B). We could see that E230001N04Rik and Gm19897 were upregulated in both two AD cell models, and the expression pattern of lncRNAs was consistent with microarray data (Figures 9C and 9D). We also detected the expression of those lncRNA-associated nearby coding genes and most of them were consistent with microarray data (Figures 9C and 9D). Second, we used the coding/non-coding gene co-expression network (CNC network) to predict the target genes of 12 lncRNAs validated in two AD cell models above. 11 lncRNAs and their target gene interactions are presented in Figure S22 and Table S17. AK034693 has not shown the predicted genes because the Pearson correlation coefficient between AK034693 and coding genes was less than 0.98. To our surprise, Srpk1 and Fkbp5, the associated nearby coding genes of E230001N04Rik, were positively correlated to E230001N04Rik in the CNC network; Sirt2, an associated nearby coding gene of Gm19897, was also positive correlated to Gm19897 (Figure S22; Table S17). To evaluate the exact effect of specific lncRNAs and their associated nearby coding genes in AD, we knocked down E230001N04Rik and Gm19897 by antisense oligonucleotides (ASOs) in AD cell models. The results showed that silencing Gm19897 did not affect the expression of nearby coding gene Sirt2 (Figure 9E), indicating that the role of Gm19897 on AD was not dependened on Sirt2. However, E230001N04Rik knockdown decreased the levels of its associated nearby coding genes Srpk1 and Fkbp5 (Figures 9F and 9G). Studies have shown that Srpk1 was increased in AD and regulated the production of tau protein, which predominantly contains four microtubule-binding repeats, which resulted in frontotemporal dementia;^22^^,^^23^ Fkbp5 interactions with Hsp90 promoted neurotoxic tau accumulation and increased tau stability and polymerized microtubules,^24^^,^^25^ indicating that E230001N04Rik might regulate tau protein accumulation by Srpk1 and/or Fkbp5 in AD progression. Indeed, E230001N04Rik knockdown also decreased tau level (Figure 9H). Taken together, our results show that E230001N04Rik regulated the tau level, which may occur through regulating its nearby coding genes Srpk1 and Fkbp5 in AD progression.

## Discussion

AD is a neurodegenerative disease characterized by devastating symptoms, such as apraxia, agnosia, aphasia, and emotional disturbance, because of progressive mental and behavioral functional decline.^26^ The SAM strain consists of SAMP and SAMR strains. The SAMP8 strain is a mouse model that shows early onset of learning and memory deficits during its aging process and was phenotypically selected from the AKR/J strain by Dr. T. Takeda’s laboratory at Kyoto University,^27^ whereas the SAMR1 strain shows a normal aging process,^28^ so it is usually used as an appropriate control for the SAMP8 strain. Many studies have shown that SAMP8 is a good model for studying age-related cognitive impairments and AD.^29^ The hippocampus is widely regarded as being at the center of the brain network that supports learning and memory and is the main impaired region of AD.^3^^,^^30^ Therefore, in the current study, we collected the hippocampus of SAMP8 and SAMR1 mice, but not the total brain, to comprehensively identify differentially expressed lncRNAs and their associated coding genes that may play an important role in AD pathogenesis. In the present study, we identified 3,112 differentially expressed lncRNAs and 3,191 differentially expressed mRNAs in SAMP8 mice compared to SAMR1 mice. The GO and KEGG analyses of AD-related transcripts could provide a foundation for future functional analysis. Furthermore, six lncRNAs and six mRNAs were selected for the further confirmation of the microarray results using quantitative PCR, and the results from quantitative PCR were consistent with the microarray findings. Moreover, we also analyzed lincRNAs and their nearby associated coding genes to examine the potential role of the regulation of the lincRNA-gene axis in AD pathogenesis. In addition, we also identified 97 aberrantly expressed antisense lncRNAs and their associated coding genes. All of these data will provide us with a new approach for understanding AD pathogenesis in terms of AD-related lncRNAs and their associated nearby coding genes.

Although AD-related genes and proteins have been extensively explored in recent decades, a detailed mechanism of AD is still lacking. Much is yet to be discovered about the precise biological changes that cause AD outside of genes and proteins. lncRNAs are a class of long RNAs (>200 nt)^31^ that are presumed to participate in many essential biological processes^32^ and human diseases, including cancer and AD.^33^^,^^34^ For example, lncRNA 51A overlaps with SORL1 and could affect Aβ formation.^14^^,^^15^ BC200 exhibited abnormal subcellular localization and expression levels in AD patients.^35^ BACE1-AS regulates BACE1 mRNA and protein expression and enhances APP processing and Aβ_1-42_.^12^^,^^13^ 17A, NDM29, and NAT-Rad18 were also reported to be involved in the progression of AD.^16^, ^17^, ^18^ However, with the exception of the few previously mentioned findings, our knowledge about the systematic expression profiles of lncRNAs and a comprehensive analysis of lncRNAs and their nearby coding genes in AD are still lacking. Although several studies have shown that lncRNAs are differentially expressed in 3xTg-AD^36^ or APP/PS1^37^ mice, a class of transgenic mice representing an uncommon familial form of AD, the roles of lncRNAs in AD remain largely unknown because none of the currently available models recapitulates all aspects of human AD.^38^ In our current study, we collected the hippocampus, the region of the brain that is considered to be mainly impaired in AD, but not the whole brain of SAMP8 and SAMR1 mice, mice that are regarded as good models for studying learning and memory, to comprehensively and systematically study lncRNAs in AD. Our results were more rigorous and accurate than those of Zhang et al.,^39^ who used the total brain to analyze the lncRNA expression profiles and deficiencies during an analysis of AD-related lncRNAs and their associated nearly coding genes. As a result, we identified 1,140 lncRNAs (the length and fold change ranged from 60 to 27,759 and 2 to 125.9, respectively) with upregulated expression, and 1,972 lncRNAs (the length and fold change ranged from 61 to 117,168 and 2 to 101.3, respectively) displayed downregulated expression, among the 3,112 differentially expressed lncRNAs in the hippocampus of SAMP8 mice compared with age-matched SAMR1 mice; these results were tremendously different from those of Zhang et al.^39^ in terms of mRNA expression profiles and GO and pathway analyses. These results suggested that AD pathogenesis is completely different in different regions, even in the same animal model, and the selection of which region to study deserves careful consideration.

In addition to lncRNA expression profiles, we also analyzed the aberrant mRNA expression profiles in the hippocampus of SAMP8. A total of 1,880 mRNAs had upregulated expression, and 1,311 mRNAs displayed downregulated expression, among the 3,191 differentially expressed genes in SAMP8 mice compared with SAMR1 mice; most of the dysregulated mRNAs were in the 2 ≤ fold change < 4 subgroup. GO analysis showed that metabolic process reprogramming was closely related to AD, which was also mentioned by Bredesen.^40^ In addition, the enrichment score values of the significantly correlated pathways of the top 10 downregulated mRNAs indicated that carcinogenesis, inflammation, and metabolism may be involved in AD pathogenesis, supporting an association between AD and cancer. Indeed, many studies have shown that cancer and AD do not often occur together, and this has been known for many years.^41^^,^^42^ Moreover, the results of the validation studies of lncRNAs and mRNAs by qRT-PCR were consistent with the microarray data, supporting the strong reliability of the microarray data in our study.

Another strength of the current study was the analysis of AD-related lincRNAs and their nearby coding genes. Some lincRNAs are known to play critical roles in diverse cellular processes through a variety of mechanisms.^43^ Although some lincRNA loci encode RNAs that act non-locally (in *trans*)^44^, there is emerging evidence that many lincRNA loci act locally (in *cis*) to regulate the expression of nearby genes.^45^^,^^46^ We found that a total of 780 lincRNAs had nearby associated coding genes, including both upstream and downstream genes. Among these lincRNAs, AK158400 had the most coding genes nearby (13 genes), all of which belonged to the histone cluster 1 family, suggesting that AK158400 might regulate the nucleosome structure of the chromosomal fiber by affecting nearby genes in AD progression; however, the role of AK158400 and its nearby coding genes in AD needs to be more carefully examined. Furthermore, antisense lncRNAs have been studied in depth, and more than 30% of the annotated human transcripts have corresponding antisense lncRNAs, which regulate the corresponding sense mRNA at the transcriptional or post-transcriptional level through a variety of mechanisms to exert their biological functions.^21^^,^^47^. In the current study, we identified 97 differentially expressed antisense lncRNAs and their associated coding genes, whose role in AD still needs further study. To evaluate the mechanism of specific lncRNAs and their associated nearby coding genes in AD progression, we detected the expression patterns of lncRNAs and their associated nearby coding genes in two AD cell models. In addition, we used the CNC network to predict the target genes of lncRNAs and found that E230001N04Rik and its associated nearby coding genes Srpk1 and Fkbp5 were positive correlated. What interested us was that Srpk1 and Fkbp5 as well as tau were downregulated when knocking down E230001N04Rik by antisense oligonucleotides in AD cell models. Studies have shown that Srpk1 regulated the production of tau protein, which resulted in frontotemporal dementia;^22^^,^^23^ Fkbp5 interacted with Hsp90 to promote neurotoxic tau accumulation and increased tau stability,^24^^,^^25^ indicating that E230001N04Rik regulated tau protein accumulation by Srpk1 and/or Fkbp5 in AD progression.

**Figure 10:**
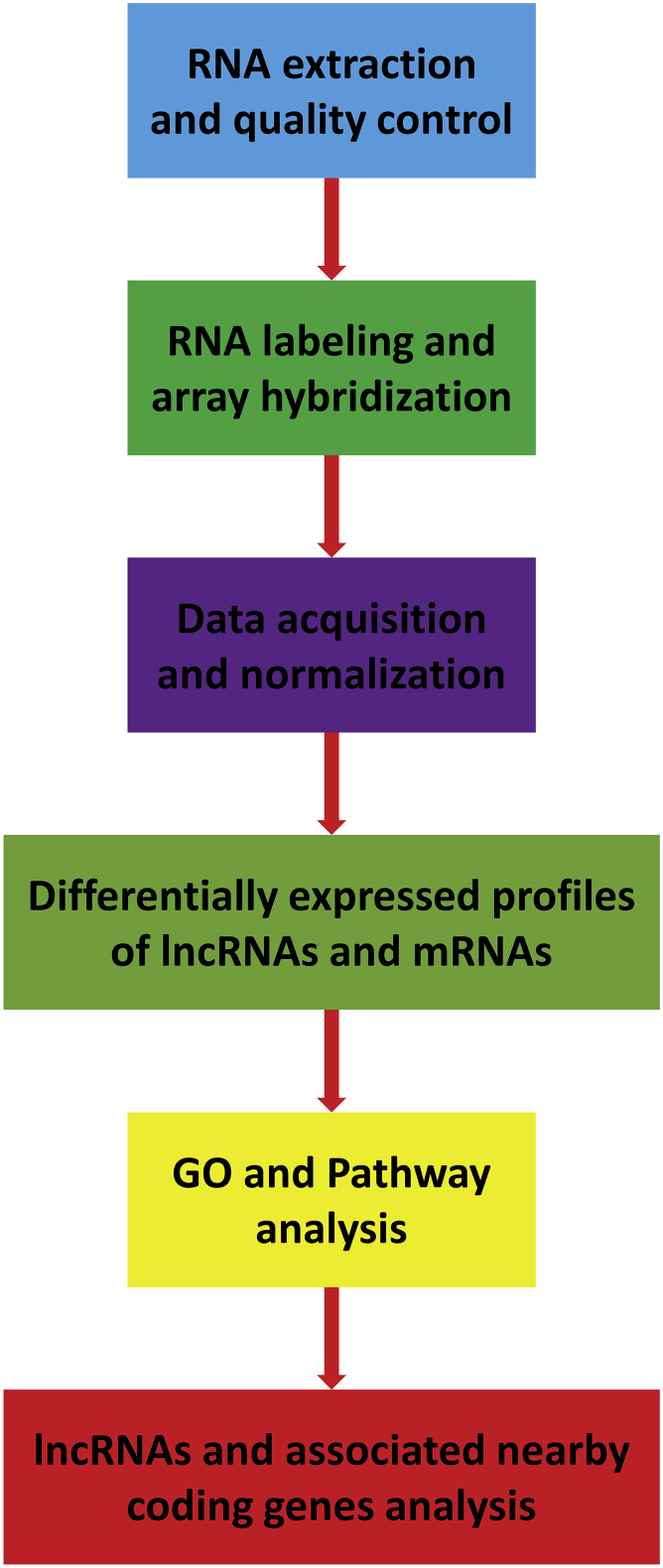
Flowchart of the Experiment

In conclusion, in the present study, we utilized microarray technology to systematically analyze the aberrant expression profiles of lncRNAs and mRNAs in the hippocampus of SAMP8 with AD. GO and pathway analyses further facilitated our understanding of the mechanism of AD. Moreover, the identification of lncRNAs and their associated nearby coding genes may contribute to the further study for understanding of AD pathogenesis and provide a valuable resource for the diagnosis and therapy of AD in the clinic (Figure 10).

## Materials and Methods

### Animal and Tissue Collection

SAMP8 mice and SAMR1 mice were purchased from Tianjin University of Traditional Chinese Medicine (Tianjin, China). The mice were housed with one mouse per cage in a standard environment (22°C ± 2°C, 45%–55% humidity, and a 12-h light/12-h dark cycle) and allowed to eat freely until they were 8 months old. Animals were randomly selected for the Morris water maze (MWM) test. We selected four animals from each group and gave them general anesthesia for the collection of hippocampal tissue. All animal protocols were carried out in strict accordance with the recommendations of the *Guide for the Care and Use of Laboratory Animals* from the National Institutes of Health. The project identification code is 20150301009, and approval date of the Ethics Committee is May 20, 2015.

### MWM Test

The learning and memory abilities of 8-month-old SAMP8 mice were evaluated using the MWM test.^48^^,^^49^ In brief, the objective for the mouse was to find the platform (8 cm in diameter), which was placed 1 cm below the water surface in the middle of one quadrant of the pool, 20 cm from the wall. Mice were given 1 day of adaptive training followed by trials on 5 consecutive days. Each trial started by gently placing the mouse into the water with its head toward the pool wall in any of the three quadrants without the platform. If an animal found the platform within 90 s, it was left on the platform for 30 s. If an animal did not find the platform, they were gently guided to the platform by the experimenter and left there for 30 s. On the last day of the MWM test, the platform was removed, and the number of times that the mice crossed the location of the original platform was recorded. Between trials, all mice were placed back in their home cages using a spoon-net to avoid direct contact with the experimenter. All trials were tracked automatically by a digital tracking system (Guangzhou Feidi Biological Technology) assessing path length, swimming speed, and escape latency.

### Arraystar lncRNA Array

#### RNA Extraction

Briefly, hippocampal tissues were pulverized and homogenized using a BioPulverizer (BioSpec) and Mini-Beadbeater-16 (BioSpec), respectively. The lysed cells were directly placed in a culture dish and 1 mL of TRIzol reagent was added (Invitrogen) to isolate the RNA. RNA quantification and quality control were performed with a NanoDrop ND-1000 (Thermo Fisher Scientific) and agarose gel electrophoresis.

#### Microarray

An Arraystar mouse lncRNA microarray v3.0 was designed for the global profiling of mouse lncRNAs and protein-coding transcripts. Approximately 35,923 lncRNAs and 24,881 coding transcripts could be detected by our third-generation lncRNA microarray.

#### RNA Labeling and Array Hybridization

Sample labeling and array hybridization were performed according to the Agilent one-color microarray-based gene expression analysis protocol (Agilent Technologies) with minor modifications. Briefly, mRNA was purified from total RNA after the removal of rRNA (mRNA-ONLY eukaryotic mRNA isolation kit, Epicenter Biotechnologies). Then, each sample was amplified and transcribed into fluorescent cRNA along the entire length of the transcripts without 3′ bias, utilizing a random priming method (Arraystar flash RNA labeling kit). The labeled cRNAs were purified with an RNeasy mini kit (QIAGEN). The concentration and specific activity of the labeled cRNAs (pmol of Cy3/μg of cRNA) were measured with a NanoDrop ND-1000. One microgram of each labeled cRNA was fragmented by adding 5 μL of 10× blocking agent and 1 μL of 25× fragmentation buffer and were then heated at 60°C for 30 min. Finally, 25 μL of 2× GE Healthcare hybridization buffer was added to dilute the labeled cRNA. Fifty microliters of hybridization solution was dispensed into the gasket slide and assembled onto the lncRNA expression microarray slide. The slides were incubated for 17 h at 65°C in an Agilent hybridization oven. The hybridized arrays were washed, fixed, and scanned using an Agilent DNA microarray scanner (part no. G2505C).

### lncRNA Microarray Data Analysis

The Agilent feature extraction software (version 11.0.1.1) was used to analyze the acquired array images. Quantile normalization and subsequent data processing were performed using the GeneSpring GX v12.1 software package (Agilent Technologies). In brief, quantile normalization was performed by the following steps: (1) The expression values of each sample were sorted in ascending order and placed next to each other. (2) Each column was sorted in ascending order. The mean of the sorted order across all samples was taken. Thus, each row in this sorted matrix had a value equal to the previous mean. (3) The modified matrix as obtained in the previous step was rearranged to have the same ordering as the input matrix. After quantile normalization of the raw data, lncRNAs and mRNAs that in at least four out of the eight samples were flagged as present or marginal (“all targets value”) were chosen for further data analysis. Differentially expressed lncRNAs and mRNAs that were significantly different between the two groups were identified through p value/false discovery rate (FDR) filtering. lncRNAs and mRNAs that were differentially expressed between the two samples were identified through fold change filtering. For the multiple test correction, we used the Benjamini-Hochberg method. Hierarchical clustering and combined analysis were performed using original scripts from our laboratory.

### qRT-PCR

The results of the lncRNA and mRNA expression profiles were validated by qRT-PCR. Total mRNA was extracted with TRIzol reagent (Invitrogen), and the concentration was measured with a NanoDrop 2000. Reverse transcription to generate cDNA was performed by using Takara 5× PrimeScript RT master mix. qRT-PCR was performed according to the manufacturer’s instructions. The specific quantitative primers for the validation of lncRNAs and mRNAs and the detailed protocol for qRT-PCR are described in Hong et al.^50^ and Supplemental Materials and Methods.

### GO and KEGG Analyses

#### GO Analysis

The GO project provides a controlled vocabulary to describe gene and gene product attributes in any organism (http://www.geneontology.org/). GO enrichment analysis was used to identify differentially expressed genes by using topGO.^51^ Fisher’s exact test was used to determine whether there was more overlap between the differentially expressed gene list and the GO annotation list than would be expected by chance. The p value denotes the significance of the GO term enrichment in the differentially expressed genes. As the p value decreases, the significance of the GO term also increases (p ≤ 0.05 is recommended).

#### KEGG Analysis

Pathway analysis is a functional analysis that maps genes to KEGG pathways. The p value (EASE score, Fisher p value, or hypergeometric p value) denotes the significance of correlation of the pathway to the conditions. As the p value decreases, the more the significance of the pathway correlation also increases (the recommend p value cutoff is 0.05).

### lncRNA-mRNA Co-expression Network

For lncRNA-mRNA correlation analysis, we calculated the Pearson correlation of lncRNA expression value with that of each mRNA to identify significantly co-expressed lncRNAs and mRNAs with the Pearson correlation coefficient ≥0.98. A CNC network was constructed based on the positive or negative correlation analysis between differentially expressed lncRNAs and mRNAs by using the open-source bioinformatics software Cytoscape.

### AD Cell Models and Knockdown of lncRNAs by antisense oligonucleotide (ASO)

For the okadaic acid-induced HT22 cell model, HT22 cells were cultured and treated with 30 μM okadaic acid for 36 h. For the Aβ42-induced HT22 cell model, HT22 cells were treated with 20 μM Aβ42 for 24 h. For the lncRNA knockdown assay, HT22 cells were transfected with antisense oligonucleotides (purchased from RiboBio, Guangzhou, China) for 24 h and then the RNA was collected for qRT-PCR.

### Statistical Analysis

The statistical significance was analyzed using SPSS (version 22, IBM, Armonk, NY, USA). All data are shown as the means ± SEM. p < 0.05 was considered significant. Briefly, a Student’s t test was used to compare the qRT-PCR results. Behavioral data from the training period were first assessed for normality and sphericity using the Shapiro-Wilks test and Mauchly’s test, respectively, and were then analyzed using repeated-measures ANOVA. Statistical significance was set at p < 0.05.

## Author Contributions

Shuhuan Fang, Qi Wang and Jiansong Fang conceived and designed the project. Honghai Hong, Yousheng Mo and Dongli Li performed and conducted the experiments. Honghai Hong wrote the manuscript. Zhiheng Xu, Yanfang Liao, Ping Yin, Xinning Liu and Yong Xia contributed to the writing of the manuscript.

## Conflicts of Interest

The authors declare no competing interests.

## Supplemental Information

Document S1. Supplemental Materials and Methods and Figures S1–S22

Table S1. All Comparison of lncRNAs

Table S2. lncRNA Expression Profiling Data

Table S3. Differentially Expressed lncRNAs

Table S4. All Comparisons of mRNAs

Table S5. mRNA Expression Profiling Data

Table S6. Differentially Expressed mRNAs

Table S7. Upregulated Biological Process (BP) Results (Mouse)

Table S8. Upregulated Cellular Component (CC) Results (Mouse)

Table S9. Upregulated Molecular Function (MF) Results (Mouse)

Table S10. Downregulated BP Results (Mouse)

Table S11. Downregulated CC Results (Mouse)

Table S12. Downregulated MF Results (Mouse)

Table S13. Upregulated Pathway Results

Table S14. Downregulated Pathway Results

Table S15. lincRNAs and Their Nearby Coding Gene Data

Table S16. Antisense lncRNAs and Their Associated Coding Gene Data

Table S17. CNC Network Pairs

Document S2. Article plus Supplemental Information
