# Supplementary material for: Aberrant Expression Profiles of lncRNAs and Their Associated Nearby Coding Genes in the Hippocampus of the SAMP8 Mouse Model with AD
Source: Mol Ther Nucleic Acids. 2020 Feb 19;20:140–54. doi: 10.1016/j.omtn.2020.02.008 (PMC7066064; doi:10.1016/j.omtn.2020.02.008)
Supplement: Document S1. Supplemental Materials and Methods and Figures S1–S22 [file mmc1.pdf]

OMTN, Volume *20*

Supplemental Information

Aberrant Expression Profiles of lncRNAs and Their Associated Nearby Coding Genes in the Hippocampus of the SAMP8 Mouse Model with AD

Honghai Hong, Yousheng Mo, Dongli Li, Zhiheng Xu, Yanfang Liao, Ping Yin, Xinning Liu, Yong Xia, Jiansong Fang, Qi Wang, and Shuhuan Fang

Supplemental information

Materials and methods

qRT-PCR

Total mRNA was extracted by TRIzol Reagent (Invitrogen life technologies) and the concentration was measure by nanodrop2000 and reversed transcription to cDNA by Takara 5×Primer Script RT Master Mix. qRT-PCR was performed by Bio-rad CFX384 and TOYOBO 2×SYBR is used. The expression of lncRNAs and mRNAs were measured by the Bio-rad CFX manager and GAPDH was used as reference. Primer sequences were described in table 1. Normalized Fold Expression: $\triangle\triangle CQ = 2^{(-\triangle CQ)}$, $\triangle CQ = CQ_{target} - CQ_{GAPDH}$

Table1. Primer sequence for lncRNAs and mRNAs

Seq Name	Gene Symbol	Forward primer	Reverse primer
ENSMUST00000157463	Rmrp	5'GCTCTGAAGGCCTGTTTCCT-3'	5'GACTTGCGGGGGAAGTCTAC-3'
ENSMUST00000175096	Rpph1	5'CGGAGCTTGGAACAGACTCA-3'	5'GAATTGGGTTATGAGGCCCG-3'
ENSMUST00000083211	Vaultrc5	5'GCTCAGCGGTTACTTCGACA-3'	5'CGGGTTAGGTAAGTGGTTGGT-3'
NR_040673	Gm12718	5'TCAAGGGTGCAGTCCATCAC-3'	5'TGCACAATCACCCAGGAGTC-3'
ENSMUST00000148940	Mup2	5'ACCTATCCAATGCCAATCGCT-3'	5'GATATTGCCCACCTGGAGGC-3'
ENSMUST00000137025	Mup9	5'AGACGTGGTCCTGACAGACA-3'	5'AGGGTCAGTCCCAAACACAG-3'
NM_001005847	Aga	5'-CCTATGCTGATGACACGGCT-3'	5'-CTACAGCTTGGTAGCTCGGC-3'
NM_178206	Hist1h3h	5'-TTGGAAGCTCGGGTGTTACC-3'	5'-GTAGCGGTGAGGCTTCTTCA-3'
NM_010922	Mrpl40	5'-AAATCAAGACAGCGACCTCA-3'	5'-GCTGGGCTTCTAGCATGGAT-3'
NM_007703	Elovl3	5'-TGCCATCTACACGGATGACG-3'	5'-CCGTGTCTCCCAGTTCAACAA-3'
NM_133894	Ugt2b38	5'ATATTTGGGAAGGCCCACCA-3'	5'AGCAGGTTTGCAGTGGAGTC-3'
NM_001126319	Mup9	5'GGCTGGTGAATATTCTGTGACG-3'	5'GTTCTCGGCCATAGAGCCC-3'

Supplemental Table

Table S1. All Comparison LncRNAs

Table S2. LncRNA Expression Profiling Data

Table S3. Differentially Expressed LncRNAs

Table S4. All Comparison mRNAs

Table S5. mRNA Expression Profiling Data

Table S6. Differentially Expressed mRNAs

Table S7. Upregulated_BP_result(Mouse)

Table S8. Upregulated_CC_result(Mouse)

Table S9. Upregulated_MF_result(Mouse)

Table S10. Downreulated_BP_result(Mouse)

Table S11. Downreulated_CC_result(Mouse)

Table S12. Downreulated_MF_result(Mouse)

Table S13. Upregulated_Pathway Result

Table S14. Downregulated_Pathway Result

Table S15. LincRNAs and their nearby coding gene data

Table S16. Antisense lncRNAs and their associated coding gene data

Table S17. CNC network pairs

Supplemental figure legends

Figure S1. The number of significantly dysregulated lncRNAs in different databases

(A and B) The number of up- (A) and downregulated expression of lncRNAs in authoritative databases. The lncRNAs are carefully collected from the most authoritative databases such as such as RefSeq, UCSC_knowngene, Ensembl, UCR, lincRNA and many related literatures.

Figure S2-21. The top ten enrichment score pathway maps of up- and downregulated mRNAs

(S2-21) pathway maps of Viral carcinogenesis (S2), TNF signaling pathway (S3), Transcriptional misregulation in cancer (S4), Chemokine signaling pathway (S5), Systemic lupus erythematosus (S6), HTLV-I infection (S7), Phagosome (S8), Antigen processing and presentation (S9), Leishmaniasis (S10), Influenza A (S11), Chemical carcinogenesis (S12), Steroid hormone biosynthesis (S13), Metabolism of xenobiotics by cytochrome P450 (S14), Drug metabolism-other enzymes (S15), Drug metabolism-cytochrome P450 (S16), Pentose and glucuronate interconversions (S17), Retinol metabolism (S18), Linoleic acid metabolism (S19), Nitrogen metabolism (S20) and Serotonergic synapse (S21).Yellow marked nodes are associated with down-regulated genes, orange marked nodes are associated with up-regulated or only whole dataset genes, green nodes have no significance.

Figure S22. CNC network

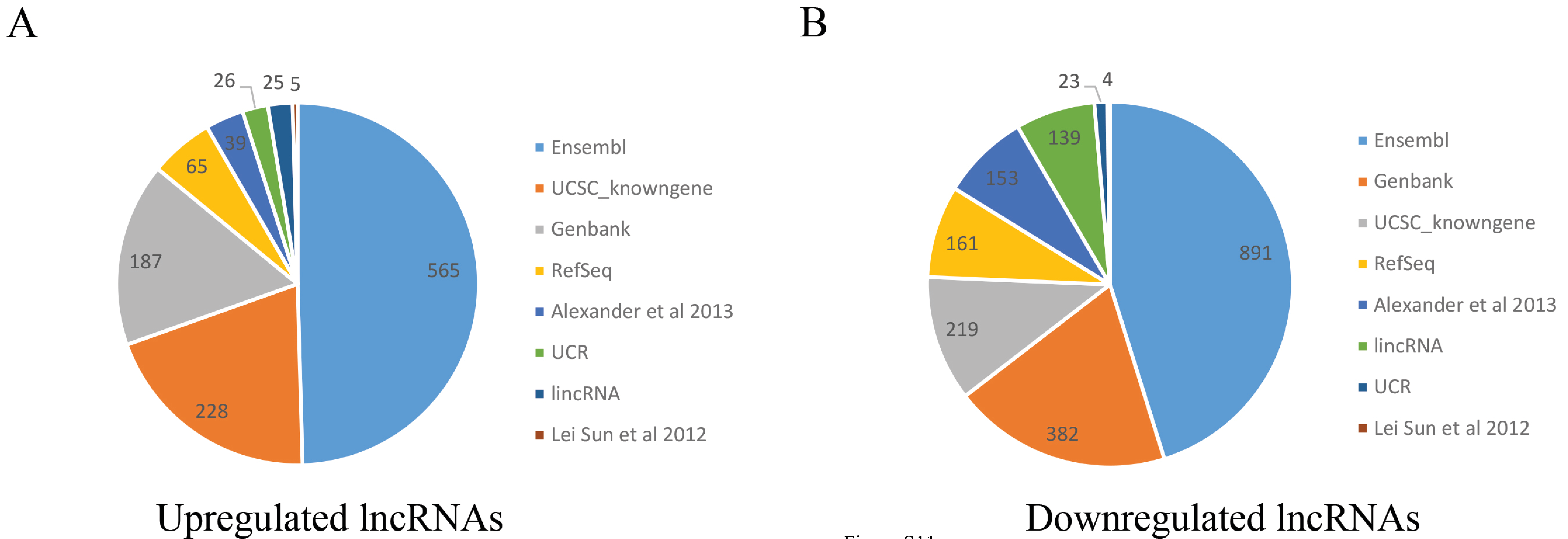
A
26
25
5
39
65
187
565
228
Ensembl
UCSC_knowngene
Genbank
RefSeq
Alexander et al 2013
UCR
lincRNA
Lei Sun et al 2012
Upregulated lncRNAs
B
23
4
139
153
161
891
219
382
Ensembl
Genbank
UCSC_knowngene
RefSeq
Alexander et al 2013
lincRNA
UCR
Lei Sun et al 2012
Downregulated lncRNAs

Figure S11

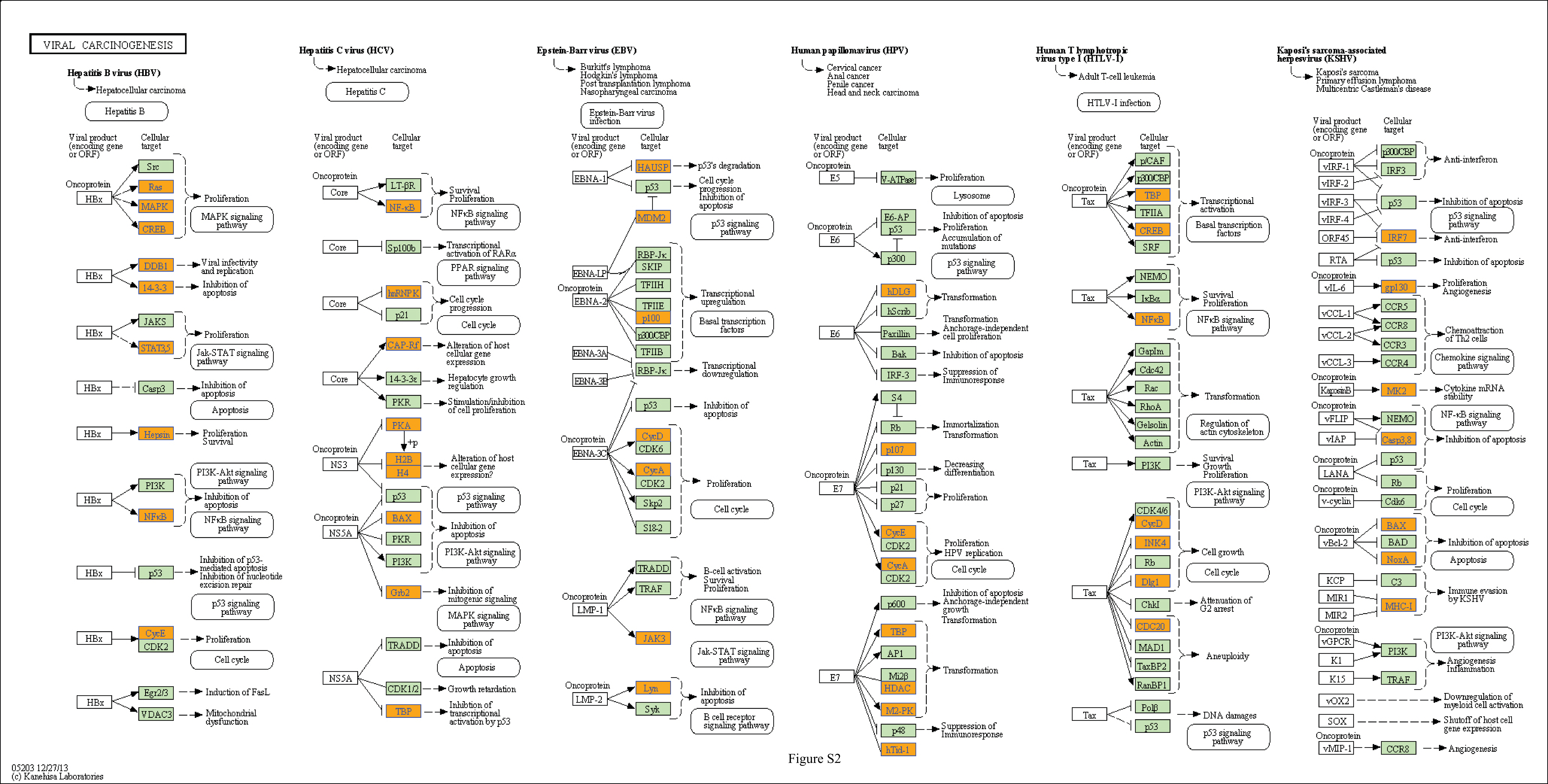
VIRAL CARCINOGENESIS
Hepatitis B virus (HBV)
Hepatocellular carcinoma
Hepatitis B
Viral product (encoding gene or ORF)
Cellular target
Oncoprotein
HBx
Src
Ras
MAPK
CREB
Proliferation
MAPK signaling pathway
DDB1
14-3-3
Viral infectivity and replication
Inhibition of apoptosis
JAK3
STAT3,5
Jak-STAT signaling pathway
Casp3
Apoptosis
Hepsin
Proliferation Survival
PI3K-Akt signaling pathway
PI3K
NFκB
NFκB signaling pathway
p53
Inhibition of p53-mediated apoptosis
Inhibition of nucleotide excision repair
p53 signaling pathway
CycE
CDK2
Cell cycle
Egr2/3
VDAC3
Induction of FasL
Mitochondrial dysfunction
Hepatitis C virus (HCV)
Hepatitis C
Core
LT-βR
NF-κB
Survival Proliferation
NFκB signaling pathway
Sp100b
Transcriptional activation of RARα
PPAR signaling pathway
hnRNPK
p21
Cell cycle progression
CAP-Rf
14-3-3ε
PKR
Alteration of host cellular gene expression
Hepatocyte growth regulation
Stimulation/inhibition of cell proliferation
NS3
PKA
+p
H2B
H4
Alteration of host cellular gene expression?
NS5A
BAX
Inhibition of apoptosis
Grb2
Inhibition of mitogenic signaling
TRADD
CDK1/2
Growth retardation
TBP
Inhibition of transcriptional activation by p53
Epstein-Barr virus (EBV)
Burkitt's lymphoma
Hodgkin's lymphoma
Post transplantation lymphoma
Nasopharyngeal carcinoma
Epstein-Barr virus infection
EBNA-1
HAUSP
p53's degradation
Cell cycle progression
Inhibition of apoptosis
MDM2
EBNA-LP
EBNA-2
RBP-Jκ
SKIP
TFIIH
TFIIE
p100
p300/CBP
TFIIB
Transcriptional upregulation
Basal transcription factors
EBNA-3A
EBNA-3B
Transcriptional downregulation
EBNA-3C
CycD
CDK6
CycA
Skp2
S18-2
LMP-1
TRAF
B-cell activation Survival Proliferation
JAK3
LMP-2
Lyn
Syk
B cell receptor signaling pathway
Human papillomavirus (HPV)
Cervical cancer
Anal cancer
Penile cancer
Head and neck carcinoma
E5
V-ATPase
Lysosome
E6
E6-AP
p300
Inhibition of apoptosis Proliferation
Accumulation of mutations
hDLG
hScrib
Transformation
Paxillin
Transformation Anchorage-independent cell proliferation
Bak
IRF-3
Suppression of Immunoresponse
E7
S4
Rb
Immortalization Transformation
p107
p130
Decreasing differentiation
p27
Proliferation HPV replication
p600
Inhibition of apoptosis Anchorage-independent growth Transformation
AP1
Mi2β
HDAC
M2-PK
p48
hTid-1
Human T lymphotropic virus type 1 (HTLV-1)
Adult T-cell leukemia
HTLV-I infection
Tax
p/CAF
SRF
Transcriptional activation
NEMO
IκBα
Survival Proliferation
GapIm
Cdc42
Rac
RhoA
Gelsolin
Actin
Regulation of actin cytoskeleton
Survival Growth Proliferation
CDK4/6
INK4
Dlg1
Cell growth
ChkI
Attenuation of G2 arrest
CDC20
MAD1
TaxBP2
RanBP1
Aneuploidy
Polβ
DNA damages
Kaposi's sarcoma-associated herpesvirus (KSHV)
Kaposi's sarcoma
Primary effusion lymphoma
Multicentric Castleman's disease
vIRF-1
vIRF-2
vIRF-3
vIRF-4
ORF45
IRF3
IRF7
Anti-interferon
RTA
vIL-6
gp130
Proliferation Angiogenesis
vCCL-1
vCCL-2
vCCL-3
CCR5
CCR8
CCR3
CCR4
Chemoattraction of Th2 cells
Chemokine signaling pathway
KaposinB
MK2
Cytokine mRNA stability
vFLIP
vIAP
Casp3,8
NF-κB signaling pathway
LANA
v-cyclin
Cdk6
vBcl-2
BAD
NoxA
KCP
MIR1
MIR2
C3
MHC-I
Immune evasion by KSHV
vGPCR
K1
K15
Angiogenesis Inflammation
vOX2
Downregulation of myeloid cell activation
SOX
Shutoff of host cell gene expression
vMIP-1
05203 12/27/13
(c) Kanehisa Laboratories
Figure S2

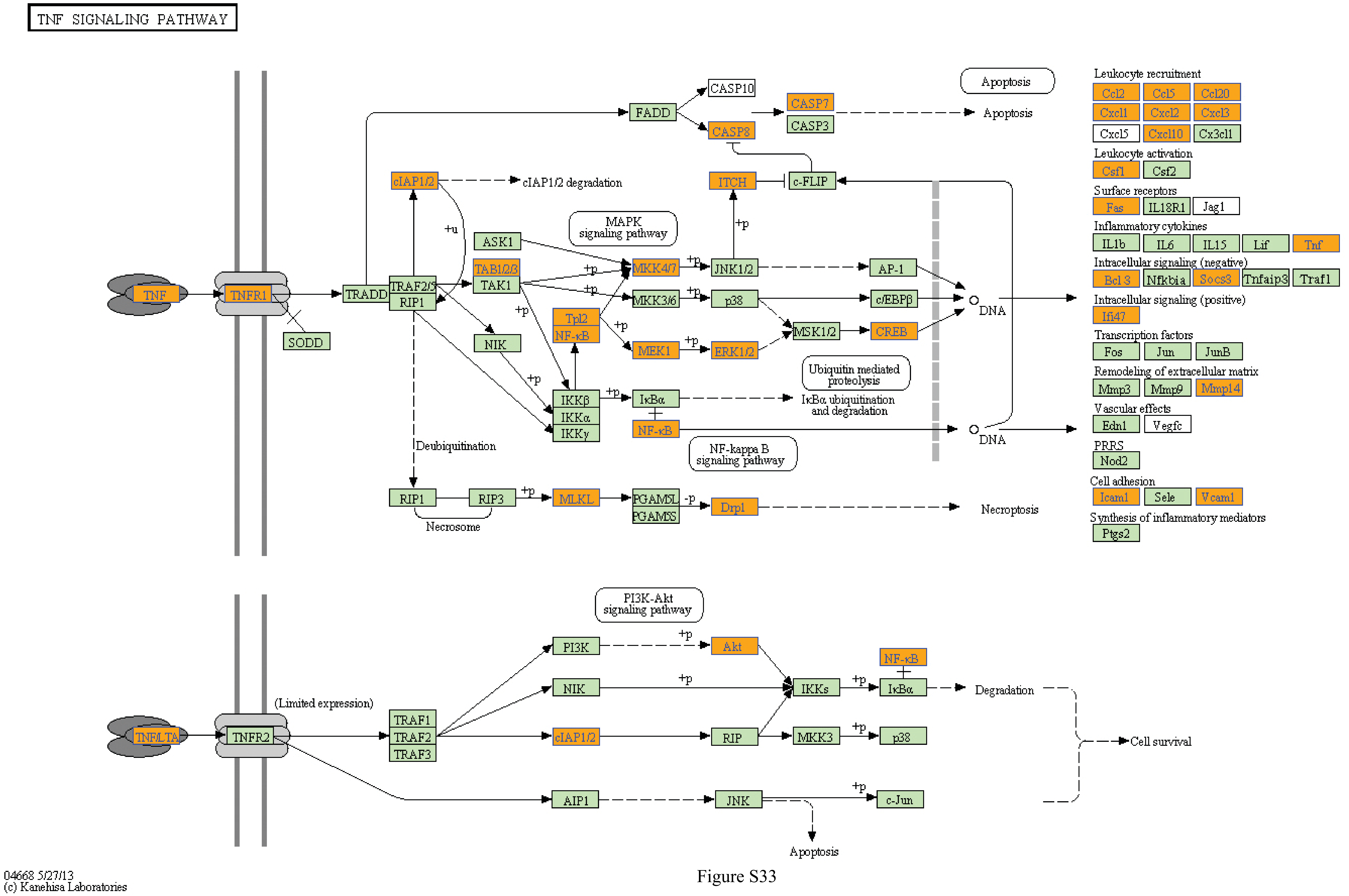
TNF SIGNALING PATHWAY
TNF
TNFR1
SODD
TRADD
TRAF2/5
RIP1
cIAP1/2
cIAP1/2 degradation
+u
ASK1
TAB1/2/3
TAK1
NIK
MAPK signaling pathway
FADD
CASP10
CASP8
CASP7
CASP3
Apoptosis
ITCH
c-FLIP
MKK4/7
MKK3/6
Tpl2
NF-κB
MEK1
JNK1/2
p38
ERK1/2
MSK1/2
AP-1
c/EBPβ
CREB
DNA
IKKβ
IKKα
IKKγ
IκBα
Ubiquitin mediated proteolysis
IκBα ubiquitination and degradation
NF-kappa B signaling pathway
Deubiquitination
RIP3
MLKL
PGAM5L
PGAM5S
-p
Drp1
Necroptosis
Necrosome
+p
Leukocyte recruitment
Ccl2
Ccl5
Ccl20
Cxcl1
Cxcl2
Cxcl3
Cxcl5
Cxcl10
Cx3cl1
Leukocyte activation
Csf1
Csf2
Surface receptors
Fas
IL18R1
Jag1
Inflammatory cytokines
IL1b
IL6
IL15
Lif
Tnf
Intracellular signaling (negative)
Bcl3
Nfkbia
Socs3
Tnfaip3
Traf1
Intracellular signaling (positive)
Ifi47
Transcription factors
Fos
Jun
JunB
Remodeling of extracellular matrix
Mmp3
Mmp9
Mmp14
Vascular effects
Edn1
Vegfc
PRRS
Nod2
Cell adhesion
Icam1
Sele
Vcam1
Synthesis of inflammatory mediators
Ptgs2
PI3K-Akt signaling pathway
TNF/LTA
TNFR2
(Limited expression)
TRAF1
TRAF2
TRAF3
PI3K
Akt
IKKs
RIP
MKK3
AIP1
JNK
c-Jun
Degradation
Cell survival
04668 5/27/13
(c) Kanehisa Laboratories

Figure S33

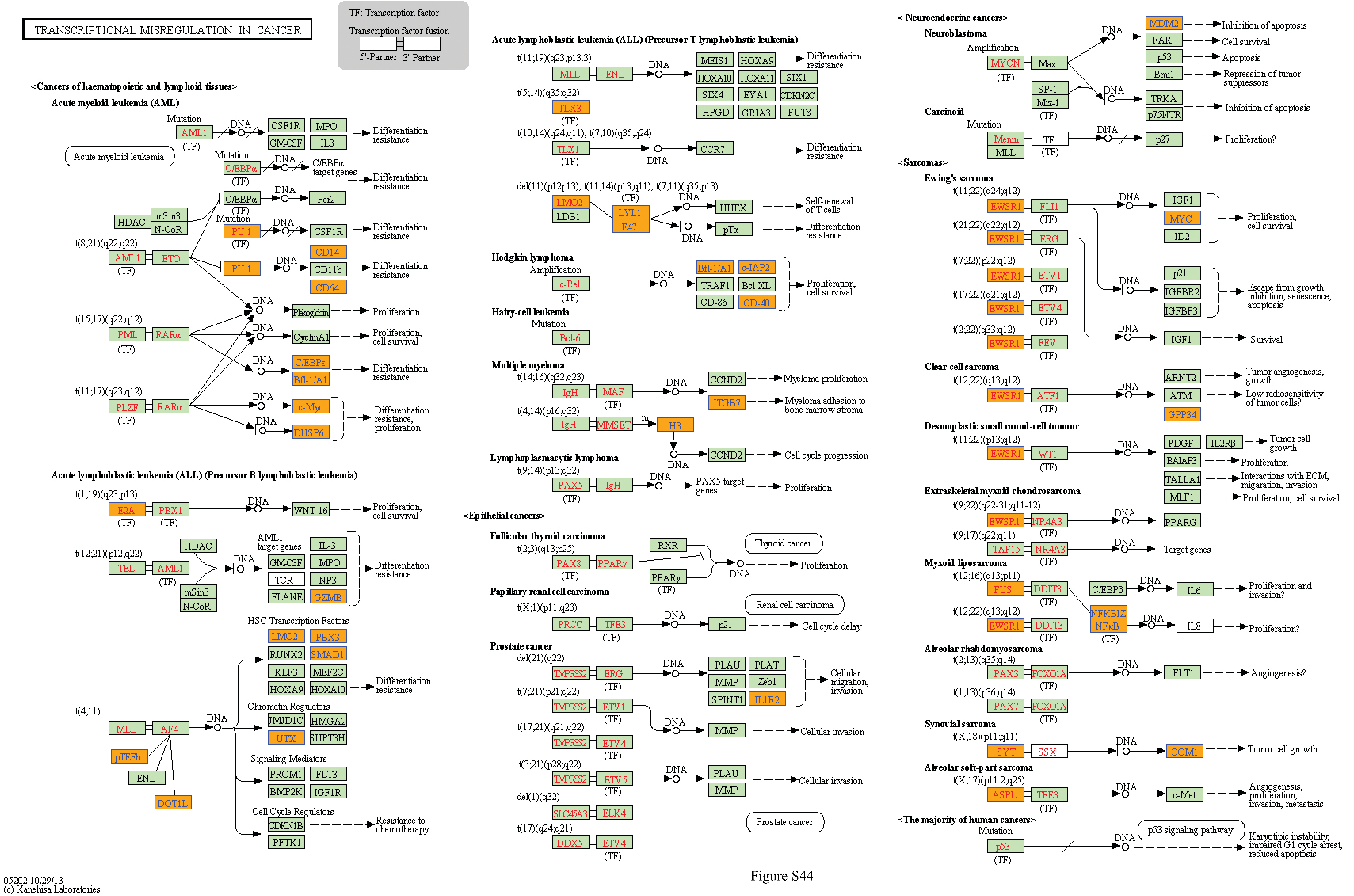
TRANSCRIPTIONAL MISREGULATION IN CANCER
TF: Transcription factor
Transcription factor fusion
5'-Partner 3'-Partner
<Cancers of haematopoietic and lymphoid tissues>
Acute myeloid leukemia (AML)
Mutation
AML1
(TF)
DNA
CSF1R
MPO
GM-CSF
IL3
Differentiation resistance
Acute myeloid leukemia
Mutation
C/EBPα
(TF)
DNA
C/EBPα target genes
Differentiation resistance
HDAC
mSin3
N-CoR
C/EBPα
(TF)
DNA
Per2
Mutation
PU.1
(TF)
DNA
CSF1R
Differentiation resistance
t(8;21)(q22;q22)
AML1
ETO
(TF)
PU.1
DNA
CD14
CD11b
CD64
Differentiation resistance
DNA
Plakoglobin
Proliferation
t(15;17)(q22;q12)
PML
RARα
(TF)
DNA
CyclinA1
Proliferation, cell survival
DNA
C/EBPε
Bfl-1/A1
Differentiation resistance
t(11;17)(q23;q12)
PLZF
RARα
(TF)
DNA
c-Myc
DNA
DUSP6
Differentiation resistance, proliferation
Acute lymphoblastic leukemia (ALL) (Precursor B lymphoblastic leukemia)
t(1;19)(q23;p13)
E2A
PBX1
(TF)
(TF)
DNA
WNT-16
Proliferation, cell survival
t(12;21)(p12;q22)
HDAC
TEL
AML1
(TF)
mSin3
N-CoR
DNA
AML1 target genes:
IL-3
GM-CSF
MPO
TCR
NP3
ELANE
GZMB
Differentiation resistance
HSC Transcription Factors
LMO2
PBX3
RUNX2
SMAD1
KLF3
MEF2C
HOXA9
HOXA10
Differentiation resistance
Chromatin Regulators
t(4;11)
MLL
AF4
DNA
JMJD1C
HMGA2
UTX
SUPT3H
pTEFb
ENL
DOT1L
Signaling Mediators
PROM1
FLT3
BMP2K
IGF1R
Cell Cycle Regulators
CDKN1B
PFTK1
Resistance to chemotherapy
Acute lymphoblastic leukemia (ALL) (Precursor T lymphoblastic leukemia)
t(11;19)(q23;p13.3)
MLL
ENL
DNA
MEIS1
HOXA9
HOXA10
HOXA11
SIX1
SIX4
EYA1
CDKN2C
HPGD
GRIA3
FUT8
Differentiation resistance
t(5;14)(q35;q32)
TLX3
(TF)
t(10;14)(q24;q11), t(7;10)(q35;q24)
TLX1
(TF)
DNA
CCR7
Differentiation resistance
del(11)(p12p13), t(11;14)(p13;q11), t(7;11)(q35;p13)
LMO2
LDB1
(TF)
LYL1
E47
DNA
HHEX
Self-renewal of T cells
DNA
pTα
Differentiation resistance
Hodgkin lymphoma
Amplification
c-Rel
(TF)
DNA
Bfl-1/A1
c-IAP2
TRAF1
Bcl-XL
CD-86
CD-40
Proliferation, cell survival
Hairy-cell leukemia
Mutation
Bcl-6
(TF)
Multiple myeloma
t(14;16)(q32;q23)
IgH
MAF
(TF)
DNA
CCND2
Myeloma proliferation
ITGB7
Myeloma adhesion to bone marrow stroma
t(4;14)(p16;q32)
IgH
MMSET
+m
H3
DNA
CCND2
Cell cycle progression
Lymphoplasmacytic lymphoma
t(9;14)(p13;q32)
PAX5
IgH
(TF)
DNA
PAX5 target genes
Proliferation
<Epithelial cancers>
Follicular thyroid carcinoma
t(2;3)(q13;p25)
PAX8
PPARγ
(TF)
RXR
PPARγ
(TF)
Thyroid cancer
DNA
Proliferation
Papillary renal cell carcinoma
t(X;1)(p11;q23)
PRCC
TFE3
(TF)
DNA
p21
Cell cycle delay
Renal cell carcinoma
Prostate cancer
del(21)(q22)
TMPRSS2
ERG
(TF)
DNA
PLAU
PLAT
MMP
Zeb1
SPINT1
IL1R2
Cellular migration, invasion
t(7;21)(p21;q22)
TMPRSS2
ETV1
(TF)
DNA
MMP
Cellular invasion
t(17;21)(q21;q22)
TMPRSS2
ETV4
(TF)
t(3;21)(p28;q22)
TMPRSS2
ETV5
(TF)
DNA
PLAU
MMP
Cellular invasion
del(1)(q32)
SLC45A3
ELK4
Prostate cancer
t(17)(q24;q21)
DDX5
ETV4
(TF)
< Neuroendocrine cancers>
Neuroblastoma
Amplification
MYCN
(TF)
Max
DNA
MDM2
Inhibition of apoptosis
FAK
Cell survival
p53
Apoptosis
Bmi1
Repression of tumor suppressors
SP-1
Miz-1
(TF)
DNA
TRKA
p75NTR
Inhibition of apoptosis
Carcinoid
Mutation
Menin
MLL
TF
(TF)
DNA
p27
Proliferation?
<Sarcomas>
Ewing's sarcoma
t(11;22)(q24;q12)
EWSR1
FLI1
(TF)
DNA
IGF1
MYC
ID2
Proliferation, cell survival
t(21;22)(q22;q12)
EWSR1
ERG
(TF)
t(7;22)(p22;q12)
EWSR1
ETV1
(TF)
DNA
p21
TGFBR2
IGFBP3
Escape from growth inhibition, senescence, apoptosis
t(17;22)(q21;q12)
EWSR1
ETV4
(TF)
t(2;22)(q33;q12)
EWSR1
FEV
(TF)
DNA
IGF1
Survival
Clear-cell sarcoma
t(12;22)(q13;q12)
EWSR1
ATF1
(TF)
DNA
ARNT2
Tumor angiogenesis, growth
ATM
Low radiosensitivity of tumor cells?
GPP34
Desmoplastic small round-cell tumour
t(11;22)(p13;q12)
EWSR1
WT1
(TF)
DNA
PDGF
IL2Rβ
Tumor cell growth
BAIAP3
Proliferation
TALLA1
Interactions with ECM, migration, invasion
MLF1
Proliferation, cell survival
Extraskeletal myxoid chondrosarcoma
t(9;22)(q22-31;q11-12)
EWSR1
NR4A3
(TF)
DNA
PPARG
t(9;17)(q22;q11)
TAF15
NR4A3
(TF)
DNA
Target genes
Myxoid liposarcoma
t(12;16)(q13;p11)
FUS
DDIT3
(TF)
C/EBPβ
DNA
IL6
Proliferation and invasion?
NFKBIZ
NFκB
(TF)
t(12;22)(q13;q12)
EWSR1
DDIT3
(TF)
DNA
IL8
Proliferation?
Alveolar rhabdomyosarcoma
t(2;13)(q35;q14)
PAX3
FOXO1A
(TF)
DNA
FLT1
Angiogenesis?
t(1;13)(p36;q14)
PAX7
FOXO1A
(TF)
Synovial sarcoma
t(X;18)(p11;q11)
SYT
SSX
DNA
COM1
Tumor cell growth
Alveolar soft-part sarcoma
t(X;17)(p11.2;q25)
ASPL
TFE3
(TF)
DNA
c-Met
Angiogenesis, proliferation, invasion, metastasis
<The majority of human cancers>
Mutation
p53
(TF)
DNA
p53 signaling pathway
Karyotipic instability, impaired G1 cycle arrest, reduced apoptosis
05202 10/29/13
(c) Kanehisa Laboratories

Figure S44

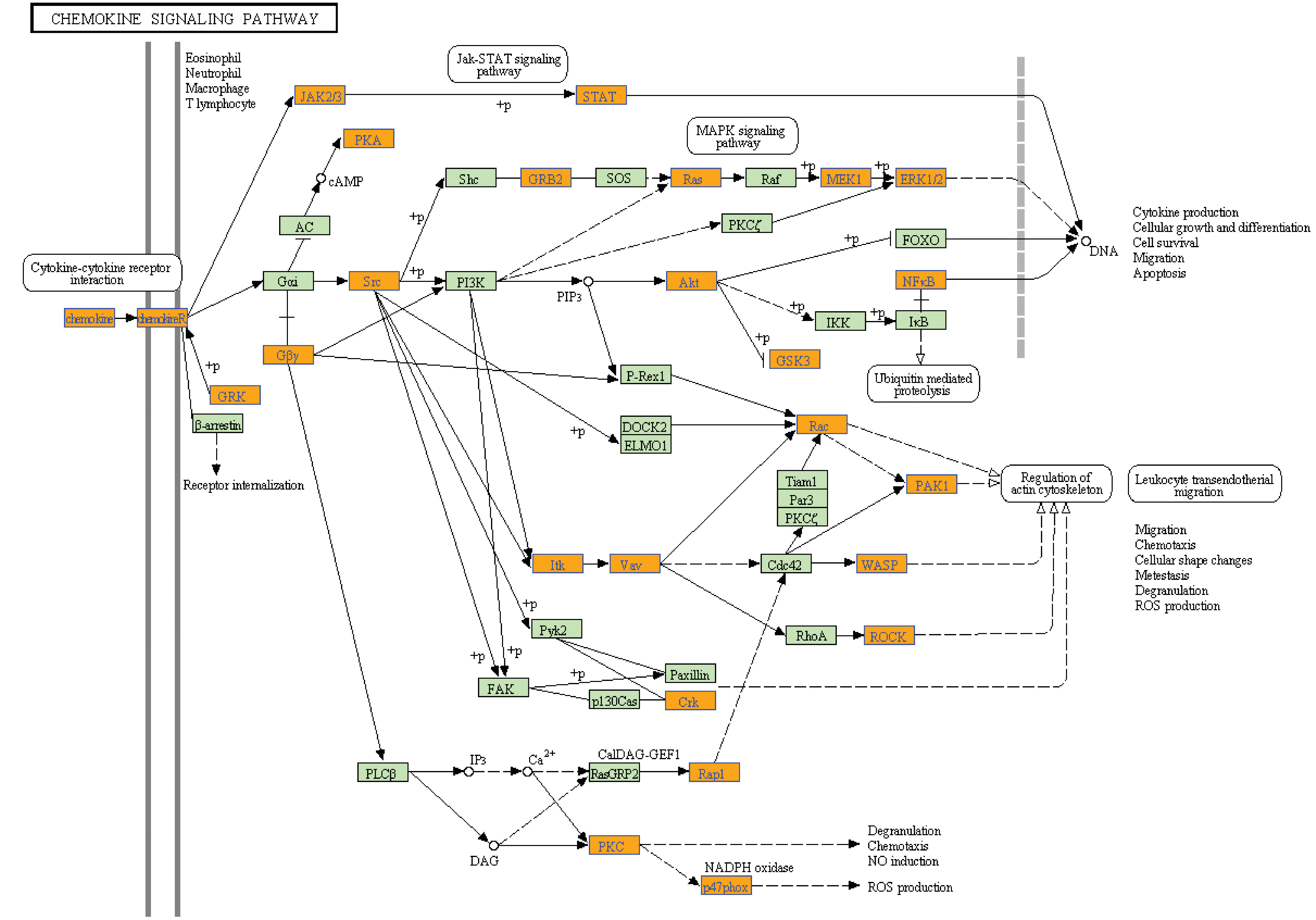
CHEMOKINE SIGNALING PATHWAY
Eosinophil
Neutrophil
Macrophage
T lymphocyte
Jak-STAT signaling pathway
JAK2/3
STAT
+p
PKA
cAMP
MAPK signaling pathway
Shc
GRB2
SOS
Ras
Raf
MEK1
ERK1/2
AC
PKCζ
FOXO
Cytokine production
Cellular growth and differentiation
Cell survival
Migration
Apoptosis
DNA
Cytokine-cytokine receptor interaction
Gαi
Src
PI3K
PIP3
Akt
NFκB
chemokine
chemokineR
IKK
IκB
GSK3
Gβγ
P-Rex1
Ubiquitin mediated proteolysis
GRK
β-arrestin
Receptor internalization
DOCK2
ELMO1
Rac
Tiam1
Par3
PKCζ
PAK1
Regulation of actin cytoskeleton
Leukocyte transendothelial migration
Migration
Chemotaxis
Cellular shape changes
Metestasis
Degranulation
ROS production
Itk
Vav
Cdc42
WASP
RhoA
ROCK
Pyk2
Paxillin
FAK
p130Cas
Crk
PLCβ
IP3
Ca2+
CalDAG-GEF1
RasGRP2
Rap1
DAG
PKC
Degranulation
Chemotaxis
NO induction
NADPH oxidase
p47phox
ROS production
04062 8/2/13
(c) Kanehisa Laboratories

Figure S55

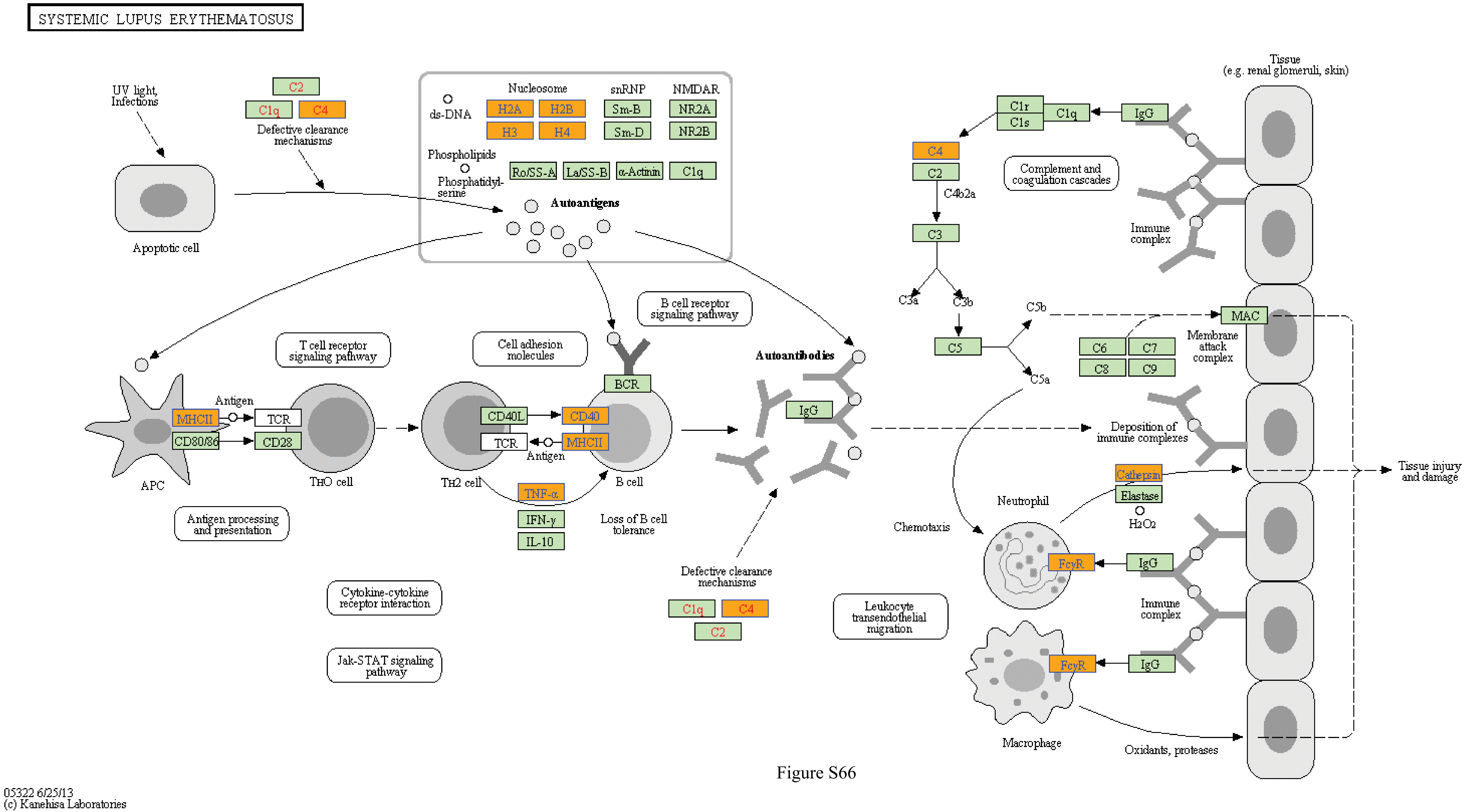
SYSTEMIC LUPUS ERYTHEMATOSUS
UV light, Infections
C2
C1q
C4
Defective clearance mechanisms
Apoptotic cell
ds-DNA
Nucleosome
H2A
H2B
H3
H4
snRNP
Sm-B
Sm-D
NMDAR
NR2A
NR2B
Phospholipids
Phosphatidyl-serine
Ro/SS-A
La/SS-B
α-Actinin
C1q
Autoantigens
B cell receptor signaling pathway
T cell receptor signaling pathway
Cell adhesion molecules
Antigen
MHCII
TCR
CD80/86
CD28
APC
TH0 cell
CD40L
CD40
TCR
MHCII
Antigen
TH2 cell
BCR
B cell
TNF-α
IFN-γ
IL-10
Loss of B cell tolerance
Antigen processing and presentation
Cytokine-cytokine receptor interaction
Jak-STAT signaling pathway
Autoantibodies
IgG
Defective clearance mechanisms
C1q
C4
C2
Leukocyte transendothelial migration
C1r
C1s
C1q
IgG
C4
C2
C4b2a
C3
C3a
C3b
C5
C5b
C5a
Complement and coagulation cascades
Immune complex
Tissue (e.g. renal glomeruli, skin)
MAC
C6
C7
C8
C9
Membrane attack complex
Deposition of immune complexes
Cathepsin
Elastase
H_2O_2
Tissue injury and damage
Chemotaxis
Neutrophil
FcγR
IgG
Immune complex
FcγR
IgG
Macrophage
Oxidants, proteases
05322 6/25/13
(c) Kanehisa Laboratories

Figure S66

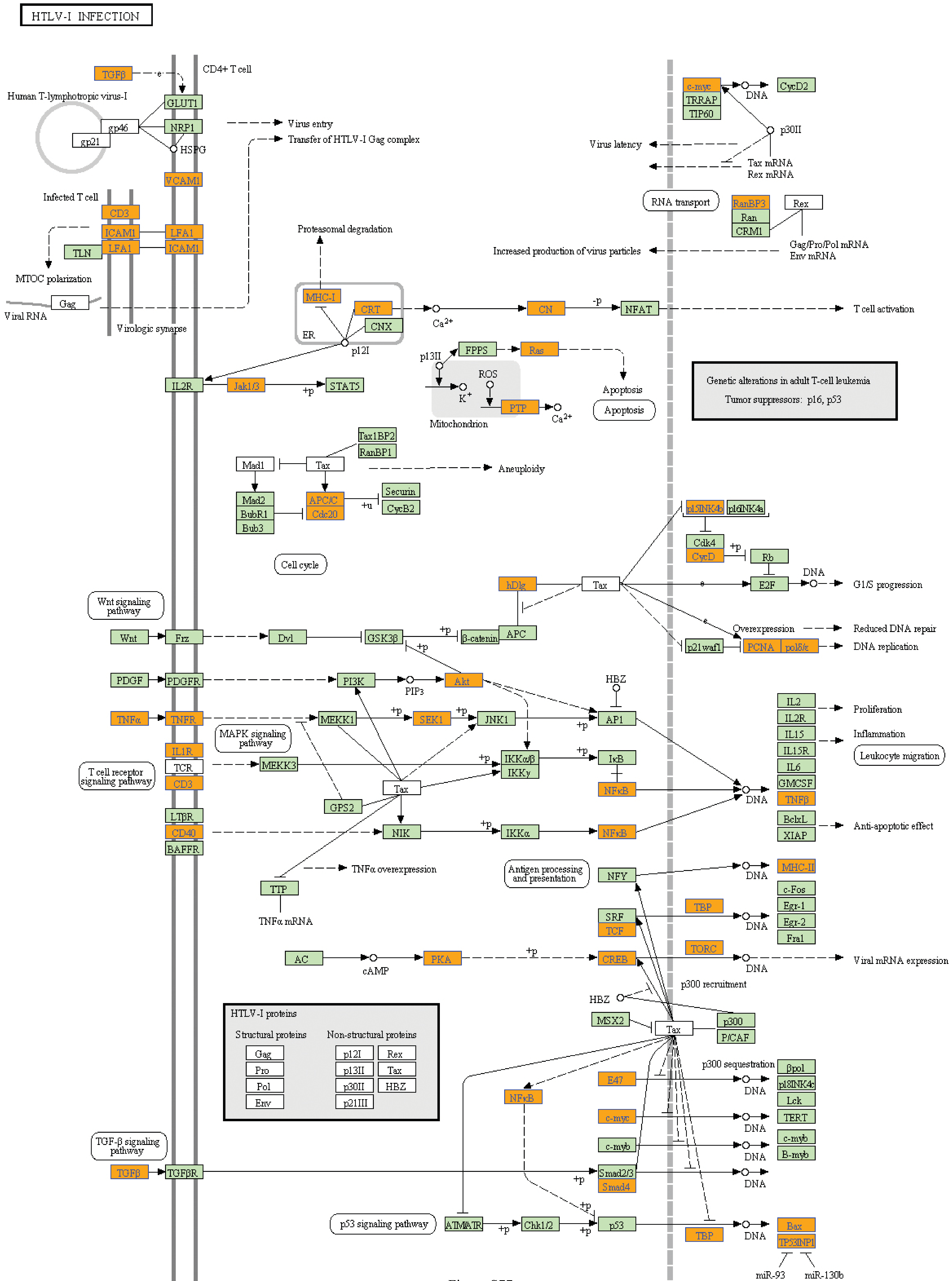

Figure S77

05166 9/2/13
(c) Kanehisa Laboratories

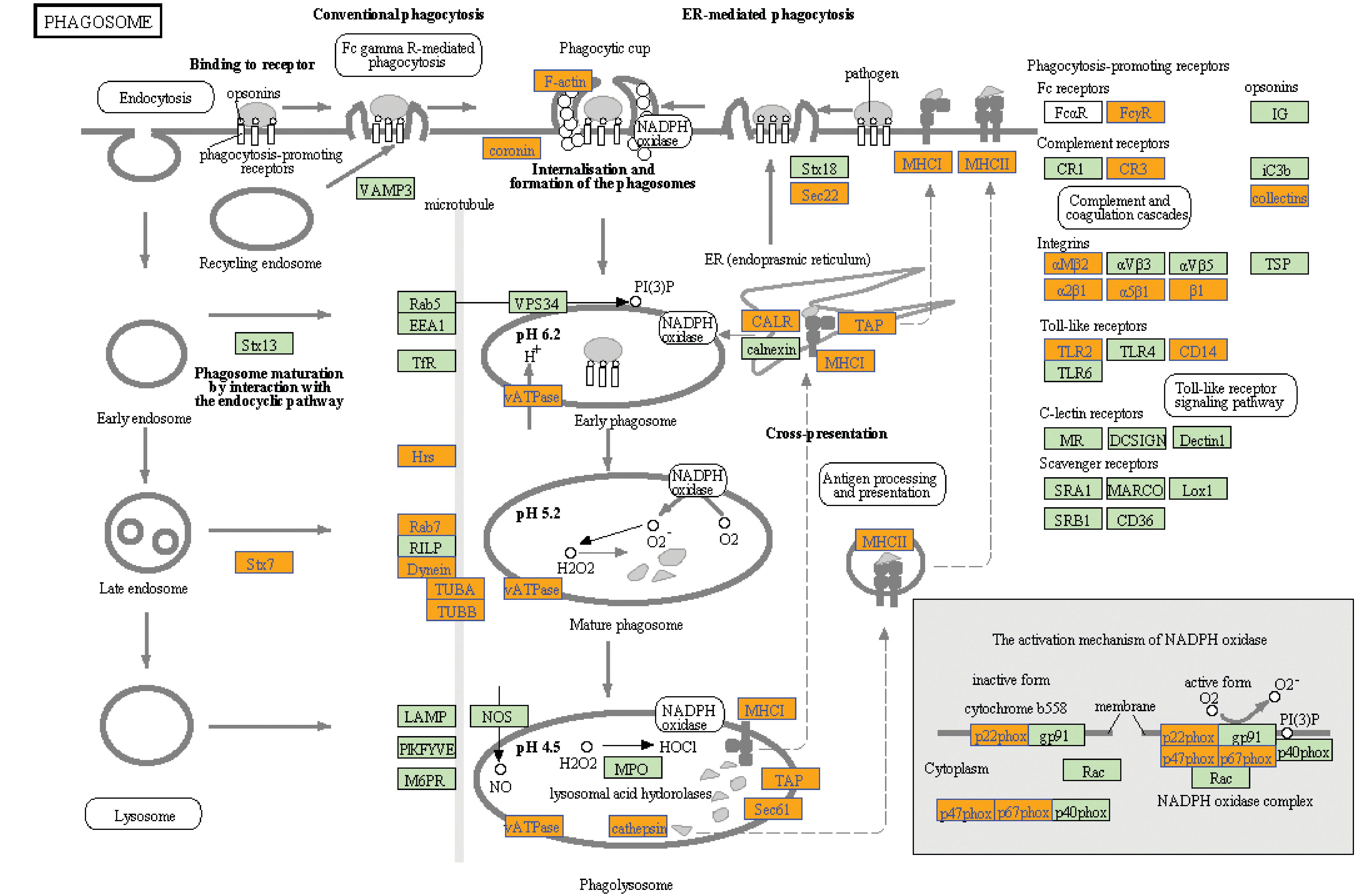

04145 12/27/13
(c) Kanehisa Laboratories

Figure S88

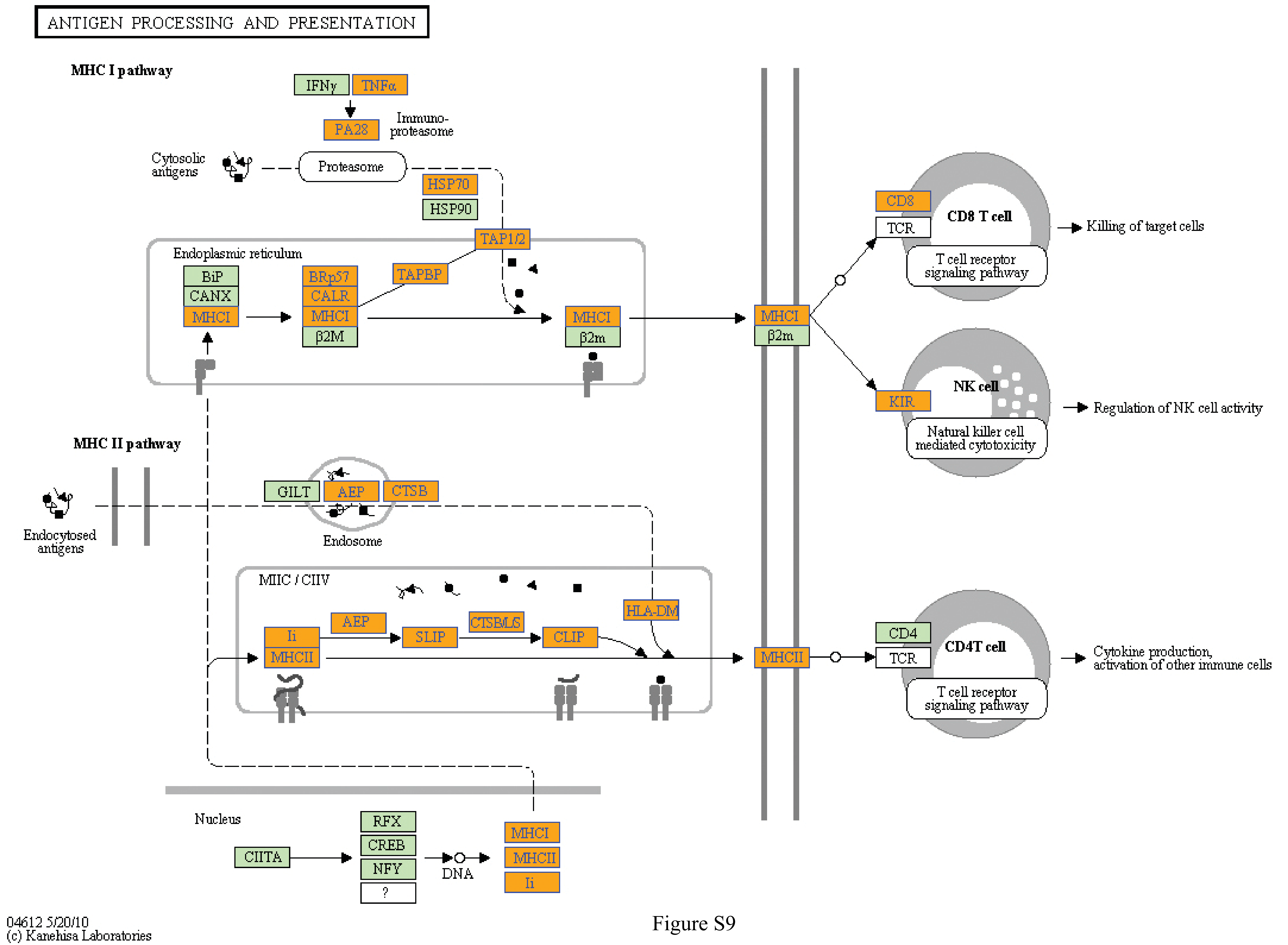
ANTIGEN PROCESSING AND PRESENTATION
MHC I pathway
IFNγ
TNFα
PA28
Immuno-
proteasome
Cytosolic
antigens
Proteasome
HSP70
HSP90
TAP1/2
Endoplasmic reticulum
BiP
CANX
MHCI
BRp57
CALR
MHCI
β2M
TAPBP
MHCI
β2m
MHCI
β2m
CD8
TCR
CD8 T cell
T cell receptor
signaling pathway
Killing of target cells
KIR
NK cell
Natural killer cell
mediated cytotoxicity
Regulation of NK cell activity
MHC II pathway
Endocytosed
antigens
GILT
AEP
CTSB
Endosome
MIIC / CIIV
Ii
MHCII
AEP
SLIP
CTSB/L/S
CLIP
HLA-DM
MHCII
CD4
TCR
CD4T cell
T cell receptor
signaling pathway
Cytokine production,
activation of other immune cells
Nucleus
CIITA
RFX
CREB
NFY
?
DNA
MHCI
MHCII
Ii
04612 5/20/10
(c) Kanehisa Laboratories

Figure S9

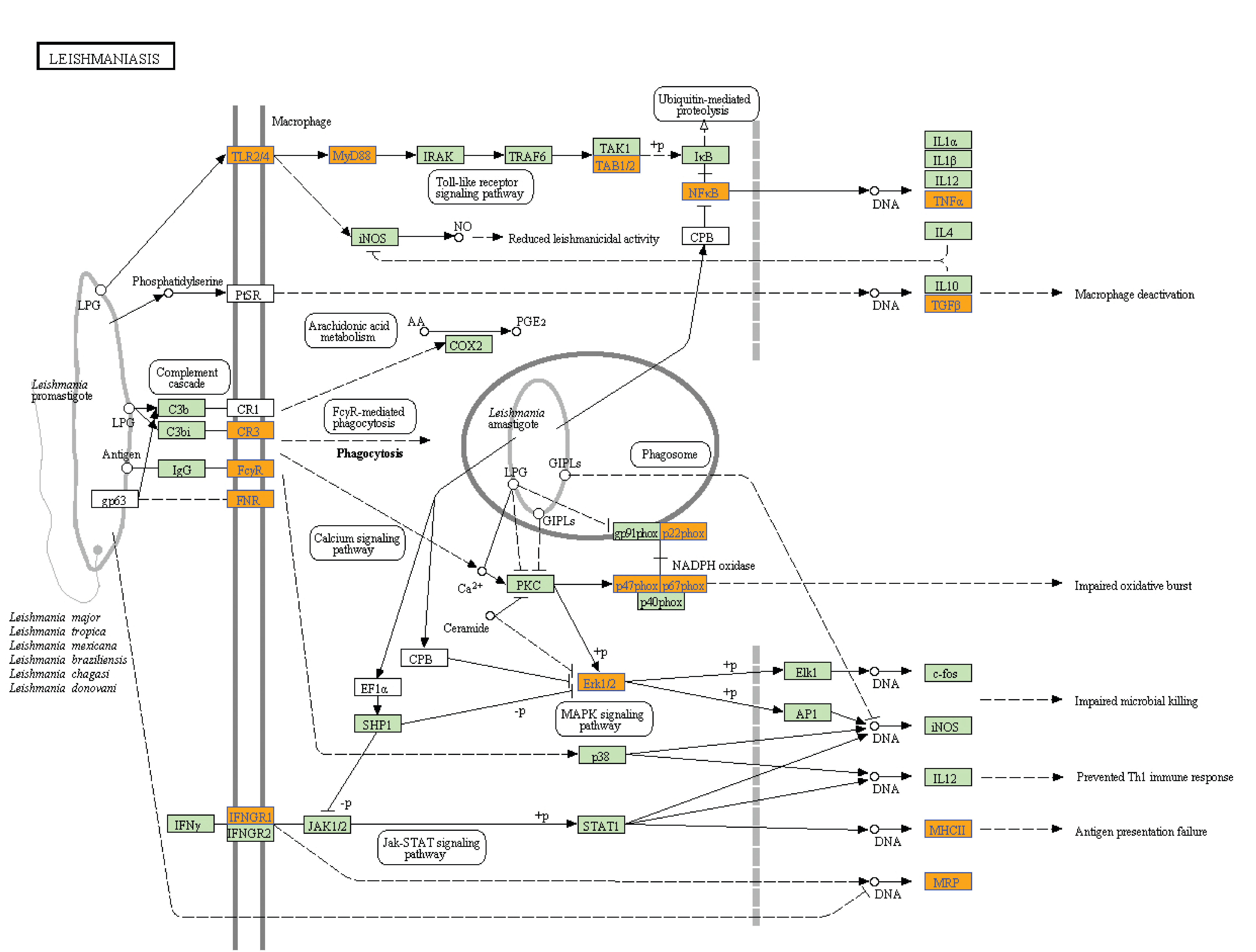

05140 5/29/13

(c) Kanehisa Laboratories

Figure S1010

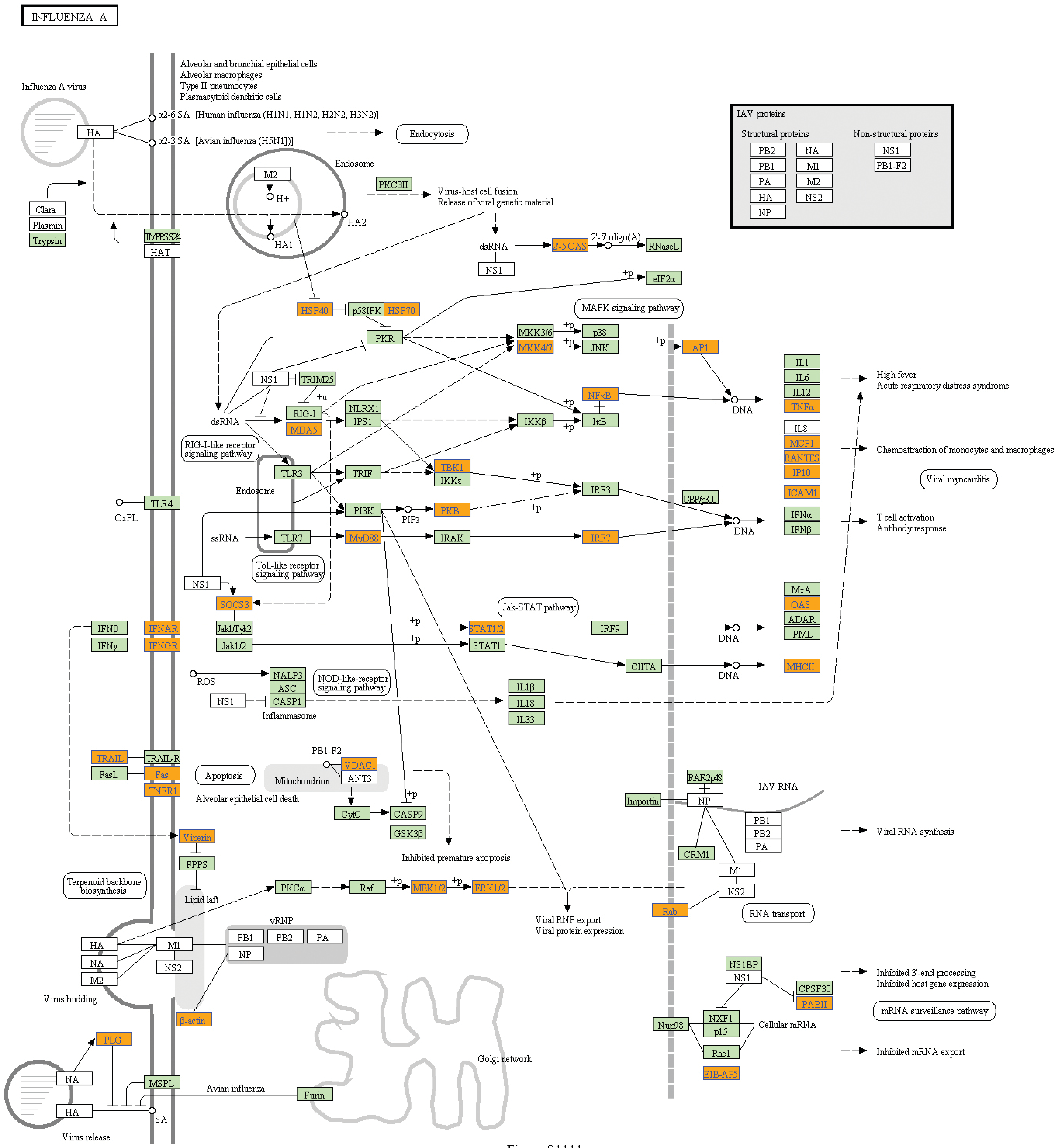

Figure S1111

05164 5/30/13
(c) Kanehisa Laboratories

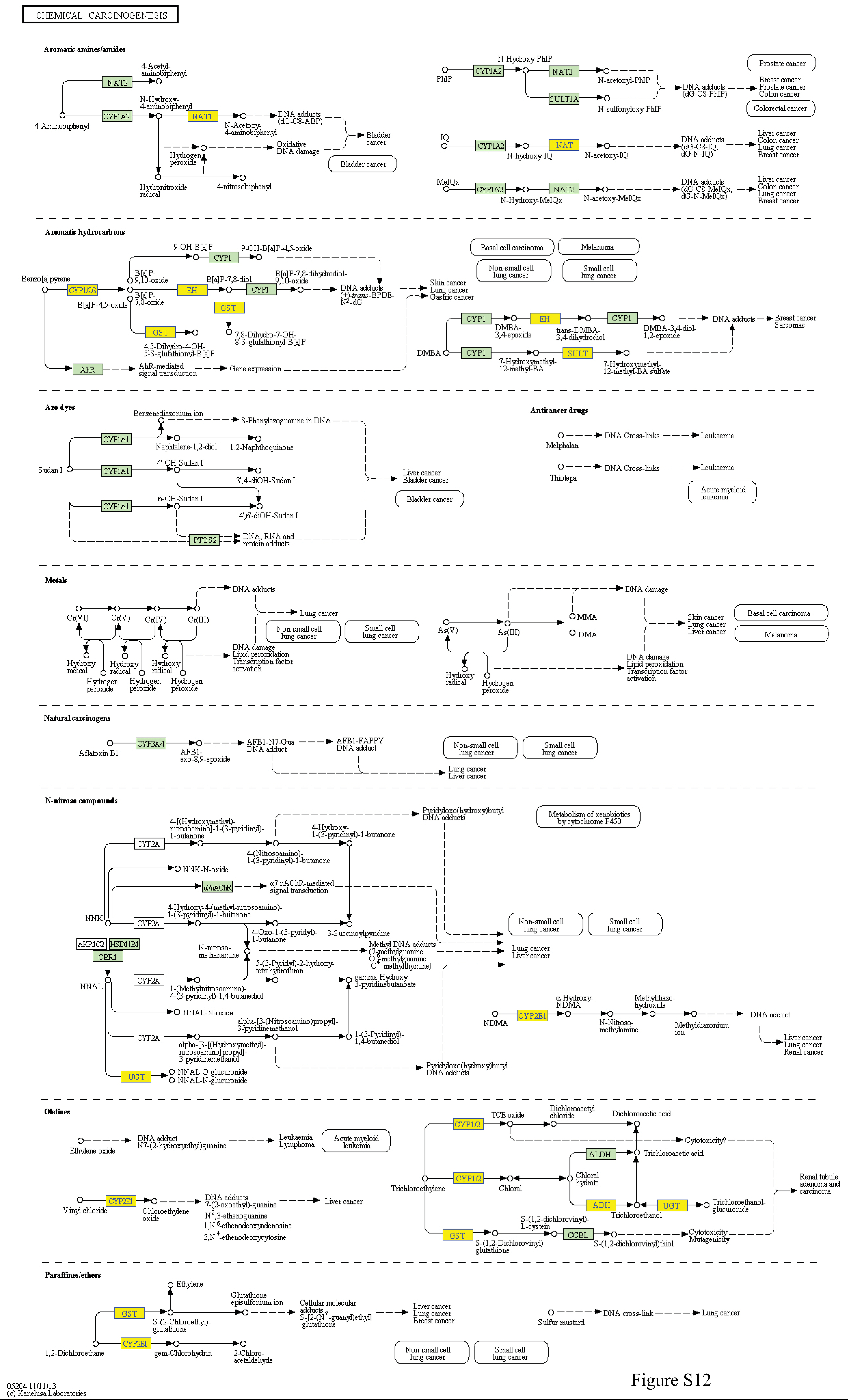
CHEMICAL CARCINOGENESIS
Aromatic amines/amides
NAT2
4-Acetyl-aminobiphenyl
CYP1A2
N-Hydroxy-4-aminobiphenyl
NAT1
N-Acetoxy-4-aminobiphenyl
DNA adducts (dG-C8-ABP)
4-Aminobiphenyl
Hydrogen peroxide
Oxidative DNA damage
Bladder cancer
Bladder cancer
Hydronitroxide radical
4-nitrosobiphenyl
N-Hydroxy-PhIP
PhIP
CYP1A2
NAT2
N-acetoxyl-PhIP
SULT1A
N-sulfonyloxy-PhIP
DNA adducts (dG-C8-PhIP)
Prostate cancer
Breast cancer
Prostate cancer
Colon cancer
Colorectal cancer
IQ
CYP1A2
N-hydroxy-IQ
NAT
N-acetoxy-IQ
DNA adducts (dG-C8-IQ, dG-N-IQ)
Liver cancer
Colon cancer
Lung cancer
Breast cancer
MeIQx
CYP1A2
N-Hydroxy-MeIQx
NAT2
N-acetoxy-MeIQx
DNA adducts (dG-C8-MeIQx, dG-N-MeIQx)
Liver cancer
Colon cancer
Lung cancer
Breast cancer
Aromatic hydrocarbons
9-OH-B[a]P
CYP1
9-OH-B[a]P-4,5-oxide
Benzo[a]pyrene
CYP1/2/3
B[a]P-9,10-oxide
B[a]P-7,8-oxide
B[a]P-4,5-oxide
EH
B[a]P-7,8-diol
CYP1
B[a]P-7,8-dihydrodiol-9,10-oxide
DNA adducts (+)-trans-BPDE-N²-dG
GST
7,8-Dihydro-7-OH-8-S-glutathionyl-B[a]P
GST
4,5-Dihydro-4-OH-5-S-glutathionyl-B[a]P
AhR
AhR-mediated signal transduction
Gene expression
Skin cancer
Lung cancer
Gastric cancer
Basal cell carcinoma
Melanoma
Non-small cell lung cancer
Small cell lung cancer
DMBA
CYP1
DMBA-3,4-epoxide
EH
trans-DMBA-3,4-dihydrodiol
CYP1
DMBA-3,4-diol-1,2-epoxide
DNA adducts
Breast cancer
Sarcomas
CYP1
7-Hydroxymethyl-12-methyl-BA
SULT
7-Hydroxymethyl-12-methyl-BA sulfate
Azo dyes
Benzenediazonium ion
8-Phenylazoguanine in DNA
CYP1A1
Naphtalene-1,2-diol
1,2-Naphthoquinone
Sudan I
CYP1A1
4'-OH-Sudan I
3',4'-diOH-Sudan I
CYP1A1
6-OH-Sudan I
4',6'-diOH-Sudan I
PTGS2
DNA, RNA and protein adducts
Liver cancer
Bladder cancer
Bladder cancer
Anticancer drugs
Melphalan
DNA Cross-links
Leukaemia
Thiotepa
DNA Cross-links
Leukaemia
Acute myeloid leukemia
Metals
Cr(VI)
Cr(V)
Cr(IV)
Cr(III)
DNA adducts
Lung cancer
Non-small cell lung cancer
Small cell lung cancer
Hydroxy radical
Hydroxy radical
Hydroxy radical
Hydrogen peroxide
Hydrogen peroxide
Hydrogen peroxide
DNA damage
Lipid peroxidation
Transcription factor activation
As(V)
As(III)
MMA
DMA
DNA damage
Skin cancer
Lung cancer
Liver cancer
Basal cell carcinoma
Melanoma
Hydroxy radical
Hydrogen peroxide
DNA damage
Lipid peroxidation
Transcription factor activation
Natural carcinogens
Aflatoxin B1
CYP3A4
AFB1-exo-8,9-epoxide
AFB1-N7-Gua DNA adduct
AFB1-FAPPY DNA adduct
Non-small cell lung cancer
Small cell lung cancer
Lung cancer
Liver cancer
N-nitroso compounds
4-[(Hydroxymethyl)-nitrosoamino]-1-(3-pyridinyl)-1-butanone
CYP2A
4-(Nitrosoamino)-1-(3-pyridinyl)-1-butanone
4-Hydroxy-1-(3-pyridinyl)-1-butanone
Pyridyloxo(hydroxy)butyl DNA adducts
Metabolism of xenobiotics by cytochrome P450
NNK-N-oxide
α7nAChR
α7 nAChR-mediated signal transduction
NNK
CYP2A
4-Hydroxy-4-(methyl-nitrosoamino)-1-(3-pyridinyl)-1-butanone
4-Oxo-1-(3-pyridyl)-1-butanone
3-Succinoylpyridine
N-nitroso-methanamine
Methyl DNA adducts
(7-methylguanine
O⁶-methylguanine
O⁴-methylthymine)
Non-small cell lung cancer
Small cell lung cancer
Lung cancer
Liver cancer
AKR1C2
HSD11B1
CBR1
NNAL
CYP2A
1-(Methylnitrosoamino)-4-(3-pyridinyl)-1,4-butanediol
5-(3-Pyridyl)-2-hydroxy-tetrahydrofuran
gamma-Hydroxy-3-pyridinebutanoate
NNAL-N-oxide
alpha-[3-(Nitrosoamino)propyl]-3-pyridinemethanol
CYP2A
alpha-[3-[(Hydroxymethyl)-nitrosoamino]propyl]-3-pyridinemethanol
1-(3-Pyridinyl)-1,4-butanediol
Pyridyloxo(hydroxy)butyl DNA adducts
UGT
NNAL-O-glucuronide
NNAL-N-glucuronide
NDMA
CYP2E1
α-Hydroxy-NDMA
N-Nitroso-methylamine
Methyldiazo-hydroxide
Methyldiazonium ion
DNA adduct
Liver cancer
Lung cancer
Renal cancer
Olefines
Ethylene oxide
DNA adduct N7-(2-hydroxyethyl)guanine
Leukaemia
Lymphoma
Acute myeloid leukemia
Vinyl chloride
CYP2E1
Chloroethylene oxide
DNA adducts
7-(2-oxoethyl)-guanine
N²,3-ethenoguanine
1,N⁶-ethenodeoxyadenosine
3,N⁴-ethenodeoxycytosine
Liver cancer
Trichloroethylene
CYP1/2
TCE oxide
Dichloroacetyl chloride
Dichloroacetic acid
Cytotoxicity?
ALDH
Trichloroacetic acid
CYP1/2
Chloral
Chloral hydrate
ADH
Trichloroethanol
UGT
Trichloroethanol-glucuronide
Renal tubule adenoma and carcinoma
GST
S-(1,2-Dichlorovinyl) glutathione
S-(1,2-dichlorovinyl)-L-cystein
CCBL
S-(1,2-dichlorovinyl)thiol
Cytotoxicity
Mutagenicity
Paraffines/ethers
Ethylene
GST
S-(2-Chloroethyl)-glutathione
Glutathione episulfonium ion
Cellular molecular adducts
S-[2-(N⁷-guanyl)ethyl] glutathione
Liver cancer
Lung cancer
Breast cancer
1,2-Dichloroethane
CYP2E1
gem-Chlorohydrin
2-Chloro-acetaldehyde
Non-small cell lung cancer
Small cell lung cancer
Sulfur mustard
DNA cross-link
Lung cancer
05204 11/11/13
(c) Kanehisa Laboratories

Figure S12

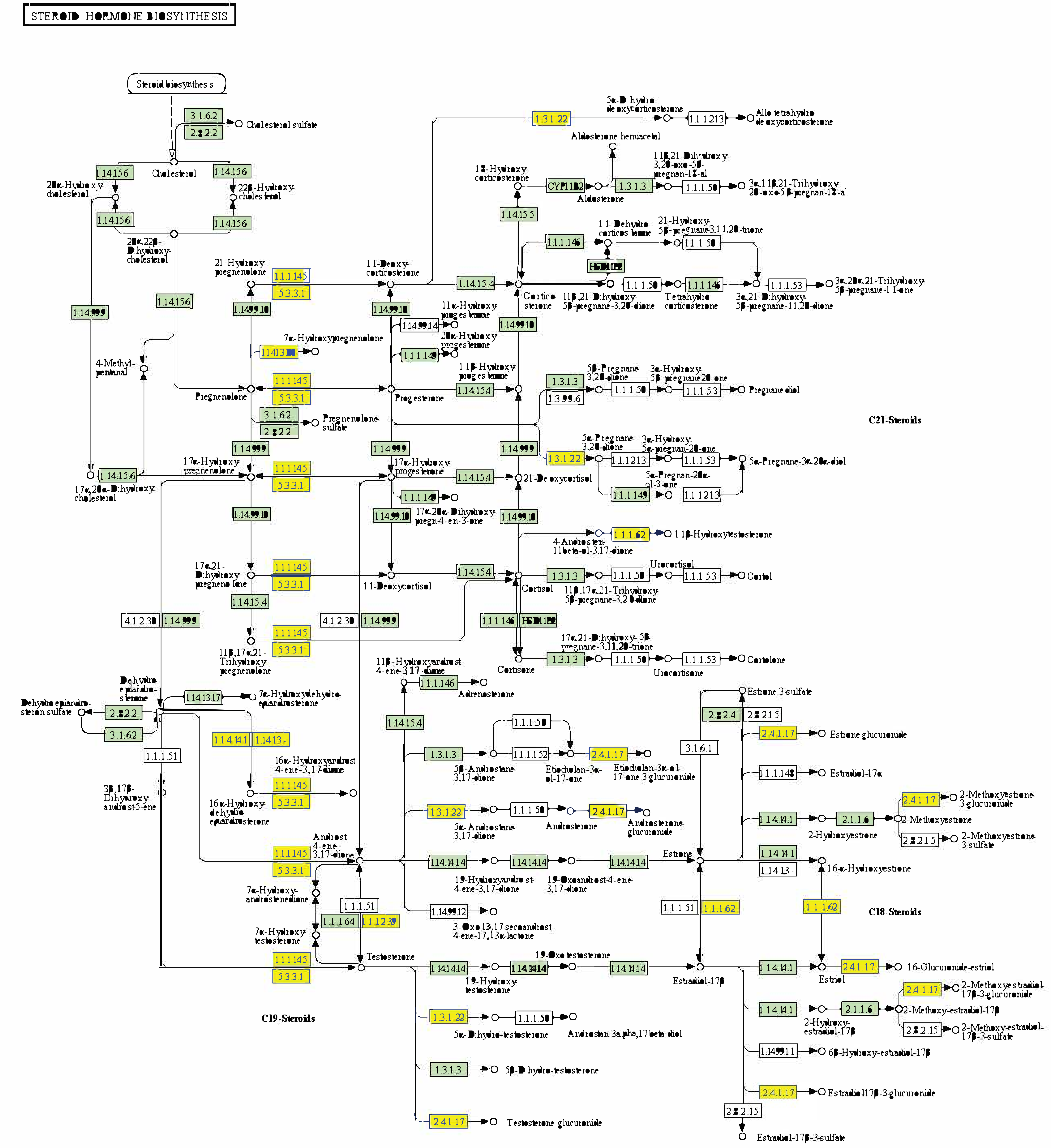
STEROID HORMONE BIOSYNTHESIS
Steroid biosynthesis
3.1.6.2
2.8.2.2
Cholesterol sulfate
Cholesterol
1.14.15.6
20α-Hydroxycholesterol
22β-Hydroxycholesterol
20α,22β-Dihydroxycholesterol
1.14.99
4-Methylpentanal
17α,20α-Dihydroxycholesterol
21-Hydroxypregnenolone
1.1.1.145
5.3.3.1
1.14.99.10
7α-Hydroxypregnenolone
1.14.13.100
Pregnenolone
Pregnenolone sulfate
17α-Hydroxypregnenolone
17α,21-Dihydroxypregnenolone
11β,17α,21-Trihydroxypregnenolone
4.1.2.30
1.14.99.9
11-Deoxycorticosterone
1.14.15.4
1.14.99.14
11α-Hydroxyprogesterone
20α-Hydroxyprogesterone
1.1.1.149
11β-Hydroxyprogesterone
Progesterone
1.14.15.4
17α-Hydroxyprogesterone
17α,20α-Dihydroxypregn-4-en-3-one
11-Deoxycortisol
1.3.1.22
5α-Dihydrodeoxycorticosterone
1.1.1.213
Allotetrahydrodeoxycorticosterone
Aldosterone hemiacetal
18-Hydroxycorticosterone
CYP11B2
1.3.1.3
Aldosterone
11β,21-Dihydroxy-3,20-oxo-5β-pregnan-18-al
1.1.1.50
3α,11β,21-Trihydroxy-20-oxo-5β-pregnan-18-al
1.14.15.5
1.1.1.146
11-Dehydrocorticosterone
HSD11B2
21-Hydroxy-5β-pregnane-3,11,20-trione
Corticosterone
11β,21-Dihydroxy-5β-pregnane-3,20-dione
Tetrahydrocorticosterone
3α,21-Dihydroxy-5β-pregnane-11,20-dione
1.1.1.53
3α,20α,21-Trihydroxy-5β-pregnan-11-one
5β-Pregnane-3,20-dione
3α-Hydroxy-5β-pregnan-20-one
Pregnanediol
1.3.99.6
C21-Steroids
5α-Pregnane-3,20-dione
3α-Hydroxy-5α-pregnan-20-one
5α-Pregnane-3α,20α-diol
5α-Pregnan-20α-ol-3-one
21-Deoxycortisol
1.1.1.62
4-Androsten-11beta-ol-3,17-dione
11β-Hydroxytestosterone
Urocortisol
Cortol
Cortisol
11β,17α,21-Trihydroxy-5β-pregnane-3,20-dione
17α,21-Dihydroxy-5β-pregnane-3,11,20-trione
Cortisone
Urocortisone
Cortolone
11β-Hydroxyandrost-4-ene-3,17-dione
Adrenosterone
1.14.15.4
Dehydroepiandrosterone
Dehydroepiandrosterone sulfate
1.14.13.17
7α-Hydroxydehydroepiandrosterone
1.14.14.1
1.14.13.-
1.1.1.51
16α-Hydroxyandrost-4-ene-3,17-dione
16α-Hydroxydehydroepiandrosterone
3β,17β-Dihydroxyandrost-5-ene
1.1.1.50
1.1.1.52
2.4.1.17
5β-Androstane-3,17-dione
Etiocholan-3α-ol-17-one
Etiocholan-3α-ol-17-one 3-glucuronide
5α-Androstane-3,17-dione
Androsterone
Androsterone glucuronide
Androst-4-ene-3,17-dione
1.14.14.14
19-Hydroxyandrost-4-ene-3,17-dione
19-Oxoandrost-4-ene-3,17-dione
Estrone
7α-Hydroxyandrostenedione
1.1.1.64
1.1.1.239
1.14.99.12
3-Oxo-13,17-secoandrost-4-ene-17,13α-lactone
7α-Hydroxytestosterone
Testosterone
19-Oxotestosterone
19-Hydroxytestosterone
Estradiol-17β
C19-Steroids
5α-Dihydrotestosterone
Androstan-3alpha,17beta-diol
5β-Dihydrotestosterone
Testosterone glucuronide
Estrone 3-sulfate
2.8.2.4
2.8.2.15
3.1.6.1
Estrone glucuronide
1.1.1.148
Estradiol-17α
2.1.1.6
2-Hydroxyestrone
2-Methoxyestrone
2-Methoxyestrone 3-glucuronide
2-Methoxyestrone 3-sulfate
16α-Hydroxyestrone
C18-Steroids
Estriol
16-Glucuronide-estriol
2-Hydroxyestradiol-17β
2-Methoxy-estradiol-17β
2-Methoxyestradiol-17β-3-glucuronide
2-Methoxy-estradiol-17β-3-sulfate
1.14.99.11
6β-Hydroxy-estradiol-17β
Estradiol-17β-3-glucuronide
Estradiol-17β-3-sulfate
00140 12/18/13
(c) Kanehisa Laboratories

Figure S13

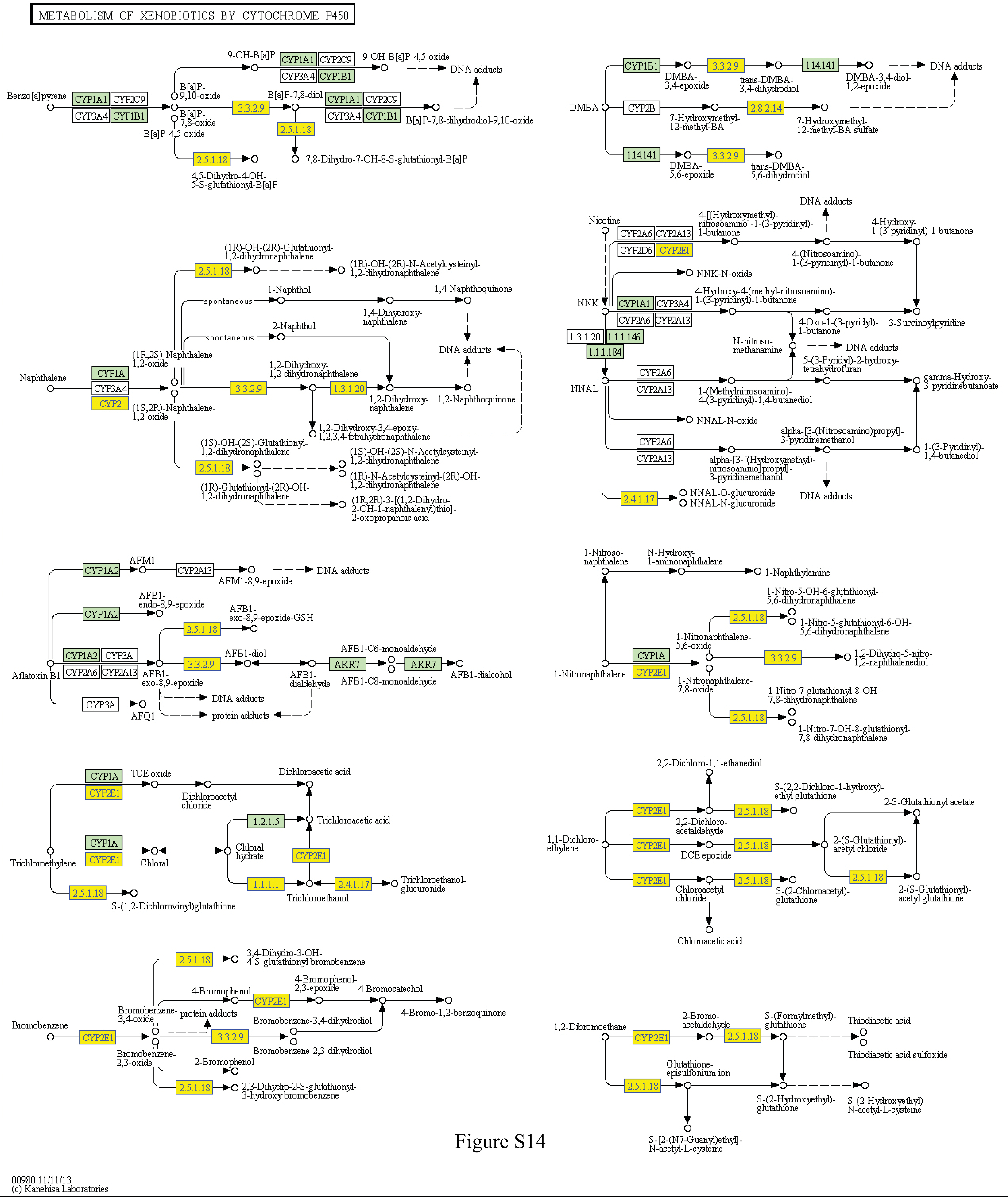

METABOLISM OF XENOBIOTICS BY CYTOCHROME P450
Benzo[a]pyrene
CYP1A1
CYP2C9
CYP3A4
CYP1B1
9-OH-B[a]P
9-OH-B[a]P-4,5-oxide
DNA adducts
B[a]P-9,10-oxide
B[a]P-7,8-oxide
B[a]P-4,5-oxide
3.3.2.9
B[a]P-7,8-diol
2.5.1.18
B[a]P-7,8-dihydrodiol-9,10-oxide
7,8-Dihydro-7-OH-8-S-glutathionyl-B[a]P
4,5-Dihydro-4-OH-5-S-glutathionyl-B[a]P
DMBA
CYP1B1
DMBA-3,4-epoxide
trans-DMBA-3,4-dihydrodiol
1.14.14.1
DMBA-3,4-diol-1,2-epoxide
CYP2B
7-Hydroxymethyl-12-methyl-BA
2.8.2.14
7-Hydroxymethyl-12-methyl-BA sulfate
DMBA-5,6-epoxide
trans-DMBA-5,6-dihydrodiol
Nicotine
NNK
CYP2A6
CYP2A13
CYP2D6
CYP2E1
CYP3A4
4-[(Hydroxymethyl)-nitrosoamino]-1-(3-pyridinyl)-1-butanone
4-(Nitrosoamino)-1-(3-pyridinyl)-1-butanone
4-Hydroxy-1-(3-pyridinyl)-1-butanone
NNK-N-oxide
4-Hydroxy-4-(methyl-nitrosoamino)-1-(3-pyridinyl)-1-butanone
4-Oxo-1-(3-pyridyl)-1-butanone
3-Succinoylpyridine
1.3.1.20
1.1.1.146
1.1.1.184
N-nitroso-methanamine
5-(3-Pyridyl)-2-hydroxy-tetrahydrofuran
NNAL
1-(Methylnitrosoamino)-4-(3-pyridinyl)-1,4-butanediol
gamma-Hydroxy-3-pyridinebutanoate
NNAL-N-oxide
alpha-[3-(Nitrosoamino)propyl]-3-pyridinemethanol
alpha-[3-[(Hydroxymethyl)-nitrosoamino]propyl]-3-pyridinemethanol
1-(3-Pyridinyl)-1,4-butanediol
2.4.1.17
NNAL-O-glucuronide
NNAL-N-glucuronide
Naphthalene
CYP1A
CYP2
(1R,2S)-Naphthalene-1,2-oxide
(1S,2R)-Naphthalene-1,2-oxide
(1R)-OH-(2R)-Glutathionyl-1,2-dihydronaphthalene
(1R)-OH-(2R)-N-Acetylcysteinyl-1,2-dihydronaphthalene
spontaneous
1-Naphthol
1,4-Dihydroxy-naphthalene
1,4-Naphthoquinone
2-Naphthol
1,2-Dihydroxy-1,2-dihydronaphthalene
1.3.1.20
1,2-Dihydroxy-naphthalene
1,2-Naphthoquinone
1,2-Dihydroxy-3,4-epoxy-1,2,3,4-tetrahydronaphthalene
(1S)-OH-(2S)-Glutathionyl-1,2-dihydronaphthalene
(1S)-OH-(2S)-N-Acetylcysteinyl-1,2-dihydronaphthalene
(1R)-N-Acetylcysteinyl-(2R)-OH-1,2-dihydronaphthalene
(1R)-Glutathionyl-(2R)-OH-1,2-dihydronaphthalene
(1R,2R)-3-[(1,2-Dihydro-2-OH-1-naphthalenyl)thio]-2-oxopropanoic acid
Aflatoxin B1
CYP1A2
AFM1
AFM1-8,9-epoxide
AFB1-endo-8,9-epoxide
AFB1-exo-8,9-epoxide-GSH
CYP3A
AFB1-exo-8,9-epoxide
AFB1-diol
AFB1-dialdehyde
AKR7
AFB1-C6-monoaldehyde
AFB1-C8-monoaldehyde
AFB1-dialcohol
protein adducts
AFQ1
1-Nitronaphthalene
1-Nitroso-naphthalene
N-Hydroxy-1-aminonaphthalene
1-Naphthylamine
1-Nitronaphthalene-5,6-oxide
1-Nitro-5-OH-6-glutathionyl-5,6-dihydronaphthalene
1-Nitro-5-glutathionyl-6-OH-5,6-dihydronaphthalene
1,2-Dihydro-5-nitro-1,2-naphthalenediol
1-Nitronaphthalene-7,8-oxide
1-Nitro-7-glutathionyl-8-OH-7,8-dihydronaphthalene
1-Nitro-7-OH-8-glutathionyl-7,8-dihydronaphthalene
Trichloroethylene
TCE oxide
Dichloroacetyl chloride
Dichloroacetic acid
1.2.1.5
Trichloroacetic acid
Chloral
Chloral hydrate
1.1.1.1
Trichloroethanol
Trichloroethanol-glucuronide
S-(1,2-Dichlorovinyl)glutathione
1,1-Dichloro-ethylene
2,2-Dichloro-1,1-ethanediol
2,2-Dichloro-acetaldehyde
S-(2,2-Dichloro-1-hydroxy)-ethyl glutathione
2-S-Glutathionyl acetate
DCE epoxide
2-(S-Glutathionyl)-acetyl chloride
Chloroacetyl chloride
S-(2-Chloroacetyl)-glutathione
2-(S-Glutathionyl)-acetyl glutathione
Chloroacetic acid
Bromobenzene
Bromobenzene-3,4-oxide
Bromobenzene-2,3-oxide
3,4-Dihydro-3-OH-4-S-glutathionyl bromobenzene
4-Bromophenol
4-Bromophenol-2,3-epoxide
4-Bromocatechol
4-Bromo-1,2-benzoquinone
Bromobenzene-3,4-dihydrodiol
Bromobenzene-2,3-dihydrodiol
2-Bromophenol
2,3-Dihydro-2-S-glutathionyl-3-hydroxy bromobenzene
1,2-Dibromoethane
2-Bromo-acetaldehyde
S-(Formylmethyl)-glutathione
Thiodiacetic acid
Thiodiacetic acid sulfoxide
Glutathione-episulfonium ion
S-(2-Hydroxyethyl)-glutathione
S-(2-Hydroxyethyl)-N-acetyl-L-cysteine
S-[2-(N7-Guanyl)ethyl]-N-acetyl-L-cysteine
00980 11/11/13
(c) Kanehisa Laboratories

Figure S14

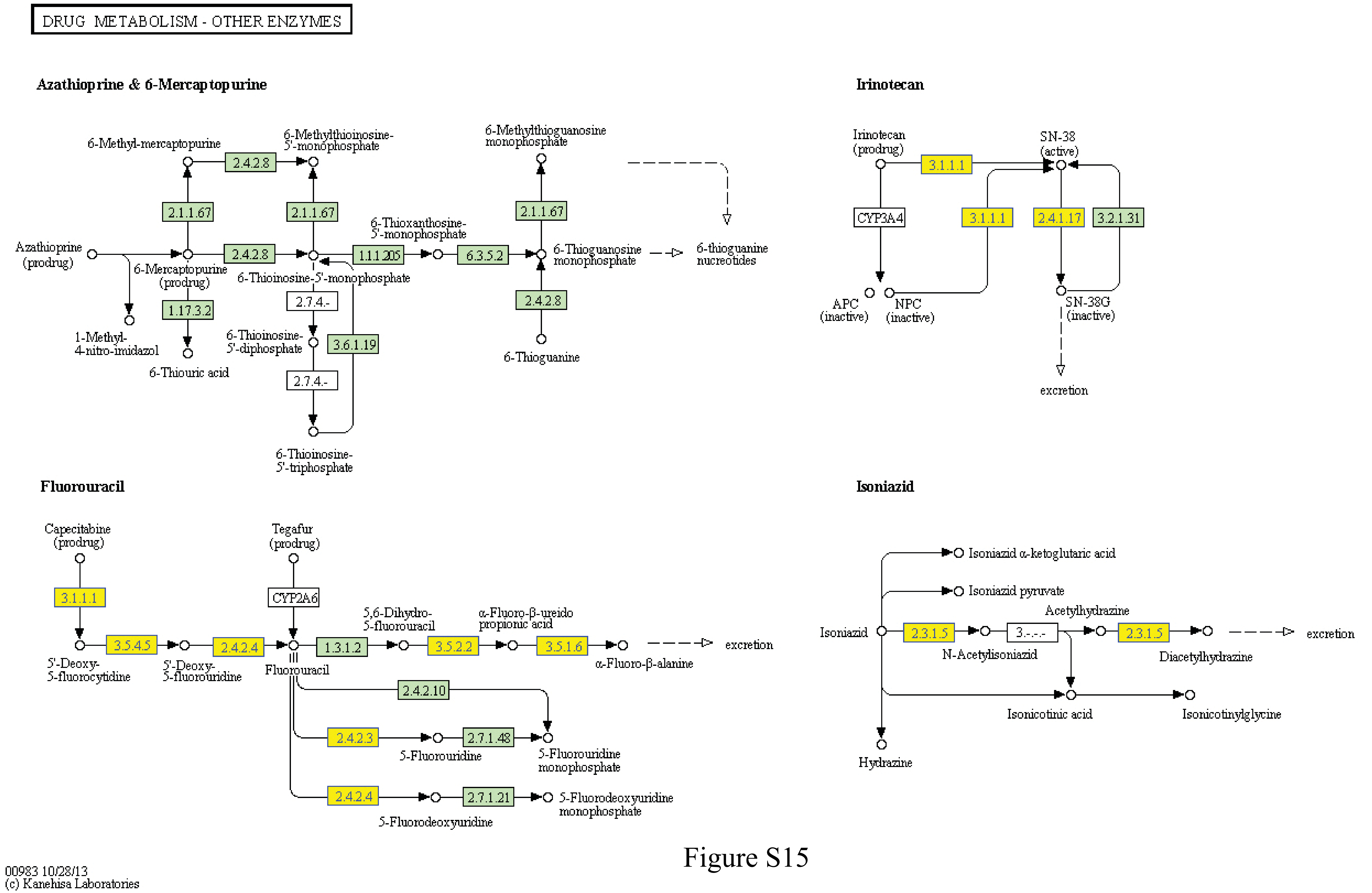
DRUG METABOLISM - OTHER ENZYMES
Azathioprine & 6-Mercaptopurine
6-Methyl-mercaptopurine
2.4.2.8
6-Methylthioinosine-5'-monophosphate
6-Methylthioguanosine monophosphate
2.1.1.67
2.1.1.67
2.1.1.67
6-Thioxanthosine-5'-monophosphate
Azathioprine (prodrug)
2.4.2.8
1.1.1.205
6.3.5.2
6-Thioguanosine monophosphate
6-thioguanine nucreotides
6-Mercaptopurine (prodrug)
6-Thioinosine-5'-monophosphate
1.17.3.2
2.7.4.-
2.4.2.8
1-Methyl-4-nitro-imidazol
6-Thioinosine-5'-diphosphate
3.6.1.19
6-Thiouric acid
2.7.4.-
6-Thioguanine
6-Thioinosine-5'-triphosphate
Irinotecan
Irinotecan (prodrug)
SN-38 (active)
3.1.1.1
CYP3A4
3.1.1.1
2.4.1.17
3.2.1.31
APC (inactive)
NPC (inactive)
SN-38G (inactive)
excretion
Fluorouracil
Capecitabine (prodrug)
Tegafur (prodrug)
3.1.1.1
CYP2A6
5,6-Dihydro-5-fluorouracil
α-Fluoro-β-ureido propionic acid
3.5.4.5
2.4.2.4
1.3.1.2
3.5.2.2
3.5.1.6
excretion
5'-Deoxy-5-fluorocytidine
5'-Deoxy-5-fluorouridine
Fluorouracil
α-Fluoro-β-alanine
2.4.2.10
2.4.2.3
2.7.1.48
5-Fluorouridine
5-Fluorouridine monophosphate
2.4.2.4
2.7.1.21
5-Fluorodeoxyuridine monophosphate
5-Fluorodeoxyuridine
Isoniazid
Isoniazid α-ketoglutaric acid
Isoniazid pyruvate
Acetylhydrazine
Isoniazid
2.3.1.5
3.-.-.-
2.3.1.5
excretion
N-Acetylisoniazid
Diacetylhydrazine
Isonicotinic acid
Isonicotinylglycine
Hydrazine
00983 10/28/13
(c) Kanehisa Laboratories

Figure S15

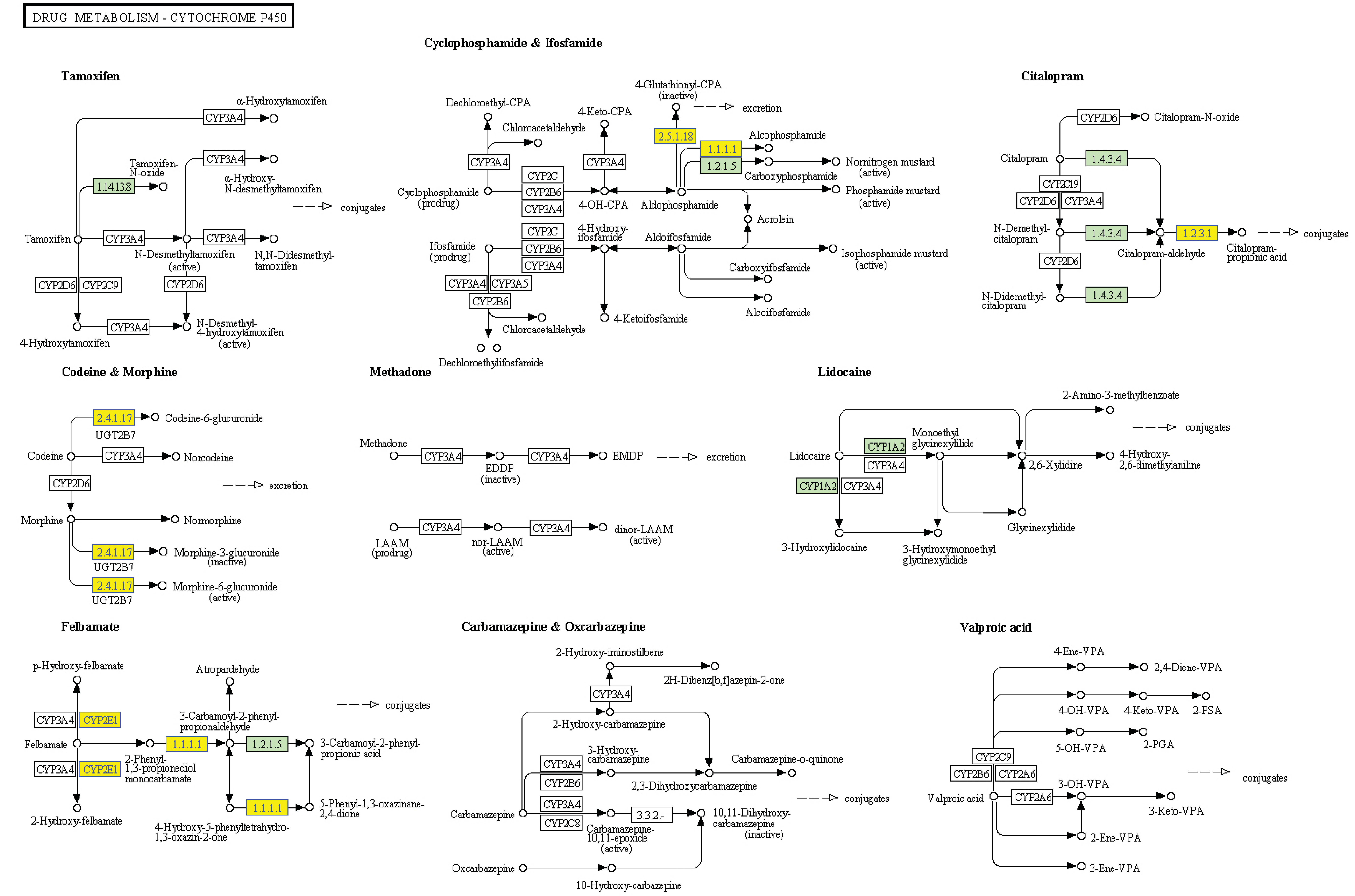

Figure S16

(c) Kanehisa Laboratories

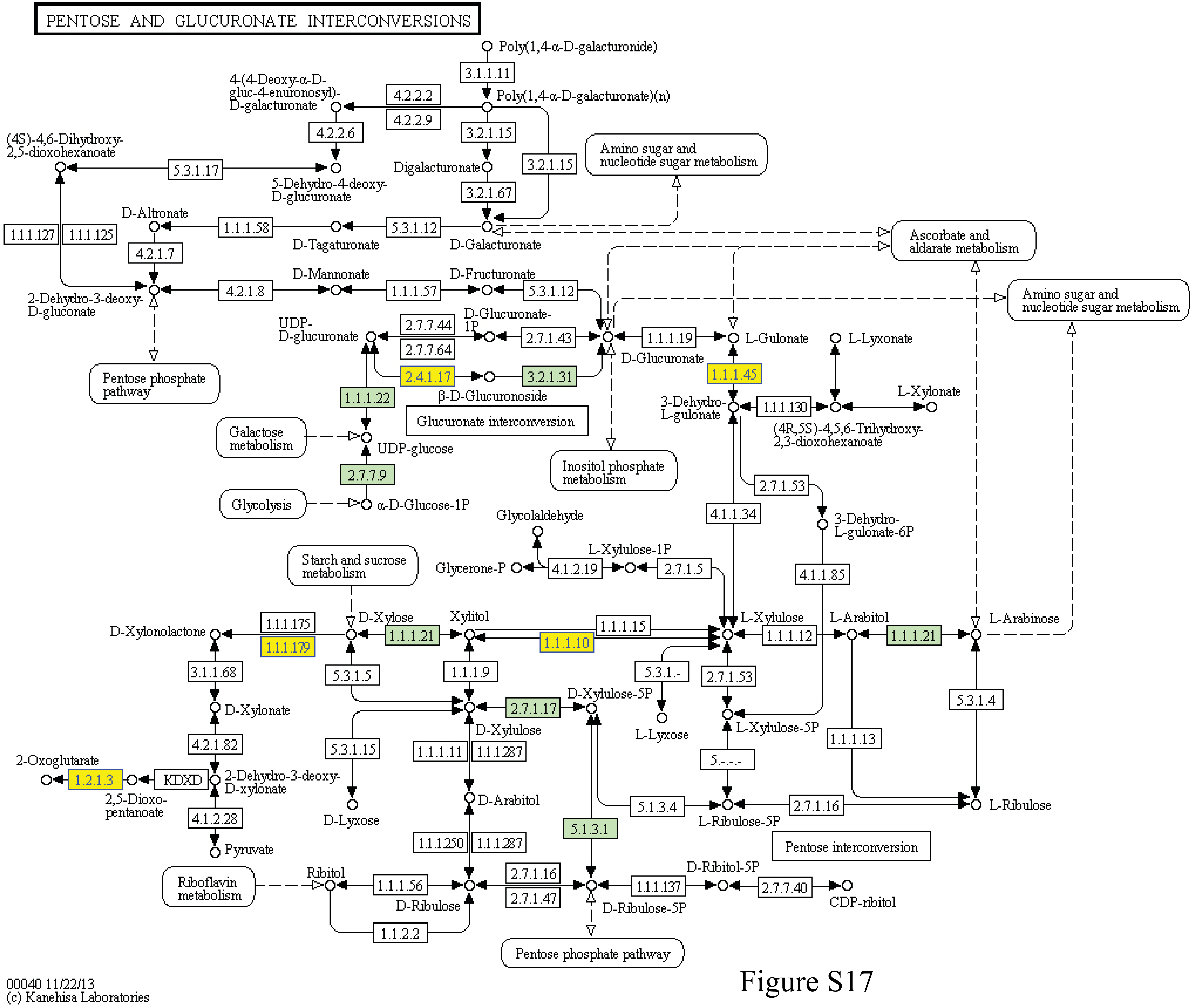
PENTOSE AND GLUCURONATE INTERCONVERSIONS
Poly(1,4-α-D-galacturonide)
3.1.1.11
Poly(1,4-α-D-galacturonate)(n)
4-(4-Deoxy-α-D-gluc-4-enuronosyl)-D-galacturonate
4.2.2.2
4.2.2.9
4.2.2.6
3.2.1.15
3.2.1.15
Amino sugar and nucleotide sugar metabolism
(4S)-4,6-Dihydroxy-2,5-dioxohexanoate
5.3.1.17
5-Dehydro-4-deoxy-D-glucuronate
Digalacturonate
3.2.1.67
D-Altronate
1.1.1.127
1.1.1.125
1.1.1.58
D-Tagaturonate
5.3.1.12
D-Galacturonate
Ascorbate and aldarate metabolism
4.2.1.7
2-Dehydro-3-deoxy-D-gluconate
4.2.1.8
D-Mannonate
1.1.1.57
D-Fructuronate
5.3.1.12
Amino sugar and nucleotide sugar metabolism
UDP-D-glucuronate
2.7.7.44
2.7.7.64
D-Glucuronate-1P
2.7.1.43
1.1.1.19
L-Gulonate
L-Lyxonate
D-Glucuronate
Pentose phosphate pathway
2.4.1.17
3.2.1.31
1.1.1.45
1.1.1.22
β-D-Glucuronoside
L-Xylonate
Glucuronate interconversion
3-Dehydro-L-gulonate
1.1.1.130
(4R,5S)-4,5,6-Trihydroxy-2,3-dioxohexanoate
Galactose metabolism
UDP-glucose
Inositol phosphate metabolism
2.7.7.9
2.7.1.53
Glycolysis
α-D-Glucose-1P
4.1.1.34
3-Dehydro-L-gulonate-6P
Glycolaldehyde
L-Xylulose-1P
Starch and sucrose metabolism
Glycerone-P
4.1.2.19
2.7.1.5
4.1.1.85
D-Xylose
Xylitol
1.1.1.175
1.1.1.15
L-Xylulose
L-Arabitol
L-Arabinose
D-Xylonolactone
1.1.1.179
1.1.1.21
1.1.1.10
1.1.1.12
1.1.1.21
3.1.1.68
5.3.1.5
1.1.1.9
5.3.1.-
2.7.1.53
D-Xylulose-5P
2.7.1.17
5.3.1.4
D-Xylonate
D-Xylulose
L-Lyxose
L-Xylulose-5P
5.3.1.15
4.2.1.82
1.1.1.11
1.1.1.287
1.1.1.13
5.-.-.-
2-Oxoglutarate
1.2.1.3
KDXD
2-Dehydro-3-deoxy-D-xylonate
2,5-Dioxo-pentanoate
D-Lyxose
D-Arabitol
5.1.3.4
2.7.1.16
L-Ribulose
L-Ribulose-5P
4.1.2.28
5.1.3.1
Pentose interconversion
Pyruvate
1.1.1.250
1.1.1.287
D-Ribitol-5P
Riboflavin metabolism
Ribitol
1.1.1.56
2.7.1.16
2.7.1.47
1.1.1.137
2.7.7.40
CDP-ribitol
D-Ribulose
D-Ribulose-5P
1.1.2.2
Pentose phosphate pathway
00040 11/22/13
(c) Kanehisa Laboratories

Figure S17

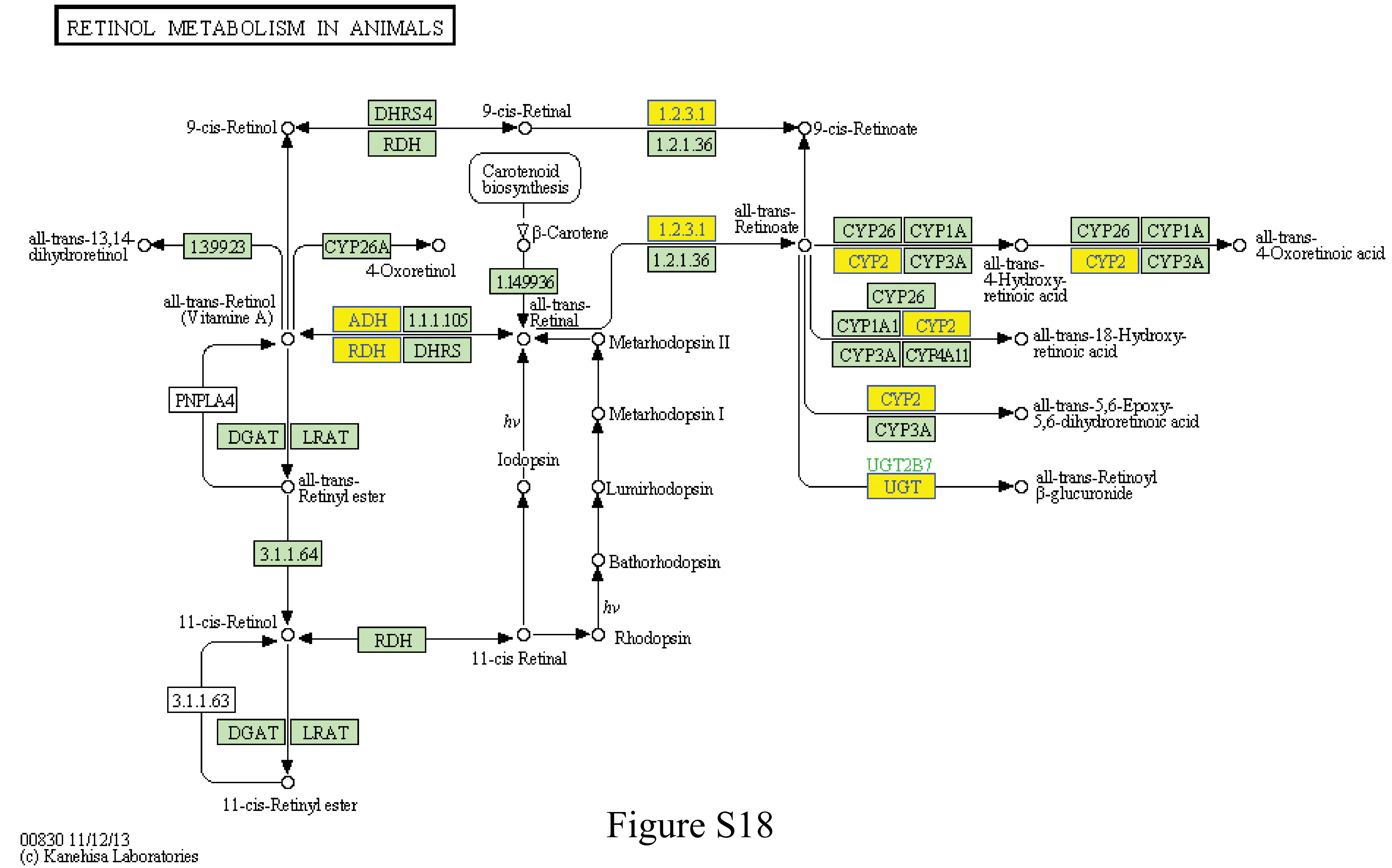
RETINOL METABOLISM IN ANIMALS
9-cis-Retinol
DHRS4
RDH
9-cis-Retinal
1.2.3.1
1.2.1.36
9-cis-Retinoate
Carotenoid biosynthesis
β-Carotene
all-trans-13,14-dihydroretinol
139923
CYP26A
4-Oxoretinol
1.14.99.36
1.2.3.1
1.2.1.36
all-trans-Retinoate
CYP26
CYP1A
CYP2
CYP3A
all-trans-4-Hydroxy-retinoic acid
CYP26
CYP1A
CYP2
CYP3A
all-trans-4-Oxoretinoic acid
all-trans-Retinol (Vitamine A)
ADH
1.1.1.105
RDH
DHRS
all-trans-Retinal
Metarhodopsin II
CYP26
CYP1A1
CYP2
CYP3A
CYP4A11
all-trans-18-Hydroxy-retinoic acid
PNPLA4
DGAT
LRAT
hν
Metarhodopsin I
CYP2
CYP3A
all-trans-5,6-Epoxy-5,6-dihydroretinoic acid
Iodopsin
UGT2B7
UGT
all-trans-Retinoyl β-glucuronide
all-trans-Retinyl ester
Lumirhodopsin
3.1.1.64
Bathorhodopsin
hν
11-cis-Retinol
RDH
11-cis Retinal
Rhodopsin
3.1.1.63
DGAT
LRAT
11-cis-Retinyl ester
00830 11/12/13
(c) Kanehisa Laboratories

Figure S18

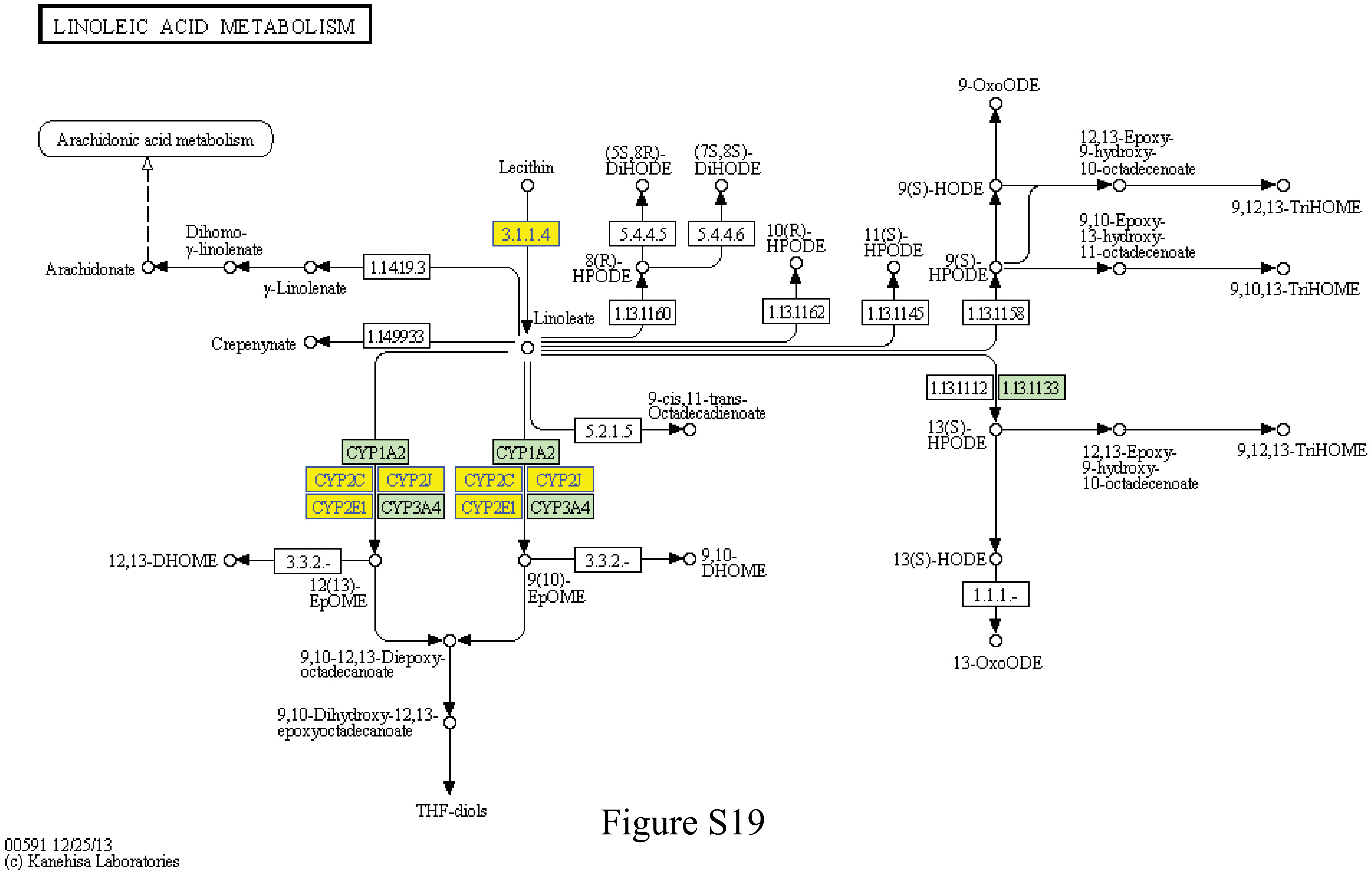
LINOLEIC ACID METABOLISM
Arachidonic acid metabolism
Arachidonate
Dihomo-γ-linolenate
γ-Linolenate
1.14.19.3
Lecithin
3.1.1.4
Linoleate
Crepenynate
1.14.99.33
(5S,8R)-DiHODE
(7S,8S)-DiHODE
5.4.4.5
5.4.4.6
8(R)-HPODE
1.13.11.60
10(R)-HPODE
1.13.11.62
11(S)-HPODE
1.13.11.45
9-OxoODE
9(S)-HODE
9(S)-HPODE
1.13.11.58
12,13-Epoxy-9-hydroxy-10-octadecenoate
9,12,13-TriHOME
9,10-Epoxy-13-hydroxy-11-octadecenoate
9,10,13-TriHOME
1.13.11.12
1.13.11.33
13(S)-HPODE
12,13-Epoxy-9-hydroxy-10-octadecenoate
9,12,13-TriHOME
13(S)-HODE
1.1.1.-
13-OxoODE
9-cis,11-trans-Octadecadienoate
5.2.1.5
CYP1A2
CYP2C
CYP2J
CYP2E1
CYP3A4
CYP1A2
CYP2C
CYP2J
CYP2E1
CYP3A4
12,13-DHOME
3.3.2.-
12(13)-EpOME
9(10)-EpOME
3.3.2.-
9,10-DHOME
9,10-12,13-Diepoxy-octadecanoate
9,10-Dihydroxy-12,13-epoxyoctadecanoate
THF-diols
00591 12/25/13
(c) Kanehisa Laboratories

Figure S19

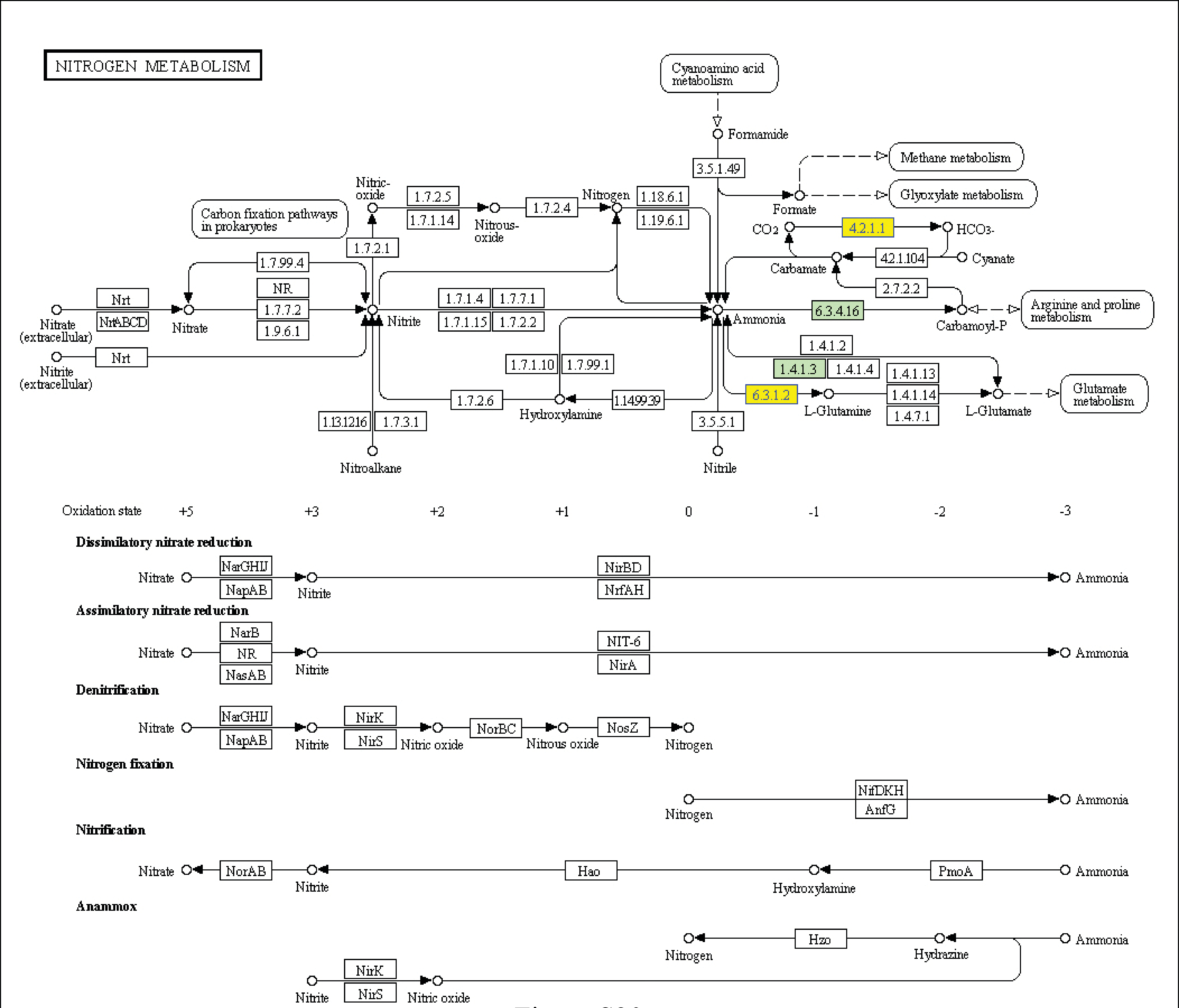

Figure S20

00910 1/9/14
(c) Kanehisa Laboratories

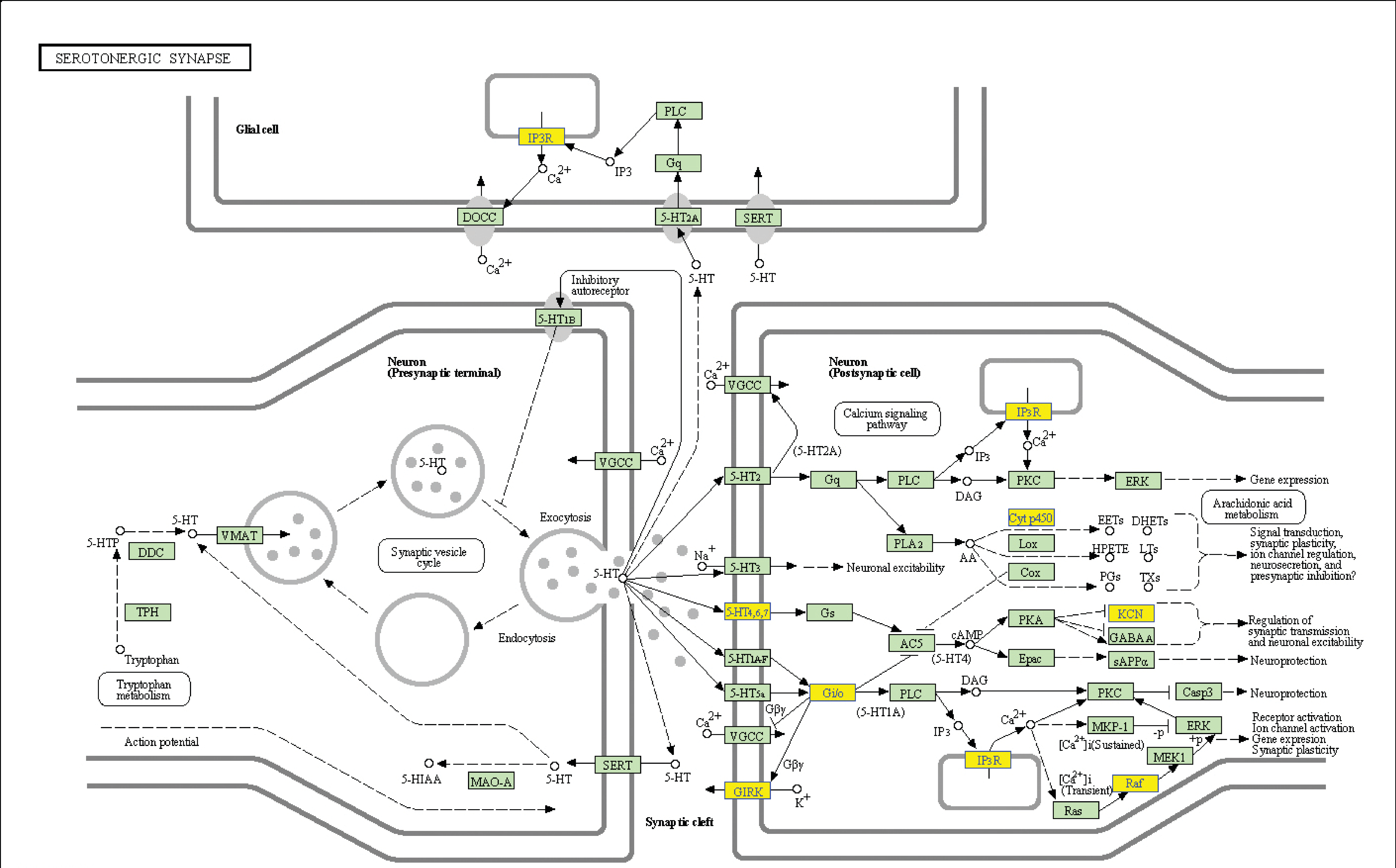
SEROTONERGIC SYNAPSE
Glial cell
IP3R
PLC
Gq
IP3
Ca2+
DOCC
5-HT2A
SERT
5-HT
Inhibitory autoreceptor
5-HT1B
Neuron (Presynaptic terminal)
Neuron (Postsynaptic cell)
VGCC
Calcium signaling pathway
5-HT2
(5-HT2A)
PLA2
AA
DAG
PKC
ERK
Gene expression
Cyt p450
Lox
Cox
EETs
DHETs
HPETE
LTs
PGs
TXs
Arachidonic acid metabolism
Signal transduction, synaptic plasticity, ion channel regulation, neurosecretion, and presynaptic inhibition?
5-HT3
Na+
Neuronal excitability
5-HT4,6,7
Gs
AC5
cAMP
(5-HT4)
PKA
Epac
KCN
GABAA
sAPPα
Regulation of synaptic transmission and neuronal excitability
Neuroprotection
5-HT1A-F
5-HT5a
Gi/o
(5-HT1A)
Gβγ
Casp3
MKP-1
MEK1
Raf
Ras
[Ca2+]i(Sustained)
[Ca2+]i (Transient)
Receptor activation
Ion channel activation
Gene expression
Synaptic plasticity
GIRK
K+
VMAT
5-HTP
DDC
TPH
Tryptophan
Tryptophan metabolism
Synaptic vesicle cycle
Exocytosis
Endocytosis
Action potential
5-HIAA
MAO-A
Synaptic cleft
04726 11/11/13
(c) Kanehisa Laboratories

Figure S21

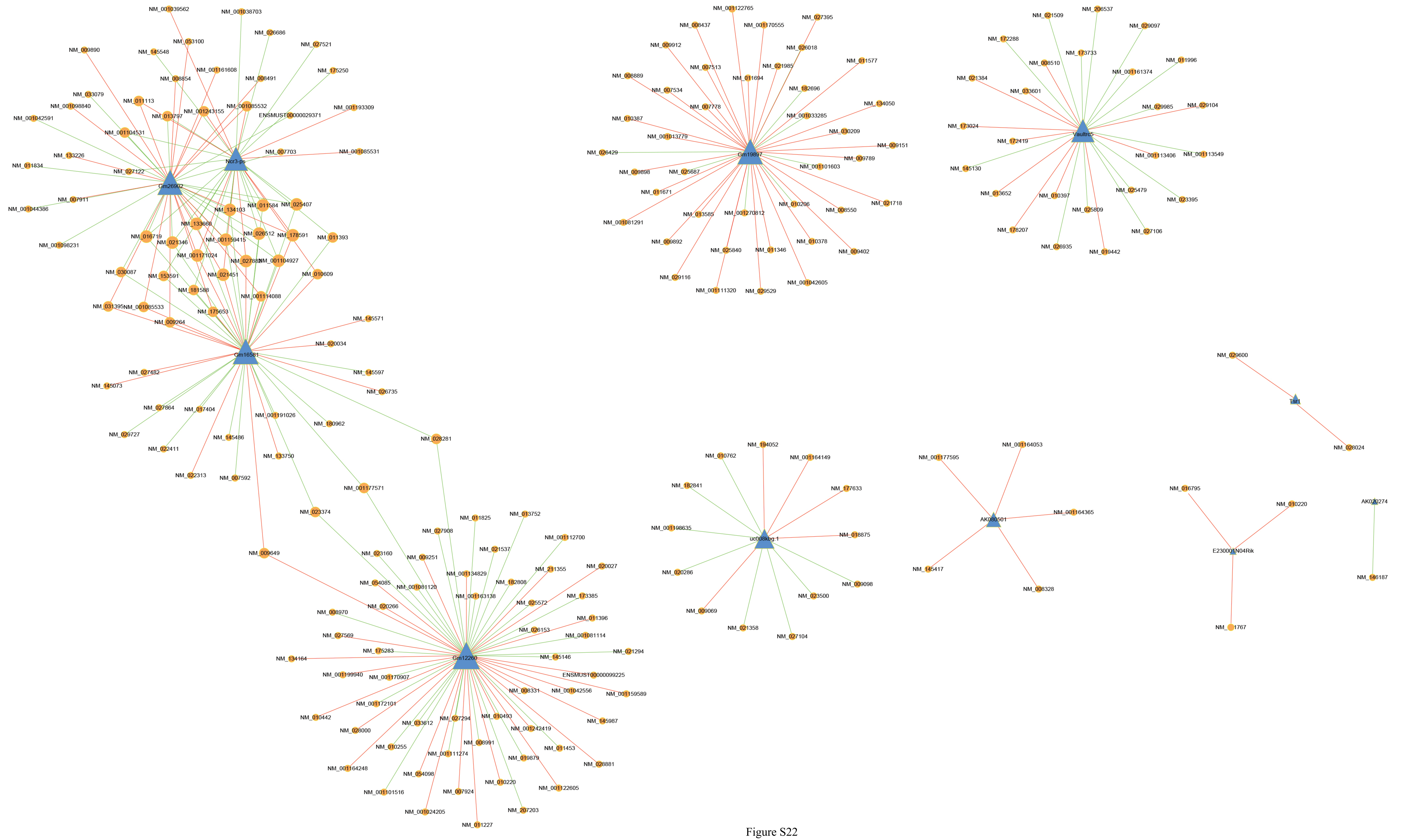
Ncr3-ps
Gm26902
Gm16581
Gm12260
Gm19897
Vaultrc5
Tsr1
uc008kbg.1
AK080501
E230001N04Rik
AK020274
NM_001039562
NM_001038703
NM_026686
NM_053100
NM_027521
NM_009890
NM_145548
NM_001161608
NM_175250
NM_008854
NM_008491
NM_033079
NM_011113
NM_001085532
NM_001193309
NM_001098840
NM_001243155
NM_013797
ENSMUST00000029371
NM_001042591
NM_001104531
NM_007703
NM_001085531
NM_133226
NM_011834
NM_027122
NM_007911
NM_011584
NM_025407
NM_001044386
NM_134103
NM_133660
NM_026512
NM_178591
NM_011393
NM_016719
NM_001159415
NM_021346
NM_001098231
NM_001171024
NM_027883
NM_001104927
NM_030087
NM_021451
NM_010609
NM_153591
NM_181588
NM_001114088
NM_031395
NM_001085533
NM_175653
NM_009264
NM_145571
NM_020034
NM_027482
NM_145597
NM_145073
NM_026735
NM_027864
NM_017404
NM_001191026
NM_180962
NM_029727
NM_145486
NM_028281
NM_022411
NM_133750
NM_022313
NM_007592
NM_001177571
NM_023374
NM_009649
NM_011825
NM_013752
NM_027908
NM_001112700
NM_021537
NM_023160
NM_009251
NM_211355
NM_020027
NM_001134829
NM_054085
NM_182808
NM_001081120
NM_001163138
NM_173385
NM_025572
NM_020266
NM_011396
NM_008970
NM_026153
NM_001081114
NM_027569
NM_021294
NM_175283
NM_145146
NM_134164
ENSMUST00000099225
NM_001199940
NM_001170907
NM_008331
NM_001042556
NM_001159589
NM_001172101
NM_010442
NM_027294
NM_010493
NM_033612
NM_001242419
NM_145987
NM_028000
NM_008991
NM_010255
NM_011453
NM_001111274
NM_019879
NM_028881
NM_001164248
NM_054098
NM_010220
NM_001101516
NM_007924
NM_001122605
NM_207203
NM_001024205
NM_011227
NM_001122765
NM_027395
NM_008437
NM_001170555
NM_009912
NM_026018
NM_007513
NM_021985
NM_011577
NM_008889
NM_011694
NM_007534
NM_182696
NM_134050
NM_007778
NM_001033285
NM_010387
NM_030209
NM_001013779
NM_009151
NM_026429
NM_009789
NM_009898
NM_025687
NM_001101603
NM_011671
NM_021718
NM_010206
NM_008550
NM_001081291
NM_013585
NM_001270812
NM_009892
NM_010378
NM_025840
NM_011346
NM_009402
NM_029116
NM_001042605
NM_001111320
NM_029529
NM_206537
NM_021509
NM_029097
NM_172288
NM_173733
NM_008510
NM_011996
NM_001161374
NM_021384
NM_033601
NM_029985
NM_029104
NM_173024
NM_172419
NM_001113406
NM_001113549
NM_145130
NM_013652
NM_010397
NM_025479
NM_023395
NM_025809
NM_178207
NM_027106
NM_026935
NM_019442
NM_029600
NM_028024
NM_194052
NM_010762
NM_001164149
NM_182841
NM_177633
NM_001198635
NM_018875
NM_020286
NM_009098
NM_023500
NM_009069
NM_021358
NM_027104
NM_001177595
NM_001164053
NM_001164365
NM_145417
NM_008328
NM_016795
NM_010220
NM_ 1767
NM_146187

Figure S22
