# Supplementary material for: Aberrant Expression Profiles of lncRNAs and Their Associated Nearby Coding Genes in the Hippocampus of the SAMP8 Mouse Model with AD
Source: Mol Ther Nucleic Acids. 2020 Feb 19;20:140–54. doi: 10.1016/j.omtn.2020.02.008 (PMC7066064; doi:10.1016/j.omtn.2020.02.008)
Supplement: Document S2. Article plus Supplemental Information [file mmc19.pdf]

Molecular Therapy
Nucleic Acids
Original Article

Aberrant Expression Profiles of lncRNAs and Their Associated Nearby Coding Genes in the Hippocampus of the SAMP8 Mouse Model with AD

Honghai Hong,[1,2,7] Yousheng Mo,[3,7] Dongli Li,[3,7] Zhiheng Xu,[4] Yanfang Liao,[3] Ping Yin,[1] Xinning Liu,[3] Yong Xia,[1] Jiansong Fang,[3,5] Qi Wang,[3,5] and Shuhuan Fang[3,5,6]

[1]Department of Clinical Laboratory, The Third Affiliated Hospital of Guangzhou Medical University, 63 Duobao Road, Guangzhou, Guangdong Province, China; [2]Department of Pathology and Laboratory Medicine, Indiana University School of Medicine, Indianapolis, IN 46202, USA; [3]Science and Technology Innovation Center, Guangzhou University of Chinese Medicine, Guangzhou, Guangdong Province, China; [4]Department of Gastrointestinal Surgery, The First Affiliated Hospital of Guangzhou University of Chinese Medicine, Guangzhou, Guangdong Province, China; [5]DME Center, Institute of Clinical Pharmacology, Guangzhou University of Chinese Medicine, Guangzhou, Guangdong Province, China; [6]Department of Cardiothoracic Surgery, Stanford University School of Medicine, Stanford, CA, USA

The senescence-accelerated mouse prone 8 (SAMP8) mouse model is a useful model for investigating the fundamental mechanisms involved in the age-related learning and memory deficits of Alzheimer's disease (AD), while the SAM/resistant 1 (SAMR1) mouse model shows normal features. Recent evidence has shown that long non-coding RNAs (lncRNAs) may play an important role in AD pathogenesis. However, a comprehensive and systematic understanding of the function of AD-related lncRNAs and their associated nearby coding genes in AD is still lacking. In this study, we collected the hippocampus, the main area of AD pathological processes, of SAMP8 and SAMR1 animals and performed microarray analysis to identify aberrantly expressed lncRNAs and their associated nearby coding genes, which may contribute to AD pathogenesis. We identified 3,112 differentially expressed lncRNAs and 3,191 differentially expressed mRNAs in SAMP8 mice compared to SAMR1 mice. More than 70% of the deregulated lncRNAs were intergenic and exon sense-overlapping lncRNAs. Gene Ontology (GO) and pathway analyses of the AD-related transcripts were also performed and are described in detail, which imply that metabolic process reprograming was likely related to AD. Furthermore, six lncRNAs and six mRNAs were selected for further validation of the microarray results using quantitative PCR, and the results were consistent with the findings from the microarray. Moreover, we analyzed 780 lincRNAs (also called long "intergenic" non-coding RNAs) and their associated nearby coding genes. Among these lincRNAs, AK158400 had the most genes nearby (n = 13), all of which belonged to the histone cluster 1 family, suggesting regulation of the nucleosome structure of the chromosomal fiber by affecting nearby genes during AD progression. In addition, we also identified 97 aberrant antisense lncRNAs and their associated coding genes. It is likely that these dysregulated lncRNAs and their associated nearby coding genes play a role in the development and/or progression of AD.

INTRODUCTION

Alzheimer's disease (AD) is considered an age-related neurodegenerative disease with a progressive impairment in cognitive function that is characterized by the presence of senile plaques and neurofibrillary tangles.[1,2] The hippocampus is one of the most important brain regions for learning and memory and is the main impaired region of AD.[3] The progressive memory deterioration of AD results in the loss of autonomy, and patients ultimately require full-time medical care.[4] Unfortunately, there are no effective therapeutic strategies to prevent the progression of AD currently.[5] Therefore, revealing the molecular mechanism of AD is necessary for developing effective therapy. The debate over whether senile plaques and neurofibrillary tangles are causative or merely markers of the disease has been ongoing for most of the past century. Hence, despite considerable research, including studies of various genes and proteins in this area, the detailed mechanism of AD is still limited, and we should focus on molecules other than genes and proteins that may play an important role in AD.

Long non-coding RNAs (lncRNAs), a novel kind of non-coding RNA that ranges from 200 nt to more than 100 kb and usually

Received 4 March 2019; accepted 11 February 2020;
https://doi.org/10.1016/j.omtn.2020.02.008.

[7]These authors contributed equally to this work.

Correspondence: Shuhuan Fang, Science and Technology Innovation Center, Guangzhou University of Chinese Medicine, Guangzhou, Guangdong Province, China; Department of Cardiothoracic Surgery, Stanford University School of Medicine, Stanford, CA, USA.
E-mail: fangshuhuan@gzucm.edu.cn
Correspondence: Wang Qi, Science and Technology Innovation Center, Guangzhou University of Chinese Medicine, Guangzhou, Guangdong Province, China.
E-mail: 1055898390@qq.com
Correspondence: Jiansong Fang, Science and Technology Innovation Center, Guangzhou University of Chinese Medicine, Guangzhou, Guangdong Province, China.
E-mail: fangjs@gzucm.edu.cn

 © 2020 The Authors.
This is an open access article under the CC BY license (http://creativecommons.org/licenses/by/4.0/).

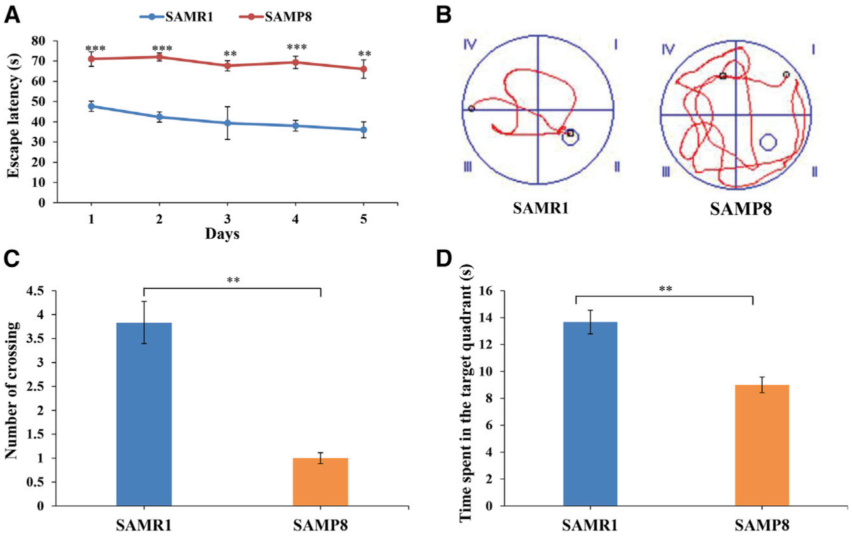

Figure 1. Learning and Memory Abilities of SAMP8 Mice

(A) Escape latency of SAMP8 (n = 10) mice and SAMR1 (n = 10) littermates in the Morris water maze test. $^{**}p < 0.01$, $^{***}p < 0.001$. (B) Drawings are representations of single-mouse distances to the platform location. (C) Number of crossings in the probe trial test. $^{**}p < 0.01$. (D) Time spent in the target quadrant in the probe trial test. $^{**}p < 0.01$.

lacks an obvious open reading frame, have received increasing attention for their involvement in the pathogenesis of many diseases.[6–8] Emerging data strongly suggest that lncRNAs are important for the basal regulation of protein coding genes at the transcriptional and posttranscriptional levels.[9] Abnormalities among lncRNAs, in regard to their sequence, spatial structure, expression, and interaction with proteins, have been found to play important roles in AD pathogenesis.[10,11] Research has shown that β-site amyloid precursor protein (APP)-cleaving enzyme-1 (BACE1) antisense transcript (BACE1-AS) regulates BACE1 expression at both the mRNA and protein levels, which could enhance APP processing and $A\beta_{1-42}$ production as well as plaque deposition.[12,13] 51A is a novel lncRNA that is overexpressed in *in vitro* models and the brain of individuals with AD, which was shown to regulate the expression of alternatively spliced SORL1 variants and subsequently increase amyloid formation.[14,15] 17A, NDM29, and NAT-Rad18 were also reported to be involved in the mechanism of AD.[16–18] However, until now, only a few studies have examined the roles of lncRNAs in AD, and our understanding of AD-associated lncRNAs has been limited to preliminary explorations. Thus, the identification of the genome-wide expression and the functional significance of AD-associated lncRNAs and their associated nearby coding genes is necessary.

In the present study, we utilized microarray technology to analyze the expression profiles of lncRNAs and mRNAs in the hippocampus of 8-month-old senescence-accelerated mouse (SAM) prone 8 (SAMP8) mice with AD and age-matched SAM/resistant 1 (SAMR1) mice. The aim of this study was to systematically explore the lncRNA and mRNA expression profiles, the related pathways, and the associated nearby coding genes of the lncRNAs, all of which may contribute to the understanding of AD pathogenesis and provide a valuable resource for the diagnosis and therapy of AD in the clinic.

RESULTS

Learning and Memory Abilities of SAMP8 Mice

To evaluate the learning and memory abilities of 8-month-old SAMP8 mice, we performed the Morris water maze test. Compared to age-matched SAMR1 mice, 8-month-old SAMP8 mice exhibited obviously increased escape latencies and traveled a greater distance before finding the hidden platform (Figures 1A and 1B), implying that the AD model mice had worse learning performances. Additionally, in the probe test, the number of platform crossings and time spent in the target quadrant of the SAMP8 mice were significantly reduced compared to SAMR1 mice (Figures 1C and 1D). Taken together, these results indicated that 8-month-old SAMP8 mice exhibited severe learning and cognitive impairments and spontaneously developed AD, which was also consistent with our previous studies.[19]

Overview of the lncRNA Expression Profiles in Hippocampal Tissues of 8-Month-Old SAMP8 Mice Compared to Age-Matched SAMR1 Mice

We used an Arraystar mouse lncRNA microarray v3.0 to analyze the expression profiles of lncRNAs in four pairs of hippocampal tissues from SAMP8 and SAMR1 mice. The lncRNA expression patterns of the hippocampal tissues of the two groups of mice were classified by using hierarchical clustering and boxplots, as illustrated in Figures 2A and 2B. A scatterplot and volcano plot depicting the variation in lncRNA expression in the two groups are shown in Figures 2C and 2D. The results showed that a total of 21,314 aberrantly expressed lncRNAs were identified in the hippocampal tissues of SAMP8 mice compared with age-matched SAMR1, of which 9,639 lncRNAs had upregulated expression and 11,675 displayed downregulated expression (Tables S1 and S2) in the SAMP8 mice. When utilizing a fold change and p value cutoff >2.0 and <0.05, respectively, it was found that 1,140 lncRNAs (the length and fold change ranged from 60 to 27,759 and 2 to 125.9, respectively) had upregulated expression, and 1,972 lncRNAs (the length and fold change ranged from 61 to 117,168 and 2 to 101.3, respectively) displayed downregulated expression, among the 3,112 differentially expressed lncRNAs (Figure 2E; Table S3). The lncRNAs were carefully identified with the use of the most authoritative databases, such as RefSeq, UCSC_knowngene, Ensembl, UCR, lincRNA, and many related studies (Figure S1). The number of dysregulated lncRNAs varied among the subgroups with different fold changes, with most of the

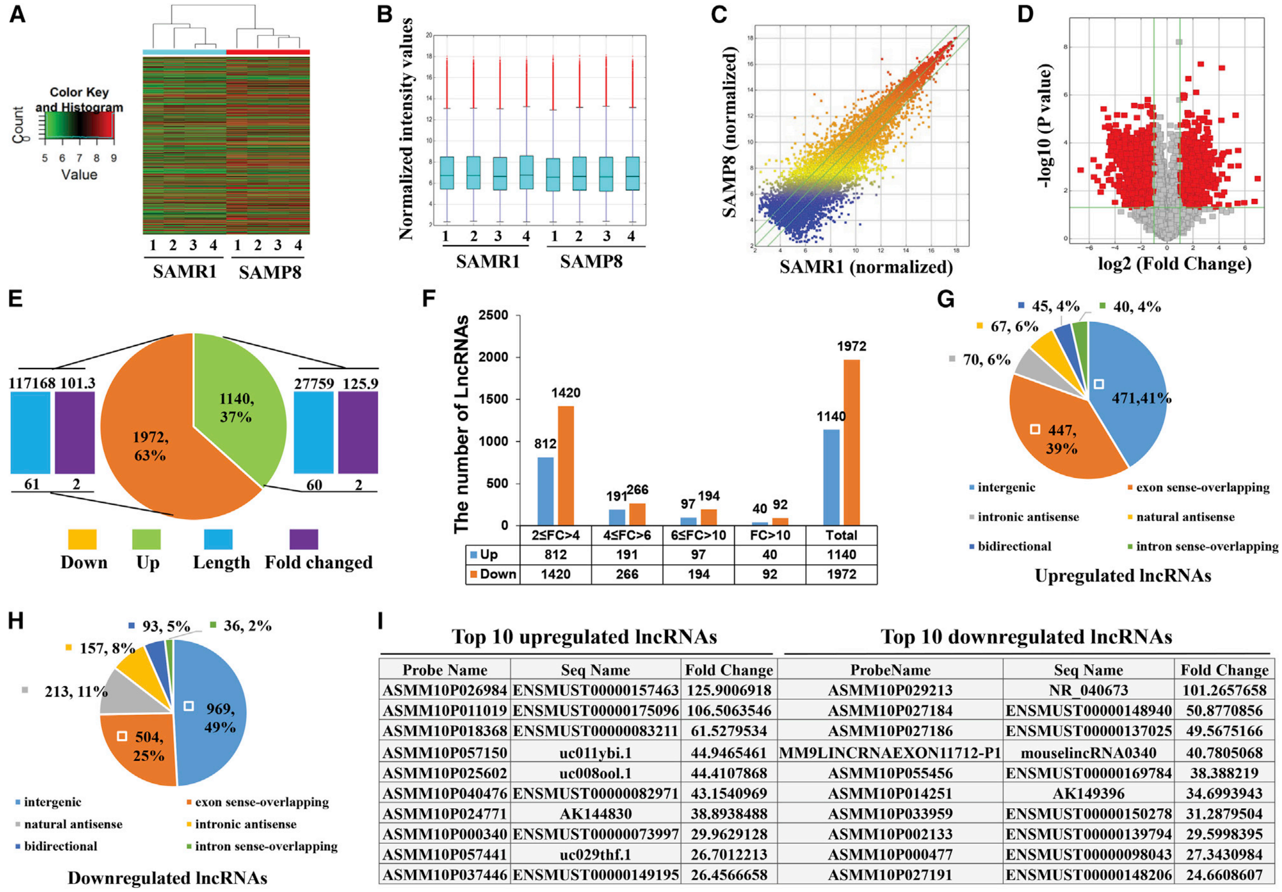

Probe Name	Seq Name	Fold Change	ProbeName	Seq Name	Fold Change
ASMM10P026984	ENSMUST00000157463	125.9006918	ASMM10P029213	NR_040673	101.2657658
ASMM10P011019	ENSMUST00000175096	106.5063546	ASMM10P027184	ENSMUST00000148940	50.8770856
ASMM10P018368	ENSMUST00000083211	61.5279534	ASMM10P027186	ENSMUST00000137025	49.5675166
ASMM10P057150	uc011ybi.1	44.9465461	MM9LINCRNAEXON11712-P1	mouselincRNA0340	40.7805068
ASMM10P025602	uc008ool.1	44.4107868	ASMM10P055456	ENSMUST00000169784	38.388219
ASMM10P040476	ENSMUST00000082971	43.1540969	ASMM10P014251	AK149396	34.6993943
ASMM10P024771	AK144830	38.8938488	ASMM10P033959	ENSMUST00000150278	31.2879504
ASMM10P000340	ENSMUST00000073997	29.9629128	ASMM10P002133	ENSMUST00000139794	29.5998395
ASMM10P057441	uc029thf.1	26.7012213	ASMM10P000477	ENSMUST00000098043	27.3430984
ASMM10P037446	ENSMUST00000149195	26.4566658	ASMM10P027191	ENSMUST00000148206	24.6608607

Figure 2. Overview of lncRNA Expression Profiles in Hippocampal Tissues of 8-Month-Old SAMP8 Mice Compared with Age-Matched SAMR1 Mice

(A) Hierarchical clustering of differentially expressed lncRNAs. Green indicates low intensity, black indicates medium intensity, and red indicates strong intensity. (B) Boxplot of differentially expressed lncRNAs in each group. (C) Scatterplot of lncRNA signal values visualizing the variation (or reproducibility) between the two groups. The green lines represent the fold change lines. The lncRNAs above the top green line and below the bottom green line demonstrated more than a 2-fold change of lncRNA expression between the two compared samples. (D) Volcano plot of the differential expression of lncRNAs. The vertical lines correspond to 2-fold upregulation and downregulation, respectively. The horizontal line represents a p value of 0.05, and the red points on the plot represent the differentially expressed lncRNAs with statistical significance. (E) Pie chart shows the number of upregulated and downregulated lncRNAs with the length and fold change. Green indicates upregulated lncRNA, orange indicates downregulated lncRNA, blue bar indicates length of lncRNA, and brown bar indicates fold change of lncRNA. (F) Number of lncRNAs in the different subgroups classified by fold change (FC). Blue and orange bars indicate the number of upregulated and downregulated lncRNAs, respectively. (G and H) Pie chart shows the classification of the lncRNAs. According the genomic positions, upregulated (G) and downregulated (H) lncRNAs were classified as bidirectional, exon sense-overlapping, intergenic, intron sense-overlapping, intronic antisense, and natural antisense. (I) Top 10 significantly upregulated and downregulated lncRNAs in the microarray data.

lncRNAs falling in the 2 ≤ fold change < 4 subgroup (Figure 2F). According to the various genomic positions of the lncRNAs with respect to other genes, all of the aberrantly expressed lncRNAs were classified as one of the following six types: bidirectional, exon sense-overlapping, intergenic, intron sense-overlapping, intronic antisense, and natural antisense. We found that more than 70% of the dysregulated lncRNAs belonged to the intergenic and exon sense-overlapping groups, regardless of whether the lncRNAs were upregulated (Figure 2G) or downregulated (Figure 2H). In addition, the most upregulated lncRNA, ENSMUST00000157463 (fold change, 125.9), and the most downregulated lncRNA, ASMM10P029213 (fold change, 101.3), as well as the top 10 distinctly regulated lncRNAs, are shown in Figure 2I. These lncRNAs may contribute to AD pathogenesis.

Expression Profiles of mRNAs in Hippocampal Tissues of 8-Month-Old SAMP8 Mice Compared to Age-Matched SAMR1 Mice

A boxplot was generated to show the differential mRNA expression patterns among the hippocampal samples of SAMP8 and SAMR1 mice (Figure 3A). A scatterplot and volcano plot were used to visually assess the variation between the two groups (Figures 3B and 3C). The mRNA expression profile data from the microarray analysis contained a total of 17,922 mRNAs that were differentially expressed in the hippocampal tissues, of which 9,309 were upregulated and 8,613 were downregulated (Tables S4 and S5). When a fold change cutoff >2.0 and p value cutoff <0.05 were used, 1,880 mRNAs (the length and fold change ranged from 294 to 23,252 and 2 to 72.8, respectively) had upregulated expression,

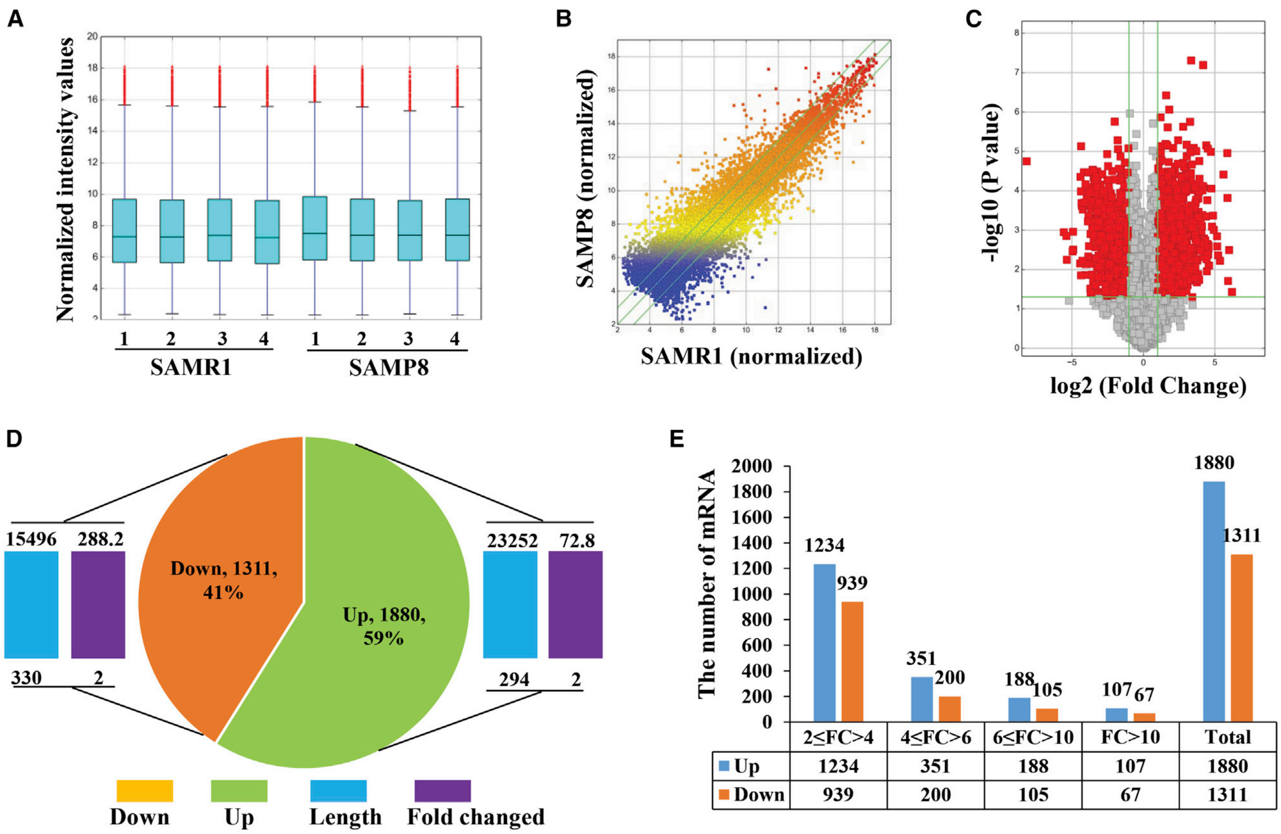

F

Top 10 upregulated mRNAs / **Top 10 downregulated mRNAs**

Probe Name	Seq Name	Gene Symbol	Fold Change	Probe Name	Seq Name	Gene Symbol	Fold Change
ASMM10P053325	NM_001005847	Aga	72.8308372	ASMM10P019947	NM_007703	Elovl3	288.1692927
ASMM10P052419	NM_178206	Hist1h3h	63.4408249	ASMM10P030593	NM_133894	Ugt2b38	47.5958085
ASMM10P054515	NM_010922	Mrpl40	60.3199196	ASMM10P027185	NM_001126319	Mup9	41.9690798
ASMM10P002419	NM_008491	Lcn2	58.6343678	ASMM10P038756	NM_024228	Gdpd3	40.965831
ASMM10P025219	NM_009892	Chil3	58.0367972	ASMM10P033215	NM_009349	Inmt	30.9108142
ASMM10P019154	NM_009890	Ch25h	48.181388	ASMM10P049924	NM_011867	Slc26a4	30.3595778
ASMM10P036113	NM_011315	Saa3	40.858094	ASMM10P024517	NM_007825	Cyp7b1	30.1280992
ASMM10P014367	NM_001033239	Csta	37.0459834	ASMM10P019186	NM_010002	Cyp2c38	21.6012007
ASMM10P006833	NM_009140	Cxcl2	35.6497419	ASMM10P021779	NM_001001444	Defb29	21.1503297
ASMM10P006945	NM_009789	S100g	35.2832934	ASMM10P042633	NM_146419	Olfr883	20.7202305

Figure 3. Differential Expression Profiles of mRNAs

(A–C) Boxplot (A), scatterplot (B), and volcano plot (C) of the differentially expressed mRNAs in the two groups that were compared. (D) The pie chart shows the number of upregulated and downregulated mRNAs with the length and fold change. Green indicates upregulated mRNA, orange indicates downregulated mRNA, blue bar indicates length of mRNA, and brown bar indicates fold change of mRNA. (E) Number of mRNAs in the different subgroups classified by fold change (FC). Blue and orange bars indicate the number of upregulated and downregulated mRNAs, respectively. (F) Top 10 significantly upregulated and downregulated mRNAs in the microarray data.

and 1,311 mRNAs (the length and fold change ranged from 330 to 15,496 and 2 to 228.2, respectively) displayed downregulated expression, among the 3,191 differentially expressed genes in SAMP8 mice compared with SAMR1 mice (Figure 3D; Table S6). Similar to the lncRNAs, most dysregulated mRNAs were in the 2 $\leq$ fold change < 4 subgroup (Figure 3E). In addition, the top 10 differentially regulated mRNAs were identified. The most obviously upregulated mRNA was Aga (NM_001005847; fold change, 72.8), while the most downregulated mRNA was Elovl3 (NM_007703; fold change, 288.2) (Figure 3F).

GO Analysis: The Differential Expression of mRNAs in the Hippocampus of SAMP8 Mice Compared to SAMR1 Mice

The Gene Ontology (GO) analysis covers three domains: biological process, cellular component, and molecular function. A Fisher's exact test was used to determine whether there was more overlap

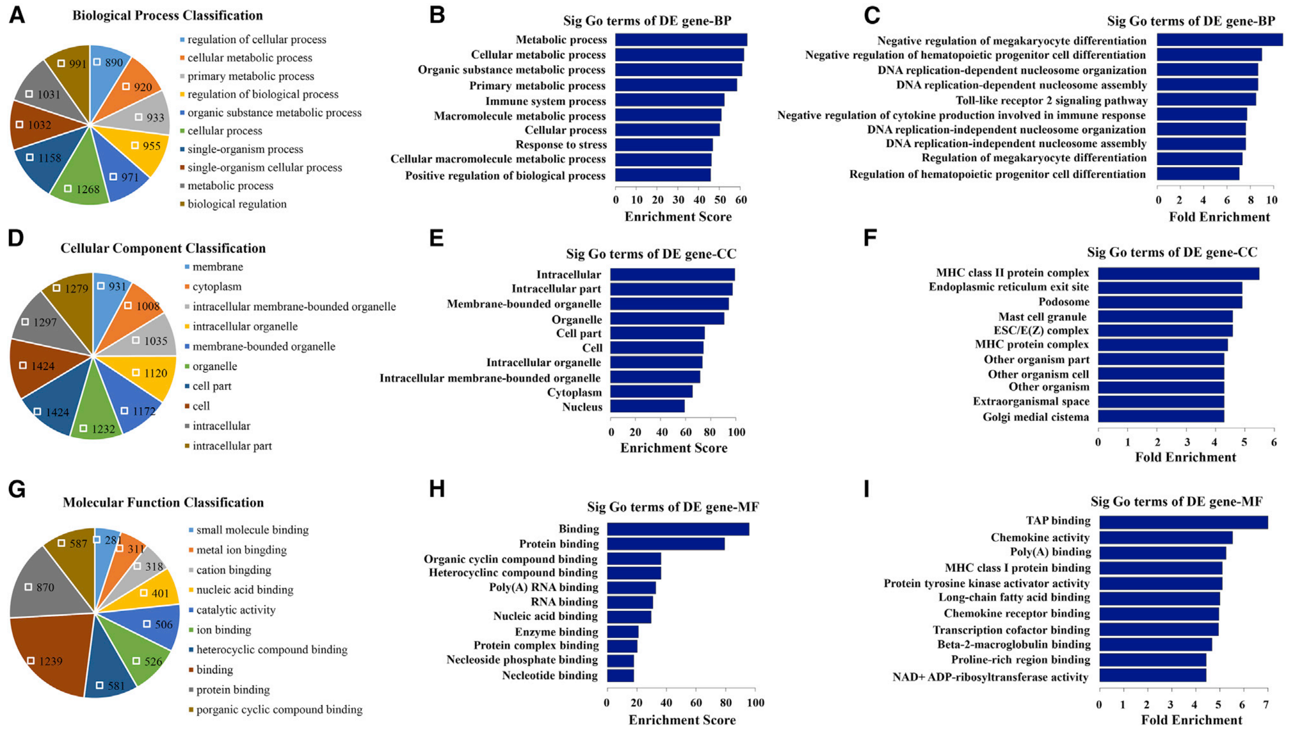

Figure 4. GO Analysis of Differentially Upregulated Expression of mRNAs

(A, D, and G) Pie chart shows the top 10 significant enrichment terms. (B, E, and H) Bar plot shows the top 10 enrichment scores ($-\log_{10}$ (p value)). (C, F, and I) Bar plot shows the top 10 fold enrichment values ((count/pop. hits)/(list. total/pop. total)). "Count" stands for the number of DE genes associated with the listed ID of gene ontology term; "Pop.Hits" stands for the number of background population genes associated with the listed ID of gene ontology term; "List.Total" stands for the total number of DE genes; "Pop.Total" stands for the total number of background population genes. (A–C) biological process (BP). (D–F) Cellular component (CC). (G–I) Molecular function (MF).

between the differentially expressed gene list and the GO annotation list than would be expected by chance. The p value denotes the significance of GO term enrichment among the differentially expressed genes. As the p value decreases, the enrichment of the GO term becomes more significant ($p \leq 0.05$ is recommended). GO analysis, including biological process, cellular component, and molecular function, for upregulated and downregulated mRNAs is shown in Tables S7, S8, S9, S10, S11, and S12. The pie chart shows the top 10 significant enrichment terms (Figures 4A, 4D, 4G, 5A, 5D, and 5G). The bar plot shows the top 10 enrichment scores (Figures 4B, 4E, 4H, 5B, 5E, and 5H) and fold enrichment (Figures 4C, 4F, 4I, 5C, 5F, and 5I) values of the enrichment terms. In our survey of existing data, we found that the GO terms of the upregulated mRNAs in the biological process category were mainly involved in (1) metabolic process, (2) cellular metabolic process, (3) organic substance metabolic process, (4) primary metabolic process, and (5) immune system process (Figure 4B). The GO terms of the downregulated mRNAs under biological process were mainly involved in (1) oxidation-reduction process, (2) single-organism metabolic process, (3) oxoacid metabolic process, (4) organic acid metabolic process, and (5) carboxylic acid metabolic process (Figure 5B). These results indicated that metabolic process reprogramming was likely related to AD.

Pathway Analysis: Differentially Expressed mRNAs in the Hippocampus of SAMP8 Mice Compared to SAMR1 Mice

Pathway analysis is a functional analysis that maps genes to KEGG (Kyoto Encyclopedia of Genes and Genomes) pathways. The pathway analysis for upregulated and downregulated mRNAs is shown in Tables S13 and S14. The results showed that the numbers of upregulated and downregulated pathways were 77 and 37, respectively (Figure 6A; Tables S13 and S14). The bar plot shows the top 10 enrichment scores ($-\log_{10}$ (p value)) of the significantly enriched pathways (Figures 6B and 6C). Overall, we found that the upregulated mRNAs were mainly involved in the following pathways: (1) viral carcinogenesis, (2) tumor necrosis factor (TNF) signaling pathway, (3) transcriptional misregulation in cancer, (4) chemokine signaling pathway; and (5) systemic lupus erythematosus (Figure 6B). The downregulated mRNAs were mainly involved the following pathways: (1) chemical carcinogenesis, (2) steroid hormone biosynthesis, (3) metabolism of xenobiotics by cytochrome P450, (4) drug metabolism (other enzymes), and (5) drug metabolism (cytochrome P450) (Figure 6C), indicating that

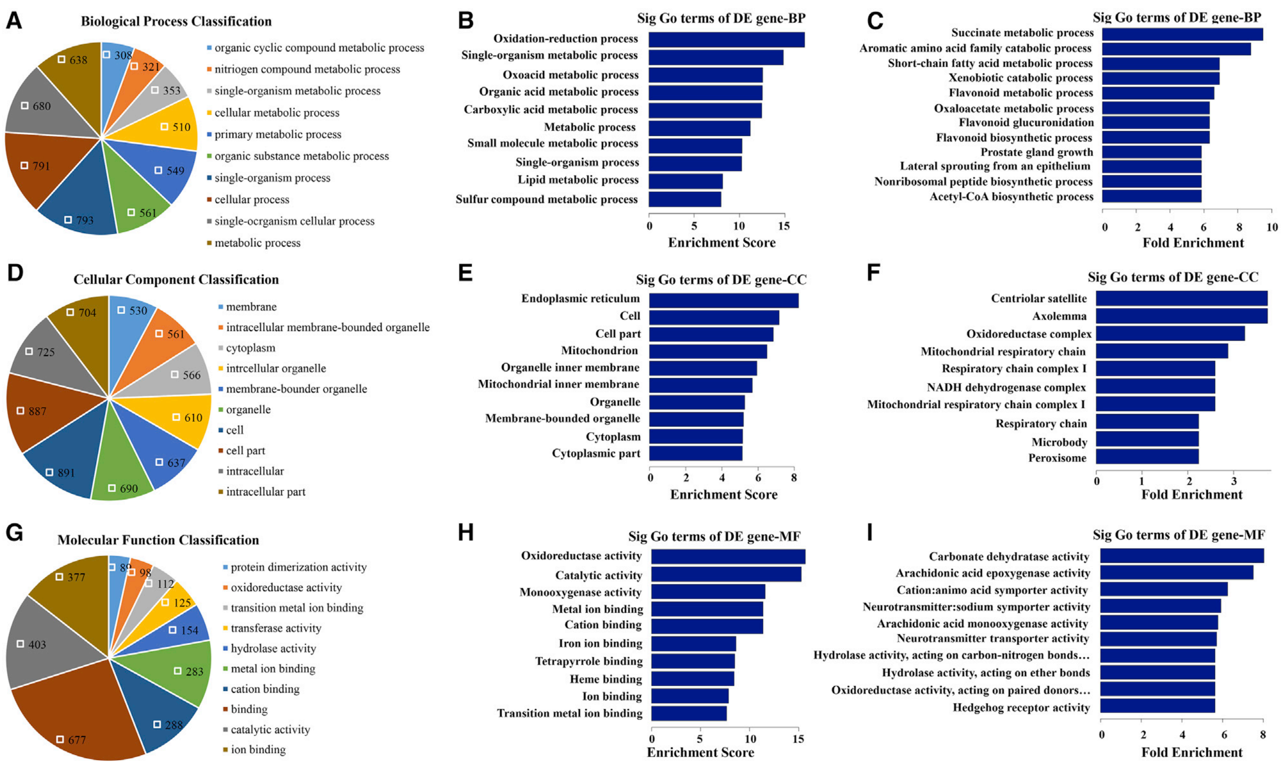

Figure 5. GO Analysis of Differentially Downregulated mRNAs

(A, D, and G) Pie chart shows the top 10 significant enrichment terms. (B, E, and H) Bar plot shows the top 10 enrichment scores ($-\log_{10}$ (p value)). (C, F, and I) Bar plot shows the top 10 fold enrichment values ((count/pop. hits)/(list. total/pop. total)). "Count" stands for the number of DE genes associated with the listed ID of gene ontology term; "Pop.Hits" stands for the number of background population genes associated with the listed ID of gene ontology term; "List.Total" stands for the total number of DE genes; "Pop.Total" stands for the total number of background population genes. (A–C) Biological process (BP). (D–F) Cellular component (CC). (G–I) Molecular function (MF).

carcinogenesis, inflammation, and metabolism may be involved in AD pathogenesis. In addition, the upregulated and downregulated pathway maps of the pathways with the top 10 enrichment scores are shown in Figures S2–21.

qRT-PCR Validates the Microarray Data of lncRNAs and mRNAs

We confirmed the differential expression of lncRNAs and mRNAs that were identified in the microarray using qRT-PCR (Figure 7). For example, ENSMUST00000157463, and ENSMUST00000175096, ENSMUST00000083211 and NR_040673, ENSMUST00000148940, and ENSMUST00000137025 were the top three upregulated and downregulated lncRNAs, respectively, in the microarray data on the hippocampus of SAMP8 mice compared with SAMR1 mice (Table S3; Figure 2I). The qRT-PCR assay demonstrated the same expression patterns for the lncRNAs as for the microarray data (Figures 7A–7F). Similarly, we verified the expression patterns of the top three upregulated and downregulated mRNAs (upregulated, Aga, Hist1h3h, and Mrpl40; downregulated, Elovl3, Ugt2b38, and Mup9) from the differentially expressed mRNAs (Table S6; Figure 3F). The qRT-PCR results showed that the expression of Aga, Hist1h3h, and Mrpl40 was upregulated, and the expression of Elovl3, Ugt2b38, and Mup9 was downregulated, in the hippocampal samples of SAMP8 mice compared with SAMR1 mice (Figures 7G–7L). Thus, the results from the qRT-PCR assay and the microarray data analysis were consistent, supporting a strong correlation between the qRT-PCR results and the microarray data.

Analysis of lincRNAs and Their Associated Nearby Coding Genes

Because lincRNAs (long "intergenic" non-coding RNAs) play a key role in the regulation of nearby genes,[20] we next analyzed the differentially expressed lncRNAs and their associated nearby coding genes (<300 kb). We found that a total of 780 lincRNAs (the length and fold change ranged from 60 to 43,829 and 2 to 61.5, respectively) had nearby associated coding genes, of which 271 lincRNAs were upregulated and 509 lincRNAs were downregulated (Figure 8A; Table S15). Among these lincRNAs, AK158400 had the most genes nearby (Figure 8B), all of which belonged to the histone cluster 1 family, suggesting that AK158400 may regulate the nucleosome structure of the chromosomal fiber by affecting nearby genes during AD progression. Furthermore, Figure 8C shows the top 10 lincRNAs (according to the fold change) and their associated nearby genes. Among lncRNAs,

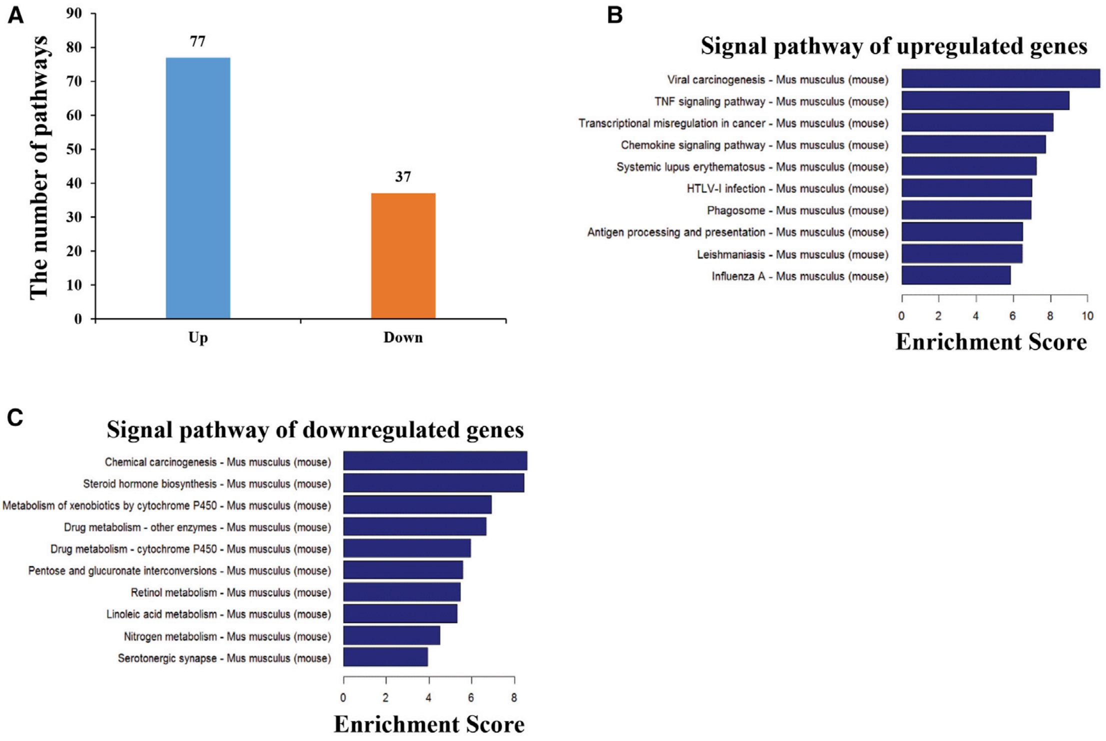

Figure 6. Pathway Analysis of mRNAs with Dysregulated Expression

(A) Number of pathways of mRNAs with dysregulated expression. Pathway analysis mapped the genes to KEGG pathways. A p value ≥ 0.05 denotes the significance correlation of the pathway to the conditions. (B and C) Pathway analysis using KEGG for the differentially expressed transcripts and schematic diagrams of the two gene categories. Pathways corresponding to the upregulated transcripts (B) and pathways corresponding to the downregulated transcripts (C) are shown. The x and y axes represent the top 10 significantly enriched pathways and their scores ($-\log_{10}$ (p value)), respectively.

antisense lncRNAs have been studied in depth, and more than 30% of annotated human transcripts have corresponding antisense lncRNAs; these lncRNAs regulate the corresponding sense mRNA at the transcriptional or post-transcriptional level through a variety of mechanisms to exert their biological functions.[21] We also focused on the differentially expressed antisense lncRNAs and their associated coding genes. We identified 97 differentially expressed human antisense lncRNAs (30 lncRNAs that were upregulated and 67 lncRNAs that were downregulated; the length and fold change ranged from 217 to 4,595 and 2 to 23, respectively) and their associated coding genes (Figures 8D and 8E; Table S16). In addition, the top 10 antisense lncRNAs and their associated coding genes are displayed in Figure 8E. All of these data will provide us with a new approach to understand AD pathogenesis in terms of AD-related lncRNAs and their associated nearby coding genes.

E230001N04Rik Regulates Its Nearby Coding Genes Srpk1 and Fkbp5 Levels and Tau Level in AD

To evaluate the mechanism of specific lncRNAs and their associated nearby coding genes in AD progression, we first detected the expression patterns of lncRNAs and their associated nearby coding genes in two AD cell models (okadaic acid and $A\beta_{42}$-induced HT22 cell models). As a result, the lncRNAs Gm26902, BC024571, Vaultrc5, Gm12260, E230001N04Rik, AK080501, and Ncr3-ps were upregulated in okadaic acid-induced HT22 cell models (Figure 9A). In $A\beta_{42}$-induced HT22 cell models, the lncRNAs Gm16581, AK020274, Tsr1, AK034693, Gm19897, and E230001N04Rik were increased compared to control group (Figure 9B). We could see that E230001N04Rik and Gm19897 were upregulated in both two AD cell models, and the expression pattern of lncRNAs was consistent with microarray data (Figures 9C and 9D). We also detected the expression of those lncRNA-associated nearby coding genes and most of them were consistent with microarray data (Figures 9C and 9D). Second, we used the coding/non-coding gene co-expression network (CNC network) to predict the target genes of 12 lncRNAs validated in two AD cell models above. 11 lncRNAs and their target gene interactions are presented in Figure S22 and Table S17. AK034693 has not shown the predicted genes because the Pearson correlation coefficient between AK034693 and coding genes was less than 0.98. To our surprise, Srpk1 and Fkbp5, the associated nearby coding genes of E230001N04Rik, were positively correlated to E230001N04Rik in the CNC network; Sirt2, an associated nearby coding gene of Gm19897, was also positive correlated to Gm19897 (Figure S22; Table S17). To evaluate the exact effect of specific lncRNAs and their associated nearby coding genes in AD, we knocked down E230001N04Rik and Gm19897 by antisense

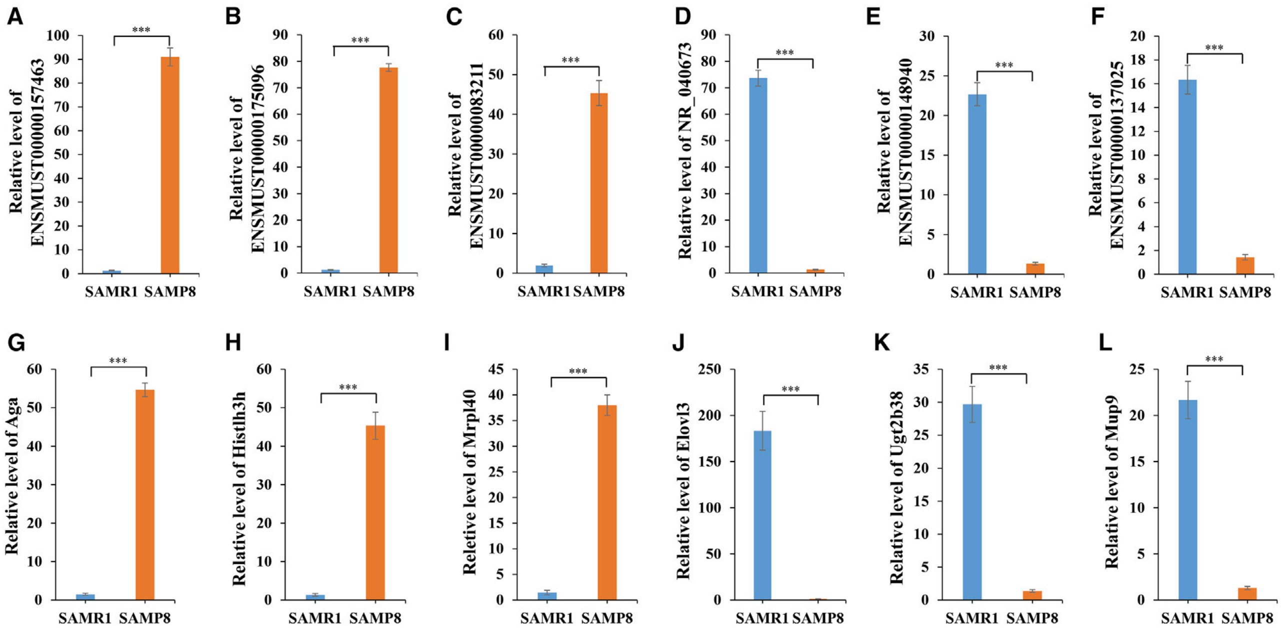

Figure 7. Validation of lncRNAs and mRNAs Using qRT-PCR

(A–F) Comparison of the expression levels of lncRNAs between the microarray and qRT-PCR results. Three differentially upregulated (A–C) and downregulated (D–F) lncRNAs were validated by qRT-PCR. ***$p < 0.001$. (G–L) Comparison of the expression levels of mRNAs between the microarray and qRT-PCR results. Three differentially upregulated (G–I) and downregulated (J–L) mRNAs were detected by qRT-PCR. The y axis represents the relative fold changes in expression across eight samples (SAMR1 = 4; SAMP8 = 4). ***$p < 0.001$.

oligonucleotides (ASOs) in AD cell models. The results showed that silencing Gm19897 did not affect the expression of nearby coding gene Sirt2 (Figure 9E), indicating that the role of Gm19897 on AD was not dependened on Sirt2. However, E230001N04Rik knockdown decreased the levels of its associated nearby coding genes Srpk1 and Fkbp5 (Figures 9F and 9G). Studies have shown that Srpk1 was increased in AD and regulated the production of tau protein, which predominantly contains four microtubule-binding repeats, which resulted in frontotemporal dementia;[22,23] Fkbp5 interactions with Hsp90 promoted neurotoxic tau accumulation and increased tau stability and polymerized microtubules,[24,25] indicating that E230001N04Rik might regulate tau protein accumulation by Srpk1 and/or Fkbp5 in AD progression. Indeed, E230001N04Rik knockdown also decreased tau level (Figure 9H). Taken together, our results show that E230001N04Rik regulated the tau level, which may occur through regulating its nearby coding genes Srpk1 and Fkbp5 in AD progression.

DISCUSSION

AD is a neurodegenerative disease characterized by devastating symptoms, such as apraxia, agnosia, aphasia, and emotional disturbance, because of progressive mental and behavioral functional decline.[26] The SAM strain consists of SAMP and SAMR strains. The SAMP8 strain is a mouse model that shows early onset of learning and memory deficits during its aging process and was phenotypically selected from the AKR/J strain by Dr. T. Takeda's laboratory at Kyoto University,[27] whereas the SAMR1 strain shows a normal aging process,[28] so it is usually used as an appropriate control for the SAMP8 strain. Many studies have shown that SAMP8 is a good model for studying age-related cognitive impairments and AD.[29] The hippocampus is widely regarded as being at the center of the brain network that supports learning and memory and is the main impaired region of AD.[3,30] Therefore, in the current study, we collected the hippocampus of SAMP8 and SAMR1 mice, but not the total brain, to comprehensively identify differentially expressed lncRNAs and their associated coding genes that may play an important role in AD pathogenesis. In the present study, we identified 3,112 differentially expressed lncRNAs and 3,191 differentially expressed mRNAs in SAMP8 mice compared to SAMR1 mice. The GO and KEGG analyses of AD-related transcripts could provide a foundation for future functional analysis. Furthermore, six lncRNAs and six mRNAs were selected for the further confirmation of the microarray results using quantitative PCR, and the results from quantitative PCR were consistent with the microarray findings. Moreover, we also analyzed lincRNAs and their nearby associated coding genes to examine the potential role of the regulation of the lincRNA-gene axis in AD pathogenesis. In addition, we also identified 97 aberrantly expressed antisense lncRNAs and their associated coding genes. All of these data will provide us with a new approach for understanding AD pathogenesis in terms of AD-related lncRNAs and their associated nearby coding genes.

Although AD-related genes and proteins have been extensively explored in recent decades, a detailed mechanism of AD is still

A

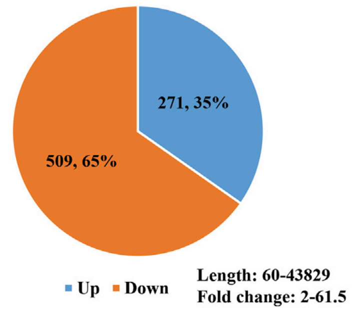

B

LincRNA with the most nearby genes

Seq Name	Gene Symbol	Genome Relationship	Nearby Gene	Nearby Gene Symbol
AK158400	AK158400	downstream	NM_013550	Hist1h3a
AK158400	AK158400	downstream	NM_030609	Hist1h1a
AK158400	AK158400	downstream	NM_175653	Hist1h3c
AK158400	AK158400	downstream	NM_175660	Hist1h2ab
AK158400	AK158400	downstream	NM_178192	Hist1h4a
AK158400	AK158400	downstream	NM_178193	Hist1h4b
AK158400	AK158400	downstream	NM_178203	Hist1h3b
AK158400	AK158400	downstream	NM_178208	Hist1h4c
AK158400	AK158400	upstream	NM_145073	Hist1h3g
AK158400	AK158400	upstream	NM_175654	Hist1h4d
AK158400	AK158400	upstream	NM_175655	Hist1h4f
AK158400	AK158400	upstream	NM_178204	Hist1h3d
AK158400	AK158400	upstream	NM_178205	Hist1h3e

C

Top 10 lincRNAs with the nearby genes

Seq Name	Gene Symbol	Fold change	Nearby Gene	Nearby Gene Symbol
ENSMUST00000083211	Vaultrc5	61.5279534	NM_009841; NM_010415; NM_028036; NM_198117; NM_001271544; NM_001271547	Cd14; Hbegf; Tmco6; Pcdha2; Slc4a9; Slc4a9
AK144830	AK144830	38.8938488	NM_001037923	Lekr1
ENSMUST00000169784	Mup-ps4	38.388219	NM_001164526; NM_001126319	Mup11; Mup9
ENSMUST00000073997	BC023105	29.9629128	NM_025776;NM_134133;NM_001034859	Rbm22; Smim3; Gm4841
ENSMUST00000098043	Mup-ps7	27.3430984	NM_001126319;NM_001164526	Mup9; Mup11
ENSMUST00000121741	Mup-ps15	23.5329797	NM_008649	Mup5
ENSMUST00000121475	Gm12399	21.6308432	ENSMUST00000178221; NM_008295; NM_001012306; NM_153193	Gm10681; Hsd3b5; Hsd3b3; Hsd3b2
AK156772	AK156772	20.5493756	NM_001122765; NM_207238	Sirt2; Fbxo27
ENSMUST00000181142	9030616G12Rik	20.2627868	NM_028873; NM_027853; NM_173733	Dnajc14; Mettl7b; Suox
ENSMUST00000120723	Mup-ps17	20.1627023	NM_008649; NM_001009550; NM_001039544	Mup5; Mup21; Mup3

D

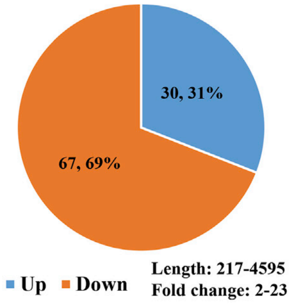

E

Top 10 antisense lncRNAs with associated genes

Seq Name	Gene Symbol	Fold change	Genome Relationship	Nearby Gene	Nearby Gene Symbol
uc029voq.1	AK161424	23.0000892	natural antisense	NM_011596	Atp6v0a2
AK040340	AK040340	20.5646377	intronic antisense	NM_019680	Elf4
AK142426	AK142426	20.2066989	natural antisense	NM_007393	Actb
ENSMUST00000125518	Gm13563	14.9376296	natural antisense	NM_001085407	Sdccag3
AK137788	AK137788	13.4127755	natural antisense	NM_008052	Dtx1
uc007nws.1	AK048798	9.9024328	natural antisense	NM_010431	Hif1a
AK143772	AK143772	8.671558	intronic antisense	NM_008290	Hsd17b2
NR_040533	Gm10804	8.0748203	intronic antisense	NM_001271430	Cd82
ENSMUST00000151220	4932702P03Rik	6.9161357	natural antisense	NM_172532	Aldh5a1
uc009ptv.1	Gm10658	6.9083969	natural antisense	NM_019651	Ptpn9

Figure 8. Analysis of lncRNAs and Their Nearby Coding Genes

(A) Pie chart shows the number of lincRNAs (the length and fold change ranged from 60 to 43,829 and 2 to 61.5, respectively) that had nearby coding genes (<300 kb). The lincRNAs with nearby coding genes (distance between the lncRNA and coding gene <300 kb) were identified. Blue indicates upregulated lincRNAs, and orange indicates downregulated lincRNAs. (B) AK158400 had the most nearby coding genes. AK158400 had 13 nearby coding genes. (C) Top 10 lincRNAs (according to fold change) with their nearby coding genes. (D) Pie chart shows the number of antisense lncRNAs (the length and fold change ranged from 217 to 4,595 and 2 to 23, respectively) with associated coding genes. Blue indicates upregulated antisense lncRNAs, and orange indicates downregulated antisense lncRNAs. (E) Top 10 antisense lncRNAs (according to fold change) with their associated coding genes.

lacking. Much is yet to be discovered about the precise biological changes that cause AD outside of genes and proteins. lncRNAs are a class of long RNAs (>200 nt)[31] that are presumed to participate in many essential biological processes[32] and human diseases, including cancer and AD.[33,34] For example, lncRNA 51A overlaps with SORL1 and could affect Aβ formation.[14,15] BC200 exhibited abnormal subcellular localization and expression levels in AD patients.[35] BACE1-AS regulates BACE1 mRNA and protein expression and enhances APP processing and $A\beta_{1-42}$.[12,13] 17A, NDM29, and NAT-Rad18 were also reported to be involved in the progression of AD.[16–18] However, with the exception of the few previously mentioned findings, our knowledge about the systematic expression profiles of lncRNAs and a comprehensive analysis of lncRNAs and their nearby coding genes in AD are still lacking. Although several studies have shown that lncRNAs are differentially expressed in 3xTg-AD[36] or APP/PS1[37] mice, a class of transgenic mice representing an uncommon familial form of AD, the roles of lncRNAs in AD remain largely unknown because none of the currently

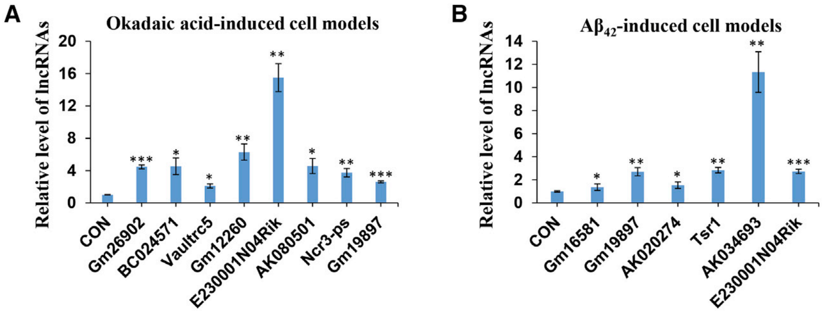

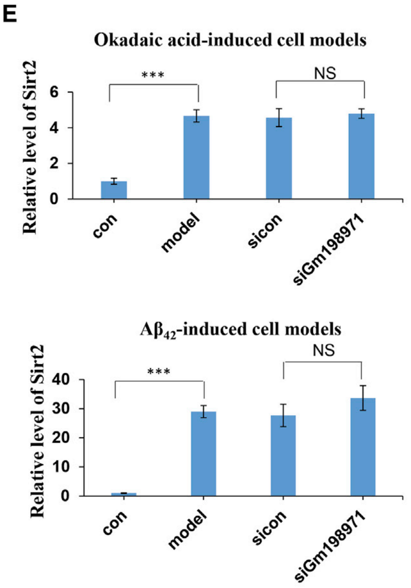

C

Okadaic acid-induced HT22 cell models

LncRNAs	Microarray data Regulation	Microarray data Fold	Validation by qPCR Regulation	Validation by qPCR Fold	Nearby genes	Microarray data Regulation	Microarray data Fold	Validation by qPCR Regulation	Validation by qPCR Fold
Gm26902	up	4.2	up	4.5	Ch25h	up	48.2	up	21
BC024571	up	12.7	up	4.5	Pdk1	down	6.3	up	1.2
BC024571	up	12.7	up	4.5	Itga6	down	4.3	up	3.6
Vaultrc5	up	61.5	up	2.1	Cd14	up	29	up	38.2
Vaultrc5	up	61.5	up	2.1	Hbegf	up	3.9	up	3.8
Gm12260	up	7.9	up	6.3	Arf1	up	3.9	up	1.4
E230001N04Rik	up	5.9	up	15.5	Srpk1	up	2.9	up	3.2
E230001N04Rik	up	5.9	up	15.5	Fkbp5	up	5	up	7.8
AK080501	up	5.5	up	4.6	Gsk3a	up	3.3	down	0.9
Ncr3-ps	up	5.4	up	3.7	Clic1	up	2.8	up	1.9
Ncr3-ps	up	5.4	up	3.7	Tnf	up	5.8	up	11
Gm19897	up	2.7	up	2.6	Sirt2	up	3.1	up	4.3

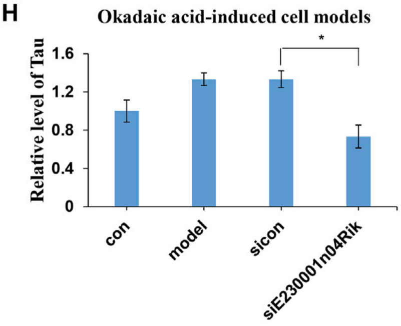

D

Aβ42-induced HT22 cell models

LncRNAs	Microarray data Regulation	Microarray data Fold	Validation by qPCR Regulation	Validation by qPCR Fold	Nearby genes	Microarray data Regulation	Microarray data Fold	Validation by qPCR Regulation	Validation by qPCR Fold
Gm16581	up	5.7	up	1.4	Coq10b	up	3.7	down	0.1
Gm19897	up	2.7	up	2.7	Sirt2	up	3.1	up	28
AK020274	up	4.8	up	1.5	Tpp1	up	2.2	up	33
AK020274	up	4.8	up	1.5	Smpd1	up	2	down	0.4
Tsr1	up	3.3	up	2.8	Srr	down	3	up	2.9
AK034693	up	2.2	up	11	Car14	down	3.2	down	0.1
E230001N04Rik	up	5.9	up	2.7	Srpk1	up	3	up	2.8
E230001N04Rik	up	5.9	up	2.7	Fkbp5	up	5	up	3.6

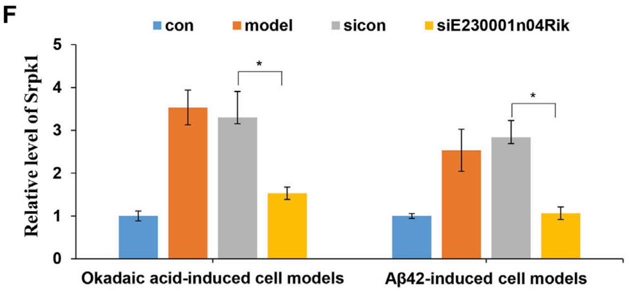

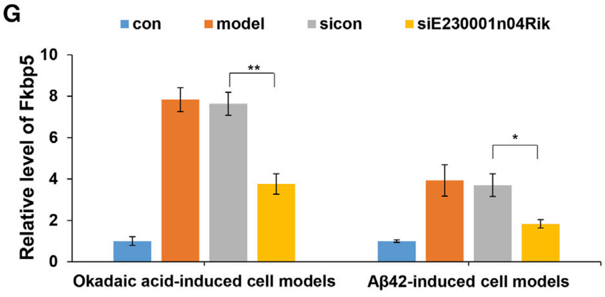

Figure 9. E230001N04Rik Regulates Its Nearby Coding Genes Srpk1 and Fkbp5 Levels and Tau Level in Alzheimer's Disease

(A and B) Expression patterns of lncRNAs in okadaic acid-induced (A) and Aβ42-induced (B) HT22 cell models. (C and D) Expression patterns of lncRNAs and their associated nearby coding genes in microarray data and two AD cell models by qRT-PCR validation. (C) Okadaic acid-induced HT22 cell models. (D) Aβ42-induced HT22 cell models. (E) The expression of Sirt2 in AD cell models by silence of Gm19897. (F and G) Expression of Srpk1 (F) and Fkbp5 (G) in AD cell models by silence of E230001N04Rik. (H) Expression of tau in okadaic acid-induced HT22 cell models by silence of E230001N04Rik. *p < 0.05, **p < 0.01, ***p < 0.001. NS, not significant.

available models recapitulates all aspects of human AD.[38] In our current study, we collected the hippocampus, the region of the brain that is considered to be mainly impaired in AD, but not the whole brain of SAMP8 and SAMR1 mice, mice that are regarded as good models for studying learning and memory, to comprehensively and systematically study lncRNAs in AD. Our results were more rigorous and accurate than those of Zhang et al.,[39] who used the total brain to analyze the lncRNA expression profiles and deficiencies during

an analysis of AD-related lncRNAs and their associated nearly coding genes. As a result, we identified 1,140 lncRNAs (the length and fold change ranged from 60 to 27,759 and 2 to 125.9, respectively) with upregulated expression, and 1,972 lncRNAs (the length and fold change ranged from 61 to 117,168 and 2 to 101.3, respectively) displayed downregulated expression, among the 3,112 differentially expressed lncRNAs in the hippocampus of SAMP8 mice compared with age-matched SAMR1 mice; these results were tremendously different from those of Zhang et al.[39] in terms of mRNA expression profiles and GO and pathway analyses. These results suggested that AD pathogenesis is completely different in different regions, even in the same animal model, and the selection of which region to study deserves careful consideration.

In addition to lncRNA expression profiles, we also analyzed the aberrant mRNA expression profiles in the hippocampus of SAMP8. A total of 1,880 mRNAs had upregulated expression, and 1,311 mRNAs displayed downregulated expression, among the 3,191 differentially expressed genes in SAMP8 mice compared with SAMR1 mice; most of the dysregulated mRNAs were in the $2 \leq$ fold change < 4 subgroup. GO analysis showed that metabolic process reprogramming was closely related to AD, which was also mentioned by Bredesen.[40] In addition, the enrichment score values of the significantly correlated pathways of the top 10 downregulated mRNAs indicated that carcinogenesis, inflammation, and metabolism may be involved in AD pathogenesis, supporting an association between AD and cancer. Indeed, many studies have shown that cancer and AD do not often occur together, and this has been known for many years.[41,42] Moreover, the results of the validation studies of lncRNAs and mRNAs by qRT-PCR were consistent with the microarray data, supporting the strong reliability of the microarray data in our study.

Another strength of the current study was the analysis of AD-related lincRNAs and their nearby coding genes. Some lincRNAs are known to play critical roles in diverse cellular processes through a variety of mechanisms.[43] Although some lincRNA loci encode RNAs that act non-locally (in *trans*)[44], there is emerging evidence that many lincRNA loci act locally (in *cis*) to regulate the expression of nearby genes.[45,46] We found that a total of 780 lincRNAs had nearby associated coding genes, including both upstream and downstream genes. Among these lincRNAs, AK158400 had the most coding genes nearby (13 genes), all of which belonged to the histone cluster 1 family, suggesting that AK158400 might regulate the nucleosome structure of the chromosomal fiber by affecting nearby genes in AD progression; however, the role of AK158400 and its nearby coding genes in AD needs to be more carefully examined. Furthermore, antisense lncRNAs have been studied in depth, and more than 30% of the annotated human transcripts have corresponding antisense lncRNAs, which regulate the corresponding sense mRNA at the transcriptional or post-transcriptional level through a variety of mechanisms to exert their biological functions.[21,47]. In the current study, we identified 97 differentially expressed antisense lncRNAs and their associated coding genes, whose role in AD still needs further study. To evaluate the mechanism of specific lncRNAs and their associated nearby coding genes in AD progression, we detected the expression patterns of lncRNAs and their associated nearby coding genes in two AD cell models. In addition, we used the CNC network to predict the target genes of lncRNAs and found that E230001N04Rik and its associated nearby coding genes Srpk1 and Fkbp5 were positive correlated. What interested us was that Srpk1 and Fkbp5 as well as tau were downregulated when knocking down E230001N04Rik by antisense oligonucleotides in AD cell models. Studies have shown that Srpk1 regulated the production of tau protein, which resulted in frontotemporal dementia;[22,23] Fkbp5 interacted with Hsp90 to promote neurotoxic tau accumulation and increased tau stability,[24,25] indicating that E230001N04Rik regulated tau protein accumulation by Srpk1 and/or Fkbp5 in AD progression.

In conclusion, in the present study, we utilized microarray technology to systematically analyze the aberrant expression profiles of lncRNAs and mRNAs in the hippocampus of SAMP8 with AD. GO and pathway analyses further facilitated our understanding of the mechanism of AD. Moreover, the identification of lncRNAs and their associated nearby coding genes may contribute to the further study for understanding of AD pathogenesis and provide a valuable resource for the diagnosis and therapy of AD in the clinic (Figure 10).

MATERIALS AND METHODS

Animal and Tissue Collection

SAMP8 mice and SAMR1 mice were purchased from Tianjin University of Traditional Chinese Medicine (Tianjin, China). The mice were housed with one mouse per cage in a standard environment (22°C ± 2°C, 45%–55% humidity, and a 12-h light/12-h dark cycle) and allowed to eat freely until they were 8 months old. Animals were randomly selected for the Morris water maze (MWM) test. We selected four animals from each group and gave them general anesthesia for the collection of hippocampal tissue. All animal protocols were carried out in strict accordance with the recommendations of the *Guide for the Care and Use of Laboratory Animals* from the National Institutes of Health. The project identification code is 20150301009, and approval date of the Ethics Committee is May 20, 2015.

MWM Test

The learning and memory abilities of 8-month-old SAMP8 mice were evaluated using the MWM test.[48,49] In brief, the objective for the mouse was to find the platform (8 cm in diameter), which was placed 1 cm below the water surface in the middle of one quadrant of the pool, 20 cm from the wall. Mice were given 1 day of adaptive training followed by trials on 5 consecutive days. Each trial started by gently placing the mouse into the water with its head toward the pool wall in any of the three quadrants without the platform. If an animal found the platform within 90 s, it was left on the platform for 30 s. If an animal did not find the platform, they were gently guided to the platform by the experimenter and left there for 30 s. On the last day of the MWM test, the platform was removed, and the number of times that the mice

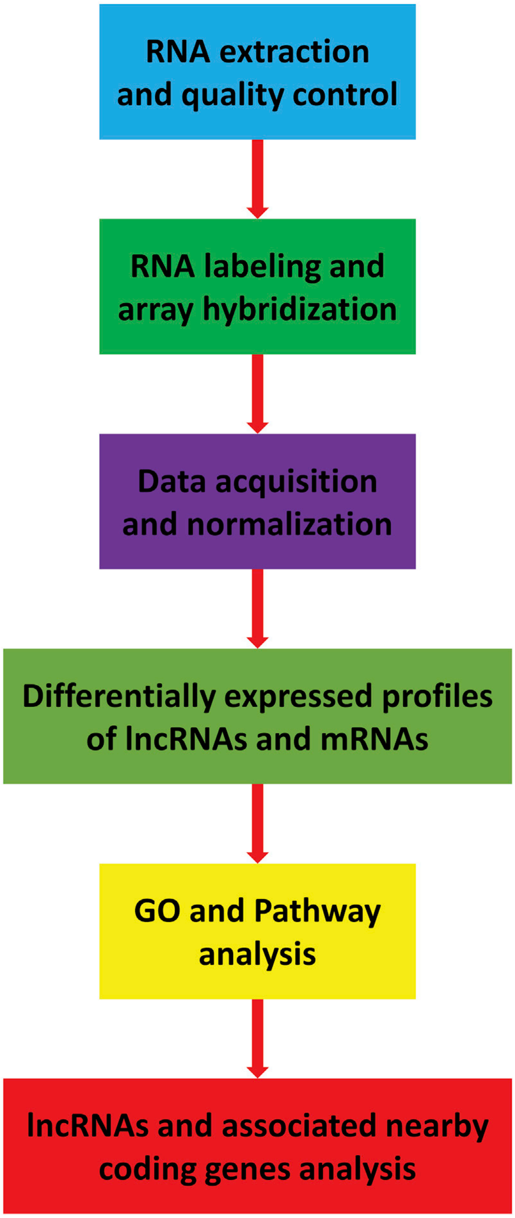

Figure 10. Flowchart of the Experiment

crossed the location of the original platform was recorded. Between trials, all mice were placed back in their home cages using a spoon-net to avoid direct contact with the experimenter. All trials were tracked automatically by a digital tracking system (Guangzhou Feidi Biological Technology) assessing path length, swimming speed, and escape latency.

Arraystar lncRNA Array

RNA Extraction

Briefly, hippocampal tissues were pulverized and homogenized using a BioPulverizer (BioSpec) and Mini-Beadbeater-16 (BioSpec), respectively. The lysed cells were directly placed in a culture dish and 1 mL of TRIzol reagent was added (Invitrogen) to isolate the RNA. RNA quantification and quality control were performed with a NanoDrop ND-1000 (Thermo Fisher Scientific) and agarose gel electrophoresis.

Microarray

An Arraystar mouse lncRNA microarray v3.0 was designed for the global profiling of mouse lncRNAs and protein-coding transcripts. Approximately 35,923 lncRNAs and 24,881 coding transcripts could be detected by our third-generation lncRNA microarray.

RNA Labeling and Array Hybridization

Sample labeling and array hybridization were performed according to the Agilent one-color microarray-based gene expression analysis protocol (Agilent Technologies) with minor modifications. Briefly, mRNA was purified from total RNA after the removal of rRNA (mRNA-ONLY eukaryotic mRNA isolation kit, Epicenter Biotechnologies). Then, each sample was amplified and transcribed into fluorescent cRNA along the entire length of the transcripts without 3′ bias, utilizing a random priming method (Arraystar flash RNA labeling kit). The labeled cRNAs were purified with an RNeasy mini kit (QIAGEN). The concentration and specific activity of the labeled cRNAs (pmol of Cy3/μg of cRNA) were measured with a NanoDrop ND-1000. One microgram of each labeled cRNA was fragmented by adding 5 μL of 10× blocking agent and 1 μL of 25× fragmentation buffer and were then heated at 60°C for 30 min. Finally, 25 μL of 2× GE Healthcare hybridization buffer was added to dilute the labeled cRNA. Fifty microliters of hybridization solution was dispensed into the gasket slide and assembled onto the lncRNA expression microarray slide. The slides were incubated for 17 h at 65°C in an Agilent hybridization oven. The hybridized arrays were washed, fixed, and scanned using an Agilent DNA microarray scanner (part no. G2505C).

lncRNA Microarray Data Analysis

The Agilent feature extraction software (version 11.0.1.1) was used to analyze the acquired array images. Quantile normalization and subsequent data processing were performed using the GeneSpring GX v12.1 software package (Agilent Technologies). In brief, quantile normalization was performed by the following steps: (1) The expression values of each sample were sorted in ascending order and placed next to each other. (2) Each column was sorted in ascending order. The mean of the sorted order across all samples was taken. Thus, each row in this sorted matrix had a value equal to the previous mean. (3) The modified matrix as obtained in the previous step was rearranged to have the same ordering as the input matrix. After quantile normalization of the raw data, lncRNAs and mRNAs that in at least four out of the eight samples were flagged as present or marginal (“all targets value”) were chosen for further data analysis. Differentially expressed lncRNAs and mRNAs that were significantly different between the two groups were identified through p value/false discovery rate (FDR) filtering. lncRNAs and mRNAs that were differentially

expressed between the two samples were identified through fold change filtering. For the multiple test correction, we used the Benjamini-Hochberg method. Hierarchical clustering and combined analysis were performed using original scripts from our laboratory.

qRT-PCR

The results of the lncRNA and mRNA expression profiles were validated by qRT-PCR. Total mRNA was extracted with TRIzol reagent (Invitrogen), and the concentration was measured with a NanoDrop 2000. Reverse transcription to generate cDNA was performed by using Takara 5× PrimeScript RT master mix. qRT-PCR was performed according to the manufacturer's instructions. The specific quantitative primers for the validation of lncRNAs and mRNAs and the detailed protocol for qRT-PCR are described in Hong et al.[50] and Supplemental Materials and Methods.

GO and KEGG Analyses

GO Analysis

The GO project provides a controlled vocabulary to describe gene and gene product attributes in any organism (http://www.geneontology.org/). GO enrichment analysis was used to identify differentially expressed genes by using topGO.[51] Fisher's exact test was used to determine whether there was more overlap between the differentially expressed gene list and the GO annotation list than would be expected by chance. The p value denotes the significance of the GO term enrichment in the differentially expressed genes. As the p value decreases, the significance of the GO term also increases ($p \leq 0.05$ is recommended).

KEGG Analysis

Pathway analysis is a functional analysis that maps genes to KEGG pathways. The p value (EASE score, Fisher p value, or hypergeometric p value) denotes the significance of correlation of the pathway to the conditions. As the p value decreases, the more the significance of the pathway correlation also increases (the recommend p value cutoff is 0.05).

lncRNA-mRNA Co-expression Network

For lncRNA-mRNA correlation analysis, we calculated the Pearson correlation of lncRNA expression value with that of each mRNA to identify significantly co-expressed lncRNAs and mRNAs with the Pearson correlation coefficient ≥ 0.98. A CNC network was constructed based on the positive or negative correlation analysis between differentially expressed lncRNAs and mRNAs by using the open-source bioinformatics software Cytoscape.

AD Cell Models and Knockdown of lncRNAs by antisense oligonucleotide (ASO)

For the okadaic acid-induced HT22 cell model, HT22 cells were cultured and treated with 30 μM okadaic acid for 36 h. For the Aβ42-induced HT22 cell model, HT22 cells were treated with 20 μM Aβ42 for 24 h. For the lncRNA knockdown assay, HT22 cells were transfected with antisense oligonucleotides (purchased from RiboBio, Guangzhou, China) for 24 h and then the RNA was collected for qRT-PCR.

Statistical Analysis

The statistical significance was analyzed using SPSS (version 22, IBM, Armonk, NY, USA). All data are shown as the means ± SEM. $p < 0.05$ was considered significant. Briefly, a Student's t test was used to compare the qRT-PCR results. Behavioral data from the training period were first assessed for normality and sphericity using the Shapiro-Wilks test and Mauchly's test, respectively, and were then analyzed using repeated-measures ANOVA. Statistical significance was set at $p < 0.05$.

SUPPLEMENTAL INFORMATION

Supplemental Information can be found online at https://doi.org/10.1016/j.omtn.2020.02.008.

AUTHOR CONTRIBUTIONS

Shuhuan Fang, Qi Wang and Jiansong Fang conceived and designed the project. Honghai Hong, Yousheng Mo and Dongli Li performed and conducted the experiments. Honghai Hong wrote the manuscript. Zhiheng Xu, Yanfang Liao, Ping Yin, Xinning Liu and Yong Xia contributed to the writing of the manuscript.

CONFLICTS OF INTEREST

The authors declare no competing interests.

ACKNOWLEDGMENTS

This study was supported by the National Nature Science Foundation of China (grants 81403144, 81473740, 81902693, and 81673627); Guangzhou Science Technology and Innovation Commission Technology Research Projects, Open Tending Project for Construction of High-Level University, Guangzhou University of Chinese Medicine (34 (2017) and 118 (2017)); Characteristic Key Discipline Construction Fund of Chinese Internal Medicine of Guangzhou University of Chinese Medicine, Guangzhou Science Technology and Innovation Commission Technology Research Projects (201805010005), Guangzhou Municipal Health Bureau (grants 20171A011312 and 20182A011020), Guangzhou traditional Chinese medicine, integrated traditional Chinese and Western medicine science and technology project; Research project on elite talent training of Third Affiliated Hospital of Guangzhou Medical University; Foundation for Characteristic Innovation of Educational Commission of Guangdong (grant 2016KTSCX011); Open Tending Project for Construction of High-Level University, Guangzhou University of Chinese Medicine; Natural Science Foundation of Guangdong Province (2018A030310298) and Educational Commission of Guangdong Province (2017KTSCX155).

REFERENCES

1. Wu, W., Liang, X., Xie, G., Chen, L., Liu, W., Luo, G., Zhang, P., Yu, L., Zheng, X., Ji, H., et al. (2018). Synthesis and evaluation of novel ligustrazine derivatives as

multi-targeted inhibitors for the treatment of Alzheimer's disease. Molecules *23*, E2540.

2. Yaari, R., and Corey-Bloom, J. (2007). Alzheimer's disease. Semin. Neurol. *27*, 32–41.
3. Jaroudi, W., Garami, J., Garrido, S., Hornberger, M., Keri, S., and Moustafa, A.A. (2017). Factors underlying cognitive decline in old age and Alzheimer's disease: the role of the hippocampus. Rev. Neurosci. *28*, 705–714.
4. Anand, R., Gill, K.D., and Mahdi, A.A. (2014). Therapeutics of Alzheimer's disease: past, present and future. Neuropharmacology *76*, 27–50.
5. Zhang, C., Du, Q.Y., Chen, L.D., Wu, W.H., Liao, S.Y., Yu, L.H., and Liang, X.T. (2016). Design, synthesis and evaluation of novel tacrine-multialkoxybenzene hybrids as multi-targeted compounds against Alzheimer's disease. Eur. J. Med. Chem. *116*, 200–209.
6. Knauss, J.L., and Sun, T. (2013). Regulatory mechanisms of long noncoding RNAs in vertebrate central nervous system development and function. Neuroscience *235*, 200–214.
7. Tang, J., Yu, Y., and Yang, W. (2017). Long noncoding RNA and its contribution to autism spectrum disorders. CNS Neurosci. Ther. *23*, 645–656.
8. Khorkova, O., Hsiao, J., and Wahlestedt, C. (2015). Basic biology and therapeutic implications of lncRNA. Adv. Drug Deliv. Rev. *87*, 15–24.
9. Ponting, C.P., Oliver, P.L., and Reik, W. (2009). Evolution and functions of long noncoding RNAs. Cell *136*, 629–641.
10. Tan, L., Yu, J.T., Hu, N., and Tan, L. (2013). Non-coding RNAs in Alzheimer's disease. Mol. Neurobiol. *47*, 382–393.
11. Wu, P., Zuo, X., Deng, H., Liu, X., Liu, L., and Ji, A. (2013). Roles of long noncoding RNAs in brain development, functional diversification and neurodegenerative diseases. Brain Res. Bull. *97*, 69–80.
12. Faghihi, M.A., Modarresi, F., Khalil, A.M., Wood, D.E., Sahagan, B.G., Morgan, T.E., Finch, C.E., St Laurent, G., 3rd, Kenny, P.J., and Wahlestedt, C. (2008). Expression of a noncoding RNA is elevated in Alzheimer's disease and drives rapid feed-forward regulation of β-secretase. Nat. Med. *14*, 723–730.
13. Liu, T., Huang, Y., Chen, J., Chi, H., Yu, Z., Wang, J., and Chen, C. (2014). Attenuated ability of BACE1 to cleave the amyloid precursor protein via silencing long noncoding RNA BACE1-AS expression. Mol. Med. Rep. *10*, 1275–1281.
14. Ciarlo, E., Massone, S., Penna, I., Nizzari, M., Gigoni, A., Dieci, G., Russo, C., Florio, T., Cancedda, R., and Pagano, A. (2013). An intronic ncRNA-dependent regulation of SORL1 expression affecting Aβ formation is upregulated in post-mortem Alzheimer's disease brain samples. Dis. Model. Mech. *6*, 424–433.
15. Ma, Q.L., Galasko, D.R., Ringman, J.M., Vinters, H.V., Edland, S.D., Pomakian, J., Ubeda, O.J., Rosario, E.R., Teter, B., Frautschy, S.A., and Cole, G.M. (2009). Reduction of SorLA/LR11, a sorting protein limiting β-amyloid production, in Alzheimer disease cerebrospinal fluid. Arch. Neurol. *66*, 448–457.
16. Massone, S., Vassallo, I., Fiorino, G., Castelnuovo, M., Barbieri, F., Borghi, R., Tabaton, M., Robello, M., Gatta, E., Russo, C., et al. (2011). 17A, a novel non-coding RNA, regulates GABA B alternative splicing and signaling in response to inflammatory stimuli and in Alzheimer disease. Neurobiol. Dis. *41*, 308–317.
17. Massone, S., Ciarlo, E., Vella, S., Nizzari, M., Florio, T., Russo, C., Cancedda, R., and Pagano, A. (2012). NDM29, a RNA polymerase III-dependent non coding RNA, promotes amyloidogenic processing of APP and amyloid β secretion. Biochim. Biophys. Acta *1823*, 1170–1177.
18. Parenti, R., Paratore, S., Torrisi, A., and Cavallaro, S. (2007). A natural antisense transcript against Rad18, specifically expressed in neurons and upregulated during β-amyloid-induced apoptosis. Eur. J. Neurosci. *26*, 2444–2457.
19. Huang, M., Qi, W., Fang, S., Jiang, P., Yang, C., Mo, Y., Dong, C., Li, Y., Zhong, J., Cai, W., et al. (2018). Pigment epithelium-derived factor plays a role in Alzheimer's disease by negatively regulating Aβ42. Neurotherapeutics *15*, 728–741.
20. Ebisuya, M., Yamamoto, T., Nakajima, M., and Nishida, E. (2008). Ripples from neighbouring transcription. Nat. Cell Biol. *10*, 1106–1113.
21. Faghihi, M.A., and Wahlestedt, C. (2009). Regulatory roles of natural antisense transcripts. Nat. Rev. Mol. Cell Biol. *10*, 637–643.
22. Hartmann, A.M., Rujescu, D., Giannakouros, T., Nikolakaki, E., Goedert, M., Mandelkow, E.M., Gao, Q.S., Andreadis, A., and Stamm, S. (2001). Regulation of alternative splicing of human tau exon 10 by phosphorylation of splicing factors. Mol. Cell. Neurosci. *18*, 80–90.
23. Daniilidou, M., Tsolaki, M., Giannakouros, T., and Nikolakaki, E. (2011). Detection of elevated antibodies against SR protein kinase 1 in the serum of Alzheimer's disease patients. J. Neuroimmunol. *238*, 67–72.
24. Blair, L.J., Nordhues, B.A., Hill, S.E., Scaglione, K.M., O'Leary, J.C., 3rd, Fontaine, S.N., Breydo, L., Zhang, B., Li, P., Wang, L., et al. (2013). Accelerated neurodegeneration through chaperone-mediated oligomerization of tau. J. Clin. Invest. *123*, 4158–4169.
25. Jinwal, U.K., Koren, J., 3rd, Borysov, S.I., Schmid, A.B., Abisambra, J.F., Blair, L.J., Johnson, A.G., Jones, J.R., Shults, C.L., O'Leary, J.C., 3rd, et al. (2010). The Hsp90 cochaperone, FKBP51, increases tau stability and polymerizes microtubules. J. Neurosci. *30*, 591–599.
26. Hara, Y., McKeehan, N., and Fillit, H.M. (2019). Translating the biology of aging into novel therapeutics for Alzheimer disease. Neurology *92*, 84–93.
27. Miyamoto, M. (1997). Characteristics of age-related behavioral changes in senescence-accelerated mouse SAMP8 and SAMP10. Exp. Gerontol. *32*, 139–148.
28. Takeda, T. (1999). Senescence-accelerated mouse (SAM): a biogerontological resource in aging research. Neurobiol. Aging *20*, 105–110.
29. Poon, H.F., Farr, S.A., Thongboonkerd, V., Lynn, B.C., Banks, W.A., Morley, J.E., Klein, J.B., and Butterfield, D.A. (2005). Proteomic analysis of specific brain proteins in aged SAMP8 mice treated with alpha-lipoic acid: implications for aging and age-related neurodegenerative disorders. Neurochem. Int. *46*, 159–168.
30. Bannerman, D.M., Sprengel, R., Sanderson, D.J., McHugh, S.B., Rawlins, J.N., Monyer, H., and Seeburg, P.H. (2014). Hippocampal synaptic plasticity, spatial memory and anxiety. Nat. Rev. Neurosci. *15*, 181–192.
31. Mercer, T.R., Dinger, M.E., and Mattick, J.S. (2009). Long non-coding RNAs: insights into functions. Nat. Rev. Genet. *10*, 155–159.
32. Shen, J., Liu, L., Zhang, F., Gu, J., and Pan, G. (2019). lncRNA TapSAKI promotes inflammation injury in HK-2 cells and urine derived sepsis-induced kidney injury. J. Pharm. Pharmacol. *71*, 839–848.
33. Batista, P.J., and Chang, H.Y. (2013). Long noncoding RNAs: cellular address codes in development and disease. Cell *152*, 1298–1307.
34. Wang, L.K., Chen, X.F., He, D.D., Li, Y., and Fu, J. (2017). Dissection of functional lncRNAs in Alzheimer's disease by construction and analysis of lncRNA-mRNA networks based on competitive endogenous RNAs. Biochem. Biophys. Res. Commun. *485*, 569–576.
35. Mus, E., Hof, P.R., and Tiedge, H. (2007). Dendritic BC200 RNA in aging and in Alzheimer's disease. Proc. Natl. Acad. Sci. USA *104*, 10679–10684.
36. Lee, D.Y., Moon, J., Lee, S.T., Jung, K.H., Park, D.K., Yoo, J.S., Sunwoo, J.S., Byun, J.I., Shin, J.W., Jeon, D., et al. (2015). Distinct expression of long non-coding RNAs in an Alzheimer's disease model. J. Alzheimers Dis. *45*, 837–849.
37. Fang, M., Zhang, P., Zhao, Y., and Liu, X. (2017). Bioinformatics and co-expression network analysis of differentially expressed lncRNAs and mRNAs in hippocampus of APP/PS1 transgenic mice with Alzheimer disease. Am. J. Transl. Res. *9*, 1381–1391.
38. Van Dam, D., and De Deyn, P.P. (2011). Animal models in the drug discovery pipeline for Alzheimer's disease. Br. J. Pharmacol. *164*, 1285–1300.
39. Zhang, S., Qin, C., Cao, G., Xin, W., Feng, C., and Zhang, W. (2016). Systematic analysis of long noncoding RNAs in the senescence-accelerated mouse prone 8 brain using RNA sequencing. Mol. Ther. Nucleic Acids *5*, e343.
40. Bredesen, D.E. (2015). Metabolic profiling distinguishes three subtypes of Alzheimer's disease. Aging (Albany N.Y.) *7*, 595–600.
41. Nixon, D.W. (2017). The inverse relationship between cancer and Alzheimer's disease: a possible mechanism. Curr. Alzheimer Res. *14*, 883–893.
42. Shafi, O. (2016). Inverse relationship between Alzheimer's disease and cancer, and other factors contributing to Alzheimer's disease: a systematic review. BMC Neurol. *16*, 236.

43. Guttman, M., Amit, I., Garber, M., French, C., Lin, M.F., Feldser, D., Huarte, M., Zuk, O., Carey, B.W., Cassady, J.P., et al. (2009). Chromatin signature reveals over a thousand highly conserved large non-coding RNAs in mammals. Nature *458*, 223–227.

44. Kretz, M., Siprashvili, Z., Chu, C., Webster, D.E., Zehnder, A., Qu, K., Lee, C.S., Flockhart, R.J., Groff, A.F., Chow, J., et al. (2013). Control of somatic tissue differentiation by the long non-coding RNA TINCR. Nature *493*, 231–235.

45. Engreitz, J.M., Haines, J.E., Perez, E.M., Munson, G., Chen, J., Kane, M., McDonel, P.E., Guttman, M., and Lander, E.S. (2016). Local regulation of gene expression by lncRNA promoters, transcription and splicing. Nature *539*, 452–455.

46. Paralkar, V.R., Taborda, C.C., Huang, P., Yao, Y., Kossenkov, A.V., Prasad, R., Luan, J., Davies, J.O., Hughes, J.R., Hardison, R.C., et al. (2016). Unlinking an lncRNA from its associated *cis* element. Mol. Cell *62*, 104–110.

47. Vadaie, N., and Morris, K.V. (2013). Long antisense non-coding RNAs and the epigenetic regulation of gene expression. Biomol. Concepts *4*, 411–415.

48. Hunter, A.J., and Roberts, F.F. (1988). The effect of pirenzepine on spatial learning in the Morris water maze. Pharmacol. Biochem. Behav. *30*, 519–523.

49. Vorhees, C.V., and Williams, M.T. (2006). Morris water maze: procedures for assessing spatial and related forms of learning and memory. Nat. Protoc. *1*, 848–858.

50. Hong, H., Zeng, Y., Jian, W., Li, L., Lin, L., Mo, Y., Liu, M., Fang, S., and Xia, Y. (2018). CDK7 inhibition suppresses rheumatoid arthritis inflammation via blockage of NF-κB activation and IL-1β/IL-6 secretion. J. Cell. Mol. Med. *22*, 1292–1301.

51. Zou, R., Zhang, D., Lv, L., Shi, W., Song, Z., Yi, B., Lai, B., Chen, Q., Yang, S., and Hua, P. (2019). Bioinformatic gene analysis for potential biomarkers and therapeutic targets of atrial fibrillation-related stroke. J. Transl. Med. *17*, 45.

OMTN, Volume *20*

Supplemental Information

Aberrant Expression Profiles of lncRNAs and Their Associated Nearby Coding Genes in the Hippocampus of the SAMP8 Mouse Model with AD

Honghai Hong, Yousheng Mo, Dongli Li, Zhiheng Xu, Yanfang Liao, Ping Yin, Xinning Liu, Yong Xia, Jiansong Fang, Qi Wang, and Shuhuan Fang

Supplemental information

Materials and methods

qRT-PCR

Total mRNA was extracted by TRIzol Reagent (Invitrogen life technologies) and the concentration was measure by nanodrop2000 and reversed transcription to cDNA by Takara 5×Primer Script RT Master Mix. qRT-PCR was performed by Bio-rad CFX384 and TOYOBO 2×SYBR is used. The expression of lncRNAs and mRNAs were measured by the Bio-rad CFX manager and GAPDH was used as reference. Primer sequences were described in table 1. Normalized Fold Expression: $\Delta\Delta CQ = 2^{(-\Delta CQ)}$, $\Delta CQ = CQ_{target} - CQ_{GAPDH}$

Table1. Primer sequence for lncRNAs and mRNAs

Seq Name	Gene Symbol	Forward primer	Reverse primer
ENSMUST00000157463	Rmrp	5'GCTCTGAAGGCCTGTTTCCT-3'	5'GACTTGCGGGGGAAGTCTAC-3'
ENSMUST00000175096	Rpph1	5'CGGAGCTTGGAACAGACTCA-3'	5'GAATTGGGTTATGAGGCCCG-3'
ENSMUST00000083211	Vaultrc5	5'GCTCAGCGGTTACTTCGACA-3'	5'CGGGTTAGGTAAGTGGTTGGT-3'
NR_040673	Gm12718	5'TCAAGGGTGCAGTCCATCAC-3'	5'TGCACAATCACCCAGGAGTC-3'
ENSMUST00000148940	Mup2	5'ACCTATCCAATGCCAATCGCT-3'	5'GATATTGCCCACCTGGAGGC-3'
ENSMUST00000137025	Mup9	5'AGACGTGGTCCTGACAGACA-3'	5'AGGGTCAGTCCCAAACACAG-3'
NM_001005847	Aga	5'-CCTATGCTGATGACACGGCT-3'	5'-CTACAGCTTGGTAGCTCGGC-3'
NM_178206	Hist1h3h	5'-TTGGAAGCTCGGGTGTTACC-3'	5'-GTAGCGGTGAGGCTTCTTCA-3'
NM_010922	Mrpl40	5'-AAATCAAGACAGCGACCTCA-3'	5'-GCTGGGCTTCTAGCATGGAT-3'
NM_007703	Elovl3	5'-TGCCATCTACACGGATGACG-3'	5'-CCGTGTCTCCCAGTTCAACAA-3'
NM_133894	Ugt2b38	5'ATATTTGGGAAGGCCCACCA-3'	5'AGCAGGTTTGCAGTGGAGTC-3'
NM_001126319	Mup9	5'GGCTGGTGAATATTCTGTGACG-3'	5'GTTCTCGGCCATAGAGCCC-3'

Supplemental Table

Table S1. All Comparison LncRNAs

Table S2. LncRNA Expression Profiling Data

Table S3. Differentially Expressed LncRNAs

Table S4. All Comparison mRNAs

Table S5. mRNA Expression Profiling Data

Table S6. Differentially Expressed mRNAs

Table S7. Upregulated_BP_result(Mouse)

Table S8. Upregulated_CC_result(Mouse)

Table S9. Upregulated_MF_result(Mouse)

Table S10. Downreulated_BP_result(Mouse)

Table S11. Downreulated_CC_result(Mouse)

Table S12. Downreulated_MF_result(Mouse)

Table S13. Upregulated_Pathway Result

Table S14. Downregulated_Pathway Result

Table S15. LincRNAs and their nearby coding gene data

Table S16. Antisense lncRNAs and their associated coding gene data

Table S17. CNC network pairs

Supplemental figure legends

Figure S1. The number of significantly dysregulated lncRNAs in different databases

(A and B) The number of up- (A) and downregulated expression of lncRNAs in authoritative databases. The lncRNAs are carefully collected from the most authoritative databases such as such as RefSeq, UCSC_knowngene, Ensembl, UCR, lincRNA and many related literatures.

Figure S2-21. The top ten enrichment score pathway maps of up- and downregulated mRNAs

(S2-21) pathway maps of Viral carcinogenesis (S2), TNF signaling pathway (S3), Transcriptional misregulation in cancer (S4), Chemokine signaling pathway (S5), Systemic lupus erythematosus (S6), HTLV-I infection (S7), Phagosome (S8), Antigen processing and presentation (S9), Leishmaniasis (S10), Influenza A (S11), Chemical carcinogenesis (S12), Steroid hormone biosynthesis (S13), Metabolism of xenobiotics by cytochrome P450 (S14), Drug metabolism-other enzymes (S15), Drug metabolism-cytochrome P450 (S16), Pentose and glucuronate interconversions (S17), Retinol metabolism (S18), Linoleic acid metabolism (S19), Nitrogen metabolism (S20) and Serotonergic synapse (S21).Yellow marked nodes are associated with down-regulated genes, orange marked nodes are associated with up-regulated or only whole dataset genes, green nodes have no significance.

Figure S22. CNC network

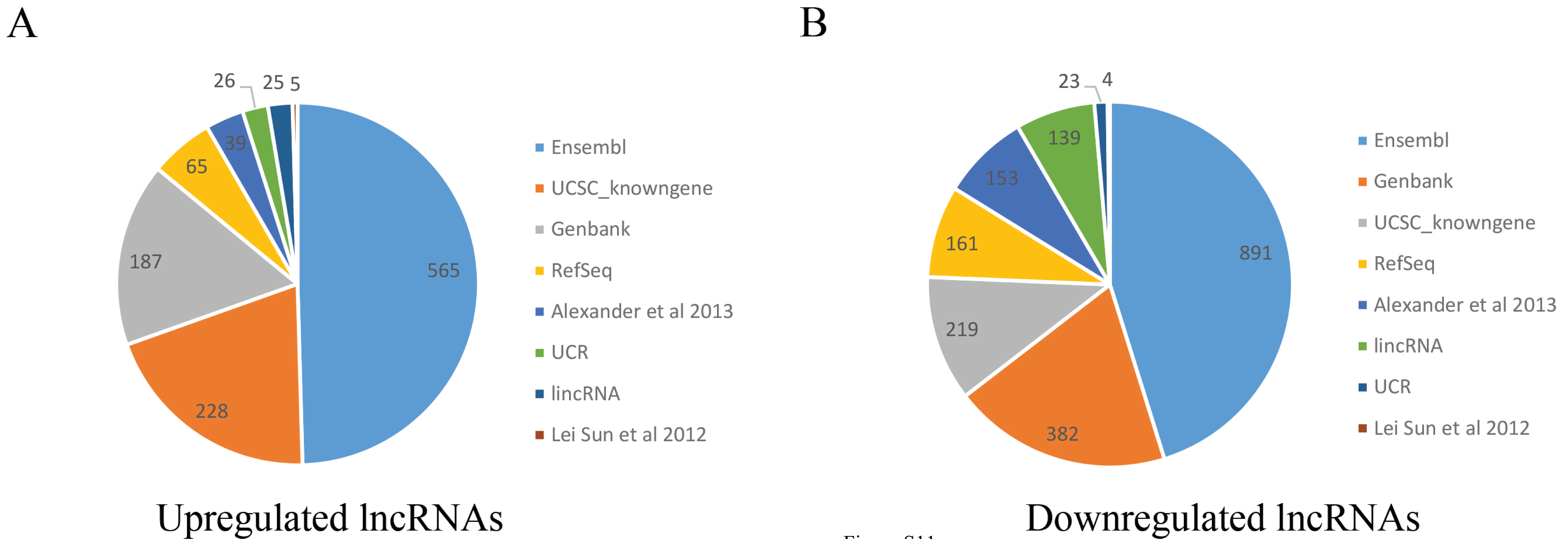
A
26
25
5
39
65
187
565
228
Ensembl
UCSC_knowngene
Genbank
RefSeq
Alexander et al 2013
UCR
lincRNA
Lei Sun et al 2012
Upregulated lncRNAs
B
23
4
139
153
161
891
219
382
Ensembl
Genbank
UCSC_knowngene
RefSeq
Alexander et al 2013
lincRNA
UCR
Lei Sun et al 2012
Downregulated lncRNAs

Figure S11

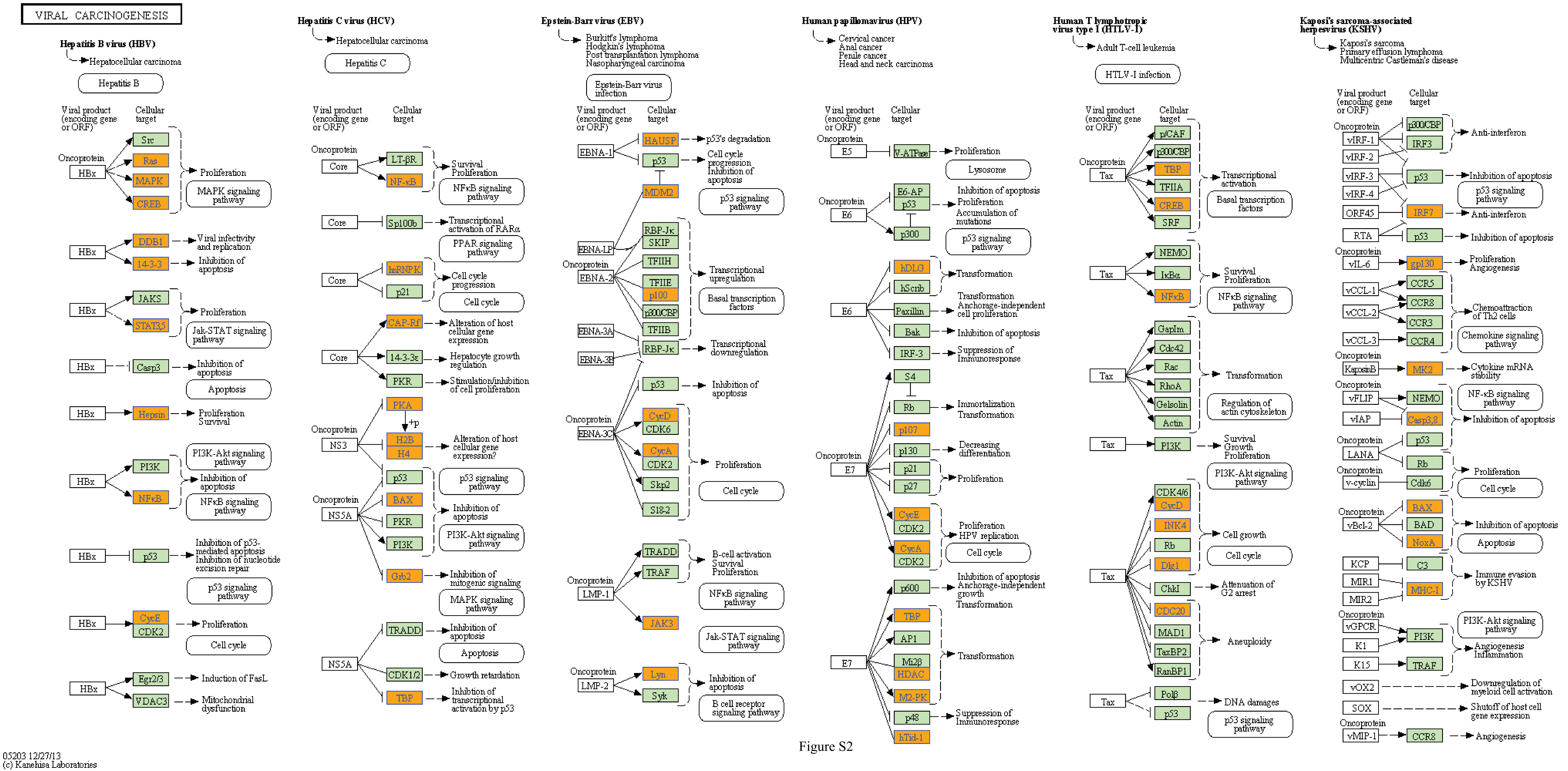
VIRAL CARCINOGENESIS
Hepatitis B virus (HBV)
Hepatitis C virus (HCV)
Epstein-Barr virus (EBV)
Human papillomavirus (HPV)
Human T lymphotropic virus type 1 (HTLV-1)
Kaposi's sarcoma-associated herpesvirus (KSHV)
Hepatocellular carcinoma
Burkitt's lymphoma
Hodgkin's lymphoma
Post transplantation lymphoma
Nasopharyngeal carcinoma
Cervical cancer
Anal cancer
Penile cancer
Head and neck carcinoma
Adult T-cell leukemia
Kaposi's sarcoma
Primary effusion lymphoma
Multicentric Castleman's disease
05203 12/27/13
(c) Kanehisa Laboratories

Figure S2

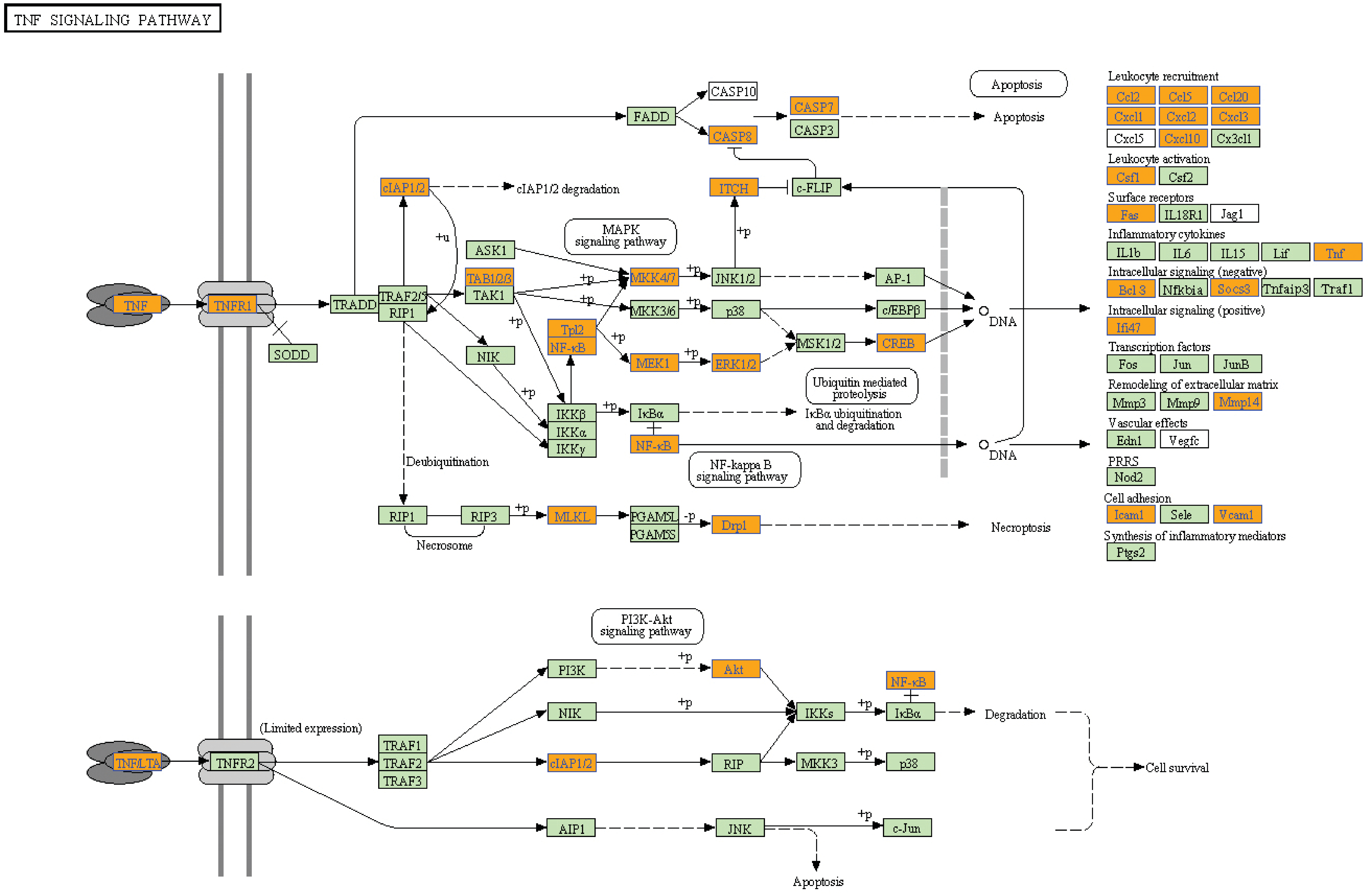

04668 5/27/13

(c) Kanehisa Laboratories

Figure S33

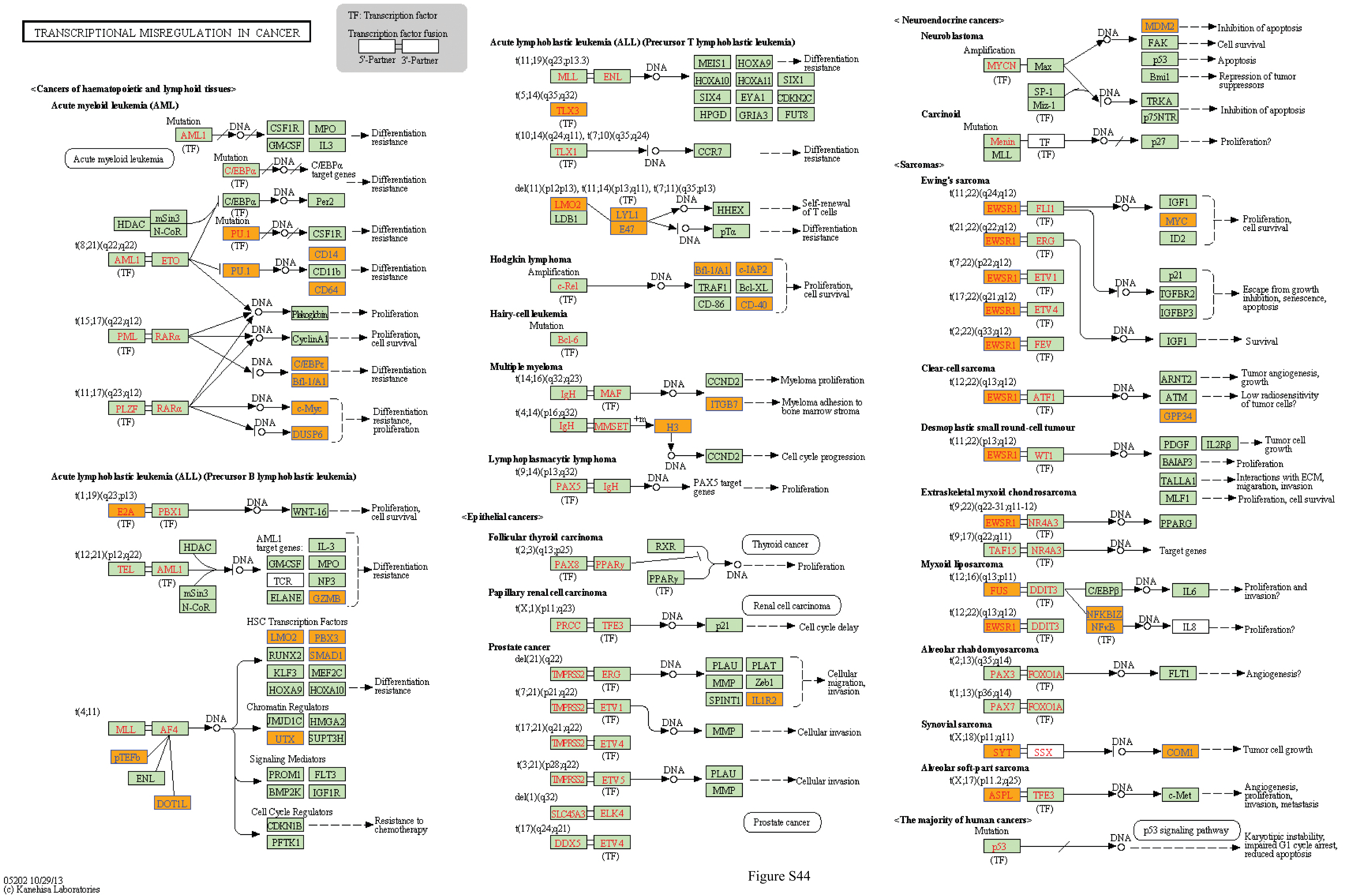
TRANSCRIPTIONAL MISREGULATION IN CANCER
TF: Transcription factor
Transcription factor fusion
5'-Partner 3'-Partner
<Cancers of haematopoietic and lymphoid tissues>
Acute myeloid leukemia (AML)
Mutation
AML1
(TF)
DNA
CSF1R
MPO
GM-CSF
IL3
Differentiation resistance
Acute myeloid leukemia
C/EBPα
C/EBPα target genes
HDAC
mSin3
N-CoR
Per2
PU.1
t(8;21)(q22;q22)
ETO
CD14
CD11b
CD64
Plakoglobin
Proliferation
t(15;17)(q22;q12)
PML
RARα
CyclinA1
Proliferation, cell survival
C/EBPε
Bfl-1/A1
t(11;17)(q23;q12)
PLZF
c-Myc
DUSP6
Differentiation resistance, proliferation
Acute lymphoblastic leukemia (ALL) (Precursor B lymphoblastic leukemia)
t(1;19)(q23;p13)
E2A
PBX1
WNT-16
t(12;21)(p12;q22)
TEL
AML1 target genes:
IL-3
TCR
NP3
ELANE
GZMB
HSC Transcription Factors
LMO2
PBX3
RUNX2
SMAD1
KLF3
MEF2C
HOXA9
HOXA10
Chromatin Regulators
JMJD1C
HMGA2
UTX
SUPT3H
t(4;11)
MLL
AF4
pTEFb
ENL
DOT1L
Signaling Mediators
PROM1
FLT3
BMP2K
IGF1R
Cell Cycle Regulators
CDKN1B
PFTK1
Resistance to chemotherapy
Acute lymphoblastic leukemia (ALL) (Precursor T lymphoblastic leukemia)
t(11;19)(q23;p13.3)
MEIS1
HOXA11
SIX1
SIX4
EYA1
CDKN2C
HPGD
GRIA3
FUT8
t(5;14)(q35;q32)
TLX3
t(10;14)(q24;q11), t(7;10)(q35;q24)
TLX1
CCR7
del(11)(p12p13), t(11;14)(p13;q11), t(7;11)(q35;p13)
LMO2
LDB1
LYL1
E47
HHEX
Self-renewal of T cells
pTα
Hodgkin lymphoma
Amplification
c-Rel
Bfl-1/A1
c-IAP2
TRAF1
Bcl-XL
CD-86
CD-40
Hairy-cell leukemia
Bcl-6
Multiple myeloma
t(14;16)(q32;q23)
IgH
MAF
CCND2
Myeloma proliferation
ITGB7
Myeloma adhesion to bone marrow stroma
t(4;14)(p16;q32)
MMSET
+m
H3
Cell cycle progression
Lymphoplasmacytic lymphoma
t(9;14)(p13;q32)
PAX5
PAX5 target genes
<Epithelial cancers>
Follicular thyroid carcinoma
t(2;3)(q13;p25)
PAX8
PPARγ
RXR
Thyroid cancer
Papillary renal cell carcinoma
t(X;1)(p11;q23)
PRCC
TFE3
p21
Cell cycle delay
Renal cell carcinoma
Prostate cancer
del(21)(q22)
TMPRSS2
ERG
PLAU
PLAT
MMP
Zeb1
SPINT1
IL1R2
Cellular migration, invasion
t(7;21)(p21;q22)
ETV1
Cellular invasion
t(17;21)(q21;q22)
ETV4
t(3;21)(p28;q22)
ETV5
del(1)(q32)
SLC45A3
ELK4
t(17)(q24;q21)
DDX5
< Neuroendocrine cancers>
Neuroblastoma
Amplification
MYCN
Max
MDM2
Inhibition of apoptosis
FAK
Cell survival
p53
Apoptosis
Bmi1
Repression of tumor suppressors
SP-1
Miz-1
TRKA
p75NTR
Carcinoid
Menin
MLL
p27
Proliferation?
<Sarcomas>
Ewing's sarcoma
t(11;22)(q24;q12)
EWSR1
FLI1
IGF1
MYC
ID2
t(21;22)(q22;q12)
t(7;22)(p22;q12)
ETV1
p21
TGFBR2
IGFBP3
Escape from growth inhibition, senescence, apoptosis
t(17;22)(q21;q12)
t(2;22)(q33;q12)
FEV
Survival
Clear-cell sarcoma
t(12;22)(q13;q12)
ATF1
ARNT2
Tumor angiogenesis, growth
ATM
Low radiosensitivity of tumor cells?
GPP34
Desmoplastic small round-cell tumour
t(11;22)(p13;q12)
WT1
PDGF
IL2Rβ
Tumor cell growth
BAIAP3
TALLA1
Interactions with ECM, migration, invasion
MLF1
Proliferation, cell survival
Extraskeletal myxoid chondrosarcoma
t(9;22)(q22-31;q11-12)
NR4A3
PPARG
t(9;17)(q22;q11)
TAF15
Target genes
Myxoid liposarcoma
t(12;16)(q13;p11)
FUS
DDIT3
C/EBPβ
IL6
Proliferation and invasion?
NFKBIZ
NFκB
IL8
Alveolar rhabdomyosarcoma
t(2;13)(q35;q14)
PAX3
FOXO1A
FLT1
Angiogenesis?
t(1;13)(p36;q14)
PAX7
Synovial sarcoma
t(X;18)(p11;q11)
SYT
SSX
COM1
Alveolar soft-part sarcoma
t(X;17)(p11.2;q25)
ASPL
c-Met
Angiogenesis, proliferation, invasion, metastasis
<The majority of human cancers>
p53 signaling pathway
Karyotipic instability, impaired G1 cycle arrest, reduced apoptosis
05202 10/29/13
(c) Kanehisa Laboratories

Figure S44

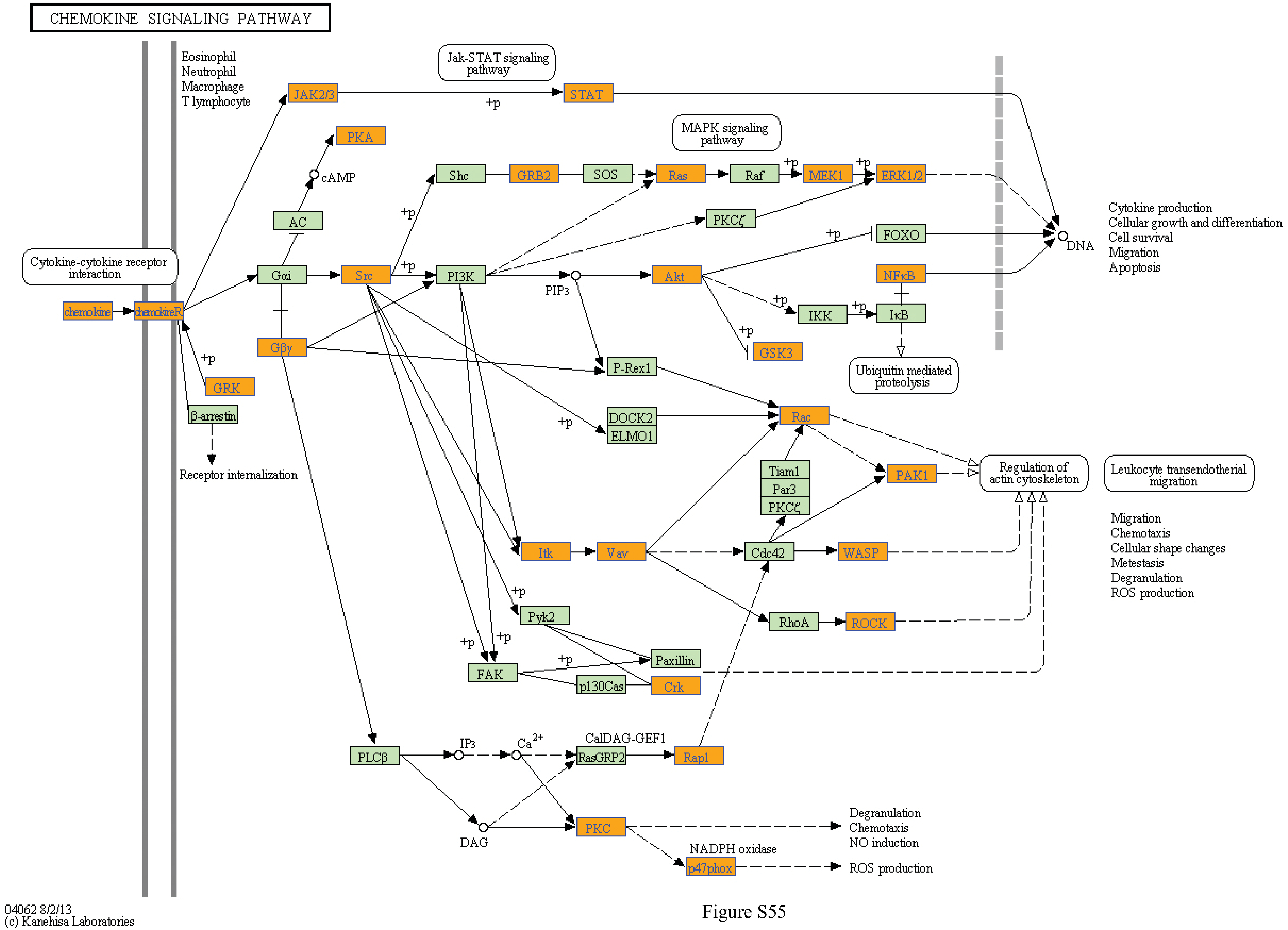
CHEMOKINE SIGNALING PATHWAY
Eosinophil
Neutrophil
Macrophage
T lymphocyte
Jak-STAT signaling pathway
JAK2/3
+p
STAT
PKA
cAMP
MAPK signaling pathway
Shc
GRB2
SOS
Ras
Raf
+p
MEK1
+p
ERK1/2
AC
PKCζ
+p
FOXO
Cytokine production
Cellular growth and differentiation
Cell survival
Migration
Apoptosis
DNA
Cytokine-cytokine receptor interaction
Gαi
Src
+p
PI3K
PIP3
Akt
NFκB
chemokine
chemokineR
+p
IKK
+p
IκB
+p
GSK3
Gβγ
+p
GRK
P-Rex1
Ubiquitin mediated proteolysis
β-arrestin
Receptor internalization
+p
DOCK2
ELMO1
Rac
Tiam1
Par3
PKCζ
PAK1
Regulation of actin cytoskeleton
Leukocyte transendothelial migration
Migration
Chemotaxis
Cellular shape changes
Metestasis
Degranulation
ROS production
Itk
Vav
Cdc42
WASP
+p
Pyk2
RhoA
ROCK
+p
+p
FAK
+p
Paxillin
p130Cas
Crk
PLCβ
IP3
Ca2+
CalDAG-GEF1
RasGRP2
Rap1
DAG
PKC
Degranulation
Chemotaxis
NO induction
NADPH oxidase
p47phox
ROS production
04062 8/2/13
(c) Kanehisa Laboratories

Figure S55

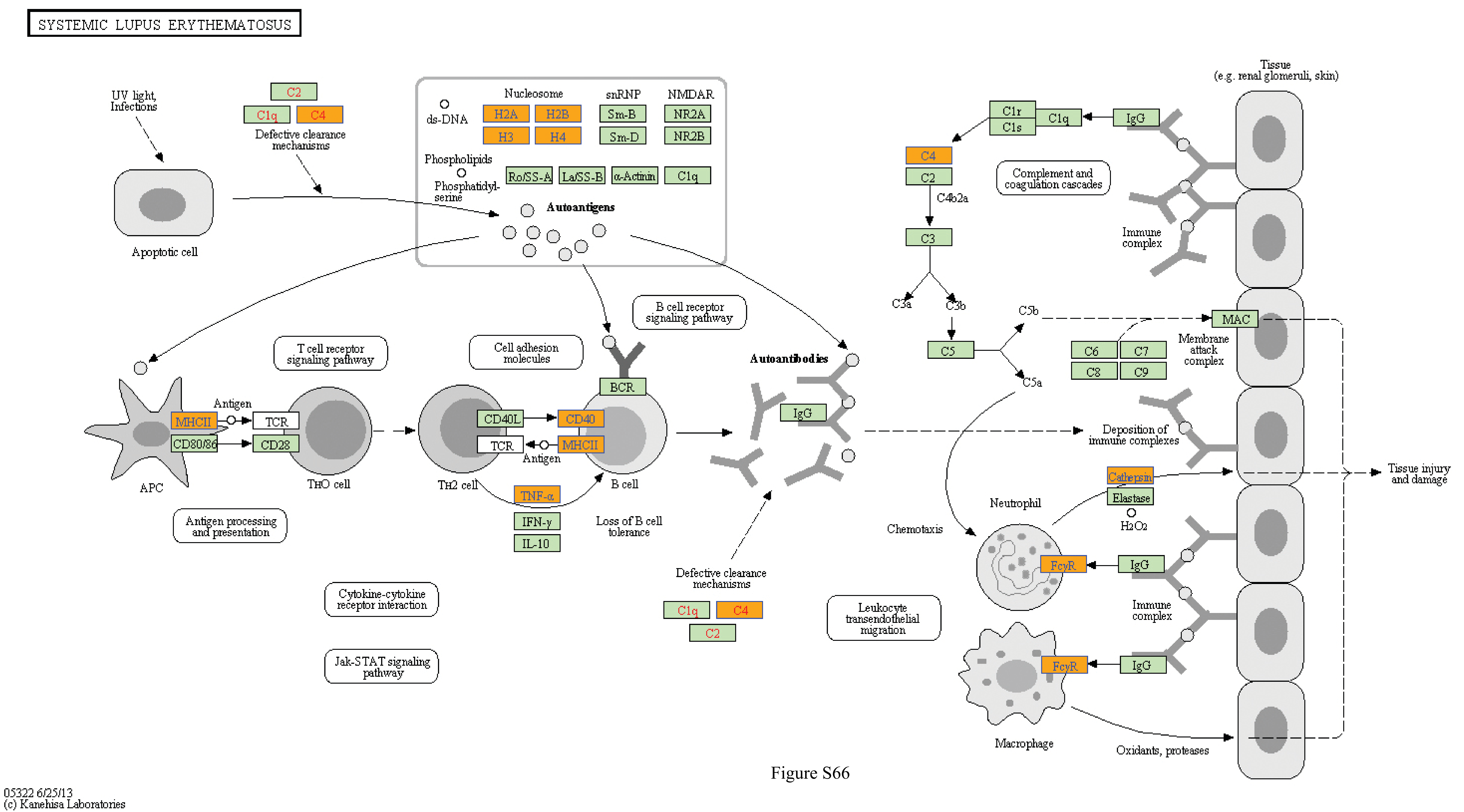
SYSTEMIC LUPUS ERYTHEMATOSUS
UV light, Infections
C2
C1q
C4
Defective clearance mechanisms
Apoptotic cell
ds-DNA
Nucleosome
H2A
H2B
H3
H4
snRNP
Sm-B
Sm-D
NMDAR
NR2A
NR2B
Phospholipids
Phosphatidyl-serine
Ro/SS-A
La/SS-B
α-Actinin
C1q
Autoantigens
T cell receptor signaling pathway
Cell adhesion molecules
B cell receptor signaling pathway
Autoantibodies
APC
MHCII
CD80/86
Antigen
TCR
CD28
THO cell
CD40L
CD40
TCR
MHCII
Antigen
TH2 cell
BCR
B cell
TNF-α
IFN-γ
IL-10
Loss of B cell tolerance
IgG
Antigen processing and presentation
Cytokine-cytokine receptor interaction
Jak-STAT signaling pathway
Defective clearance mechanisms
C1q
C4
C2
Leukocyte transendothelial migration
C1r
C1s
C1q
IgG
C4
C2
C4b2a
C3
C3a
C3b
C5
C5b
C5a
Complement and coagulation cascades
Immune complex
MAC
C6
C7
C8
C9
Membrane attack complex
Deposition of immune complexes
Cathepsin
Elastase
H2O2
Neutrophil
Chemotaxis
FcγR
IgG
Immune complex
FcγR
IgG
Macrophage
Oxidants, proteases
Tissue (e.g. renal glomeruli, skin)
Tissue injury and damage
05322 6/25/13
(c) Kanehisa Laboratories

Figure S66

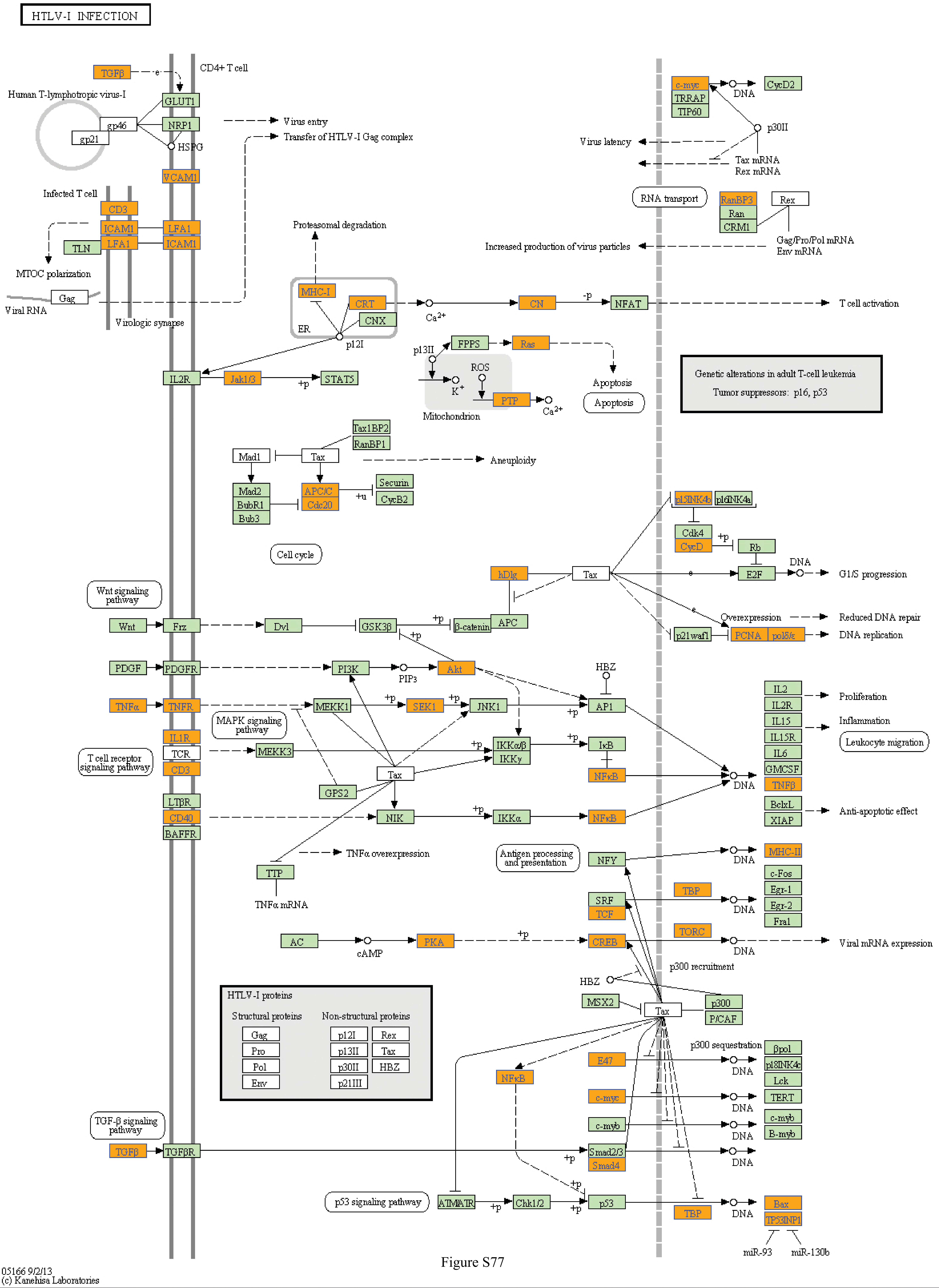
HTLV-I INFECTION
CD4+ T cell
Human T-lymphotropic virus-I
TGFβ
GLUT1
NRP1
HSPG
gp46
gp21
VCAM1
Virus entry
Transfer of HTLV-I Gag complex
Infected T cell
CD3
ICAM1
LFA1
TLN
MTOC polarization
Gag
Viral RNA
Virologic synapse
Proteasomal degradation
MHC-I
CRT
CNX
ER
p12I
Ca2+
CN
NFAT
T cell activation
IL2R
Jak1/3
STAT5
p13II
FPPS
Ras
ROS
K+
PTP
Mitochondrion
Apoptosis
Genetic alterations in adult T-cell leukemia
Tumor suppressors: p16, p53
c-myc
TRRAP
TIP60
DNA
CycD2
p30II
Virus latency
Tax mRNA
Rex mRNA
RNA transport
RanBP3
Ran
CRM1
Rex
Gag/Pro/Pol mRNA
Env mRNA
Increased production of virus particles
Tax1BP2
RanBP1
Mad1
Tax
Aneuploidy
Mad2
BubR1
Bub3
APC/C
Cdc20
Securin
CycB2
Cell cycle
p15INK4b
p16INK4a
Cdk4
CycD
Rb
E2F
G1/S progression
hDlg
APC
Overexpression
Reduced DNA repair
p21waf1
PCNA
polδ/ε
DNA replication
Wnt signaling pathway
Wnt
Frz
Dvl
GSK3β
β-catenin
PDGF
PDGFR
PI3K
PIP3
Akt
HBZ
AP1
TNFα
TNFR
MEKK1
SEK1
JNK1
MAPK signaling pathway
IL1R
TCR
T cell receptor signaling pathway
MEKK3
IKKα/β
IKKγ
IκB
NFκB
GPS2
LTβR
CD40
BAFFR
NIK
IKKα
IL2
IL15
IL15R
IL6
GMCSF
TNFβ
Proliferation
Inflammation
Leukocyte migration
BclxL
XIAP
Anti-apoptotic effect
TTP
TNFα mRNA
TNFα overexpression
Antigen processing and presentation
NFY
MHC-II
SRF
TCF
TBP
c-Fos
Egr-1
Egr-2
Fra1
AC
cAMP
PKA
CREB
TORC
Viral mRNA expression
p300 recruitment
MSX2
p300
P/CAF
HTLV-I proteins
Structural proteins
Non-structural proteins
Pro
Pol
Env
p21III
p300 sequestration
E47
βpol
p18INK4c
Lck
TERT
c-myb
B-myb
TGF-β signaling pathway
TGFβR
Smad2/3
Smad4
p53 signaling pathway
ATM/ATR
Chk1/2
p53
Bax
TP53INP1
miR-93
miR-130b
+p
-p
+u
05166 9/2/13
(c) Kanehisa Laboratories

Figure S77

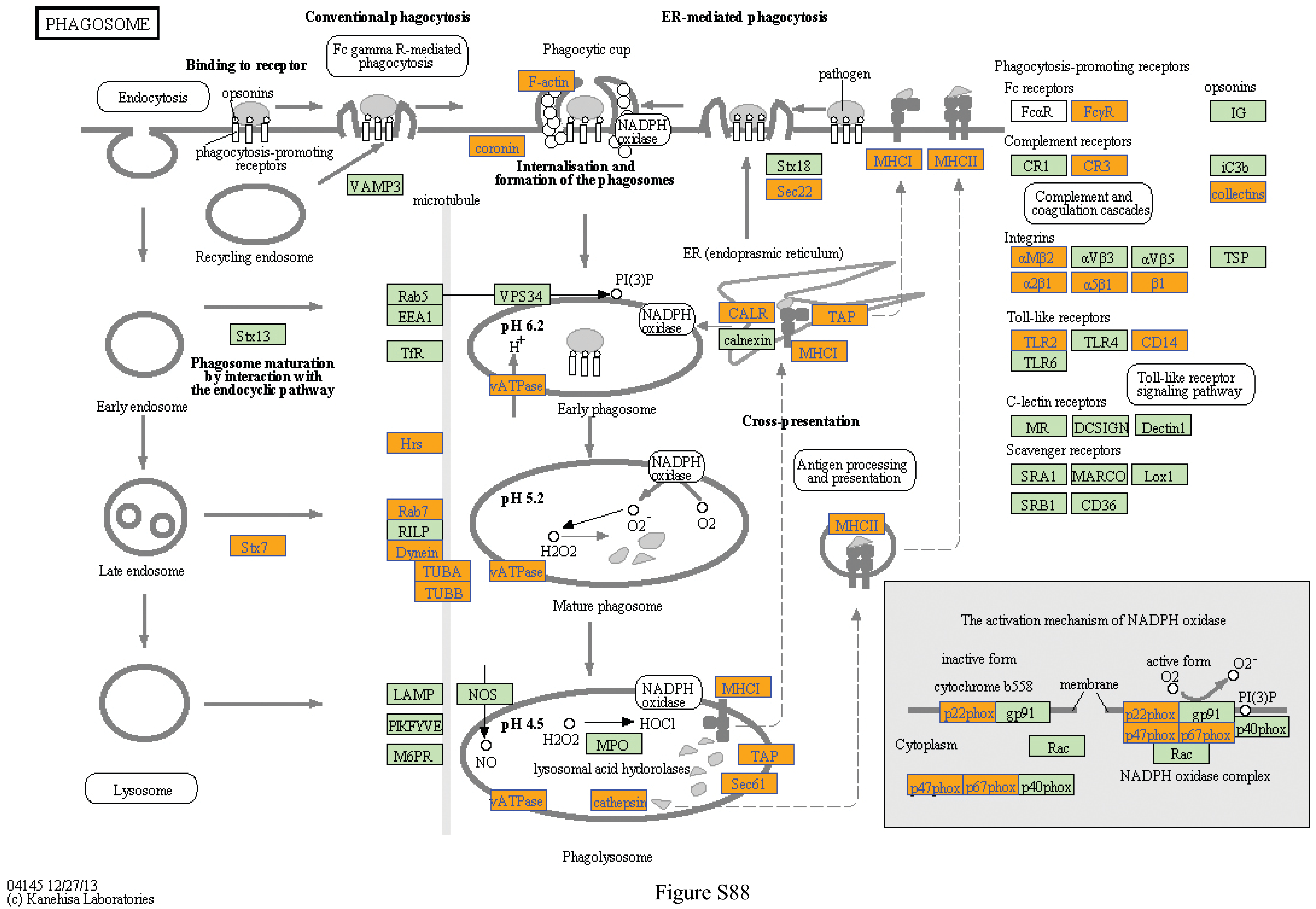
PHAGOSOME
Conventional phagocytosis
ER-mediated phagocytosis
Binding to receptor
Fc gamma R-mediated phagocytosis
Phagocytic cup
pathogen
Endocytosis
opsonins
phagocytosis-promoting receptors
F-actin
NADPH oxidase
coronin
VAMP3
microtubule
Internalisation and formation of the phagosomes
Stx18
Sec22
MHCI
MHCII
Recycling endosome
ER (endoprasmic reticulum)
Rab5
EEA1
TfR
VPS34
PI(3)P
pH 6.2
H+
vATPase
Early phagosome
CALR
calnexin
TAP
Stx13
Phagosome maturation by interaction with the endocyclic pathway
Early endosome
Hrs
Cross-presentation
Antigen processing and presentation
pH 5.2
O2-
O2
H2O2
Rab7
RILP
Dynein
TUBA
TUBB
Stx7
Late endosome
Mature phagosome
LAMP
PIKFYVE
M6PR
NOS
pH 4.5
HOCl
MPO
NO
lysosomal acid hydrolases
Sec61
cathepsin
Lysosome
Phagolysosome
Phagocytosis-promoting receptors
Fc receptors
FcαR
FcγR
opsonins
IG
Complement receptors
CR1
CR3
iC3b
collectins
Complement and coagulation cascades
Integrins
αMβ2
αVβ3
αVβ5
TSP
α2β1
α5β1
β1
Toll-like receptors
TLR2
TLR4
CD14
TLR6
Toll-like receptor signaling pathway
C-lectin receptors
MR
DCSIGN
Dectin1
Scavenger receptors
SRA1
MARCO
Lox1
SRB1
CD36
The activation mechanism of NADPH oxidase
inactive form
active form
cytochrome b558
membrane
p22phox
gp91
p47phox
p67phox
p40phox
Cytoplasm
Rac
NADPH oxidase complex
04145 12/27/13
(c) Kanehisa Laboratories

Figure S88

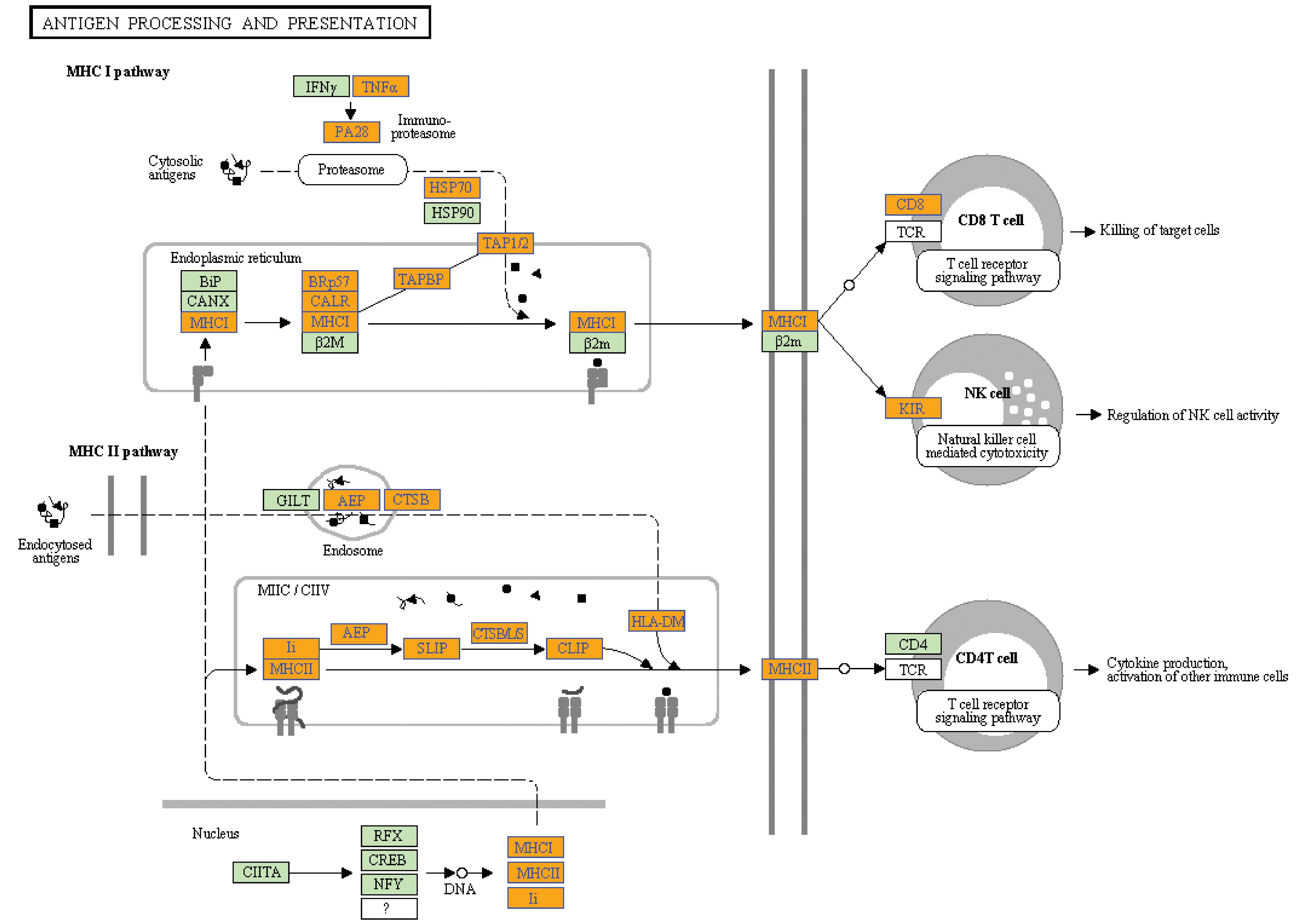

Figure S9

(c) Kanehisa Laboratories

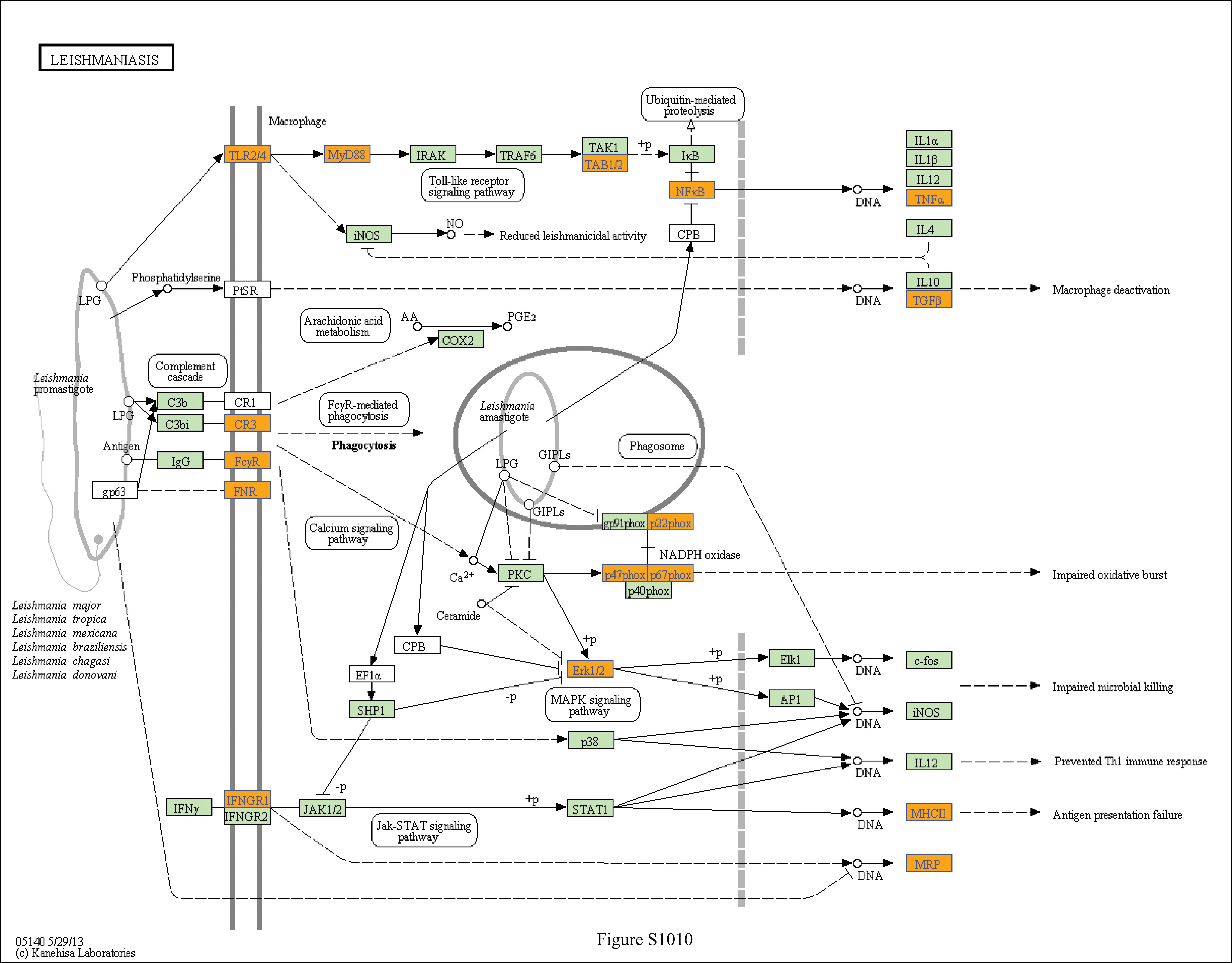

Figure S1010

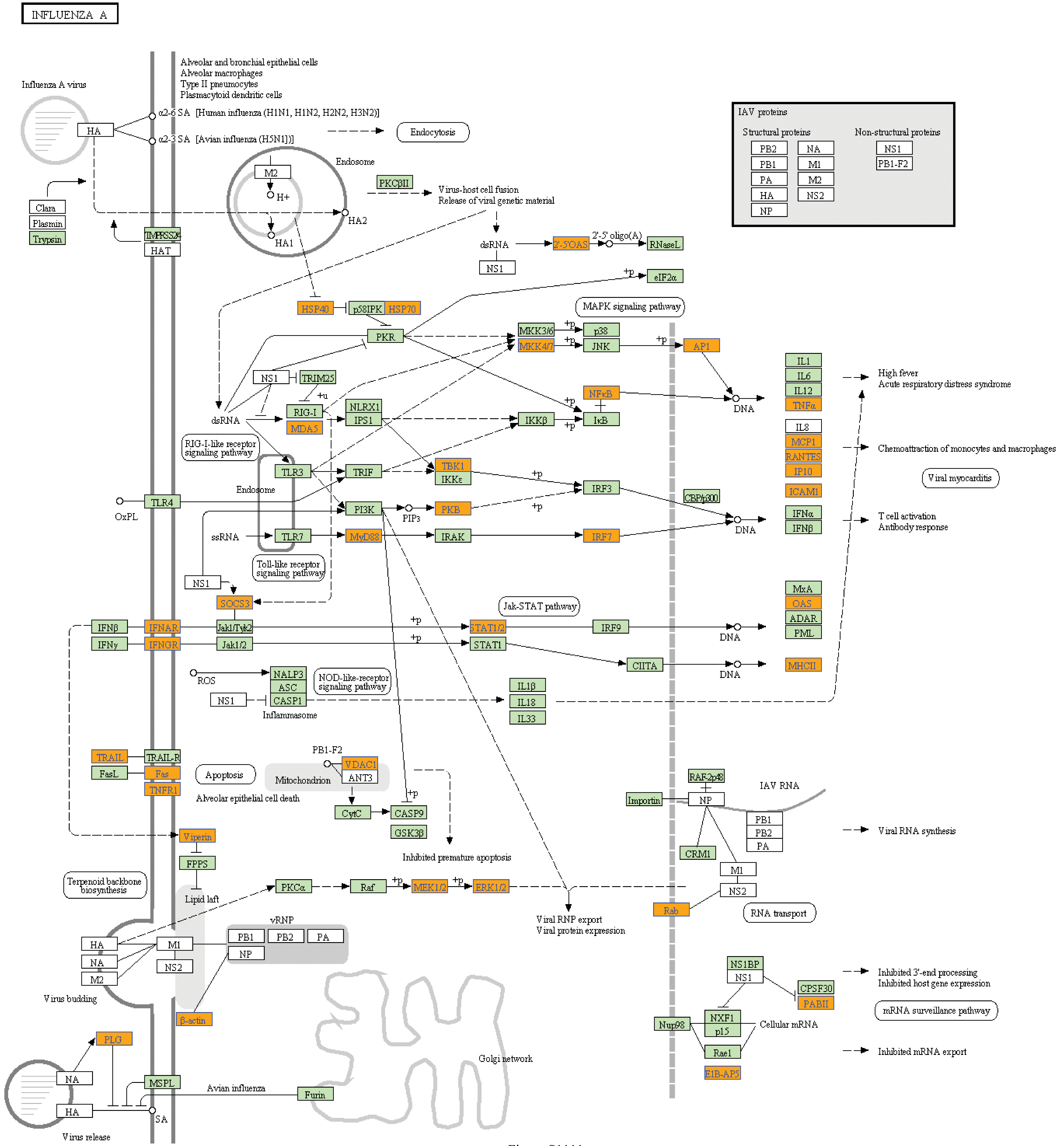

Figure S1111

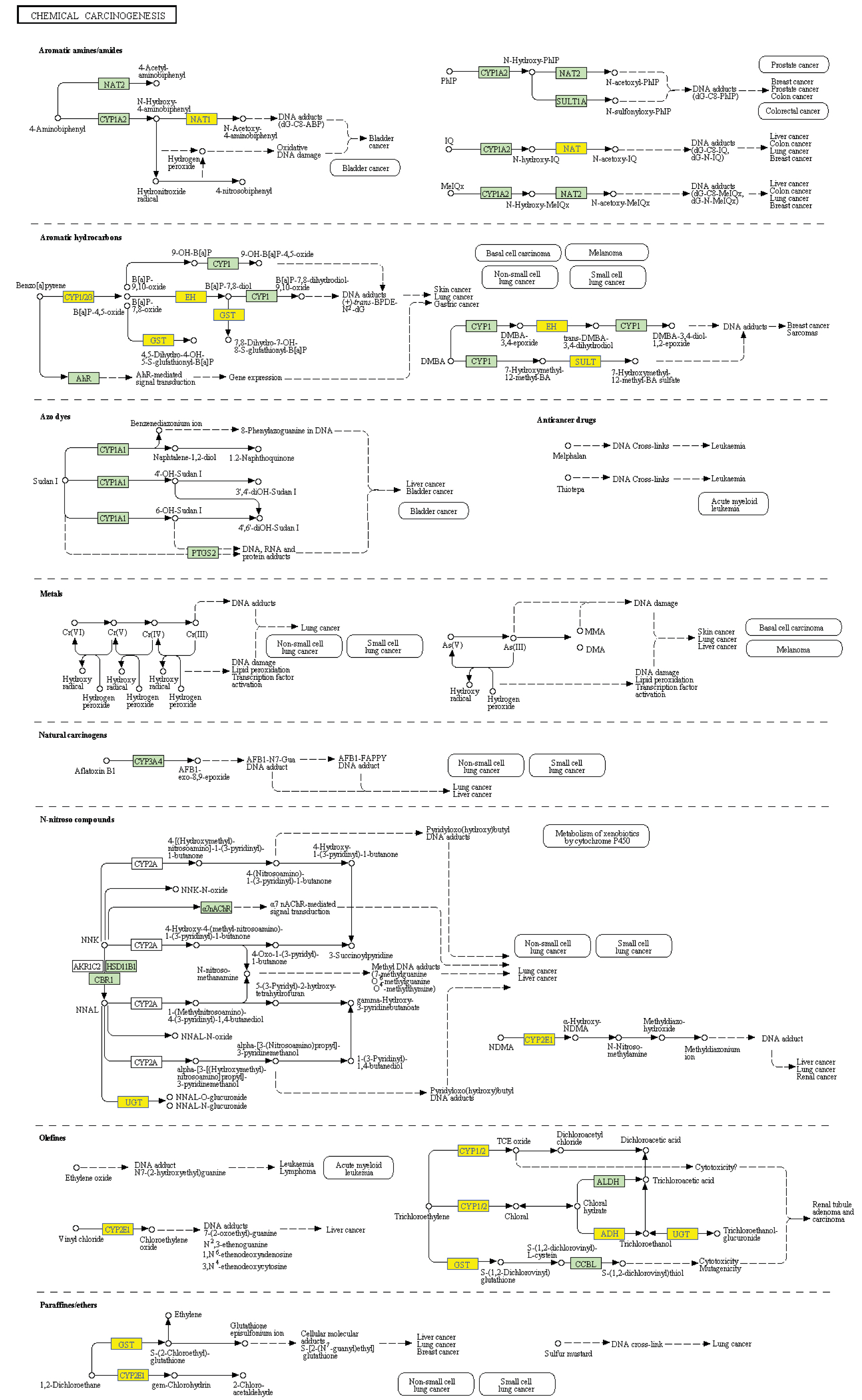

05204 11/11/13
(c) Kanehisa Laboratories

Figure S12

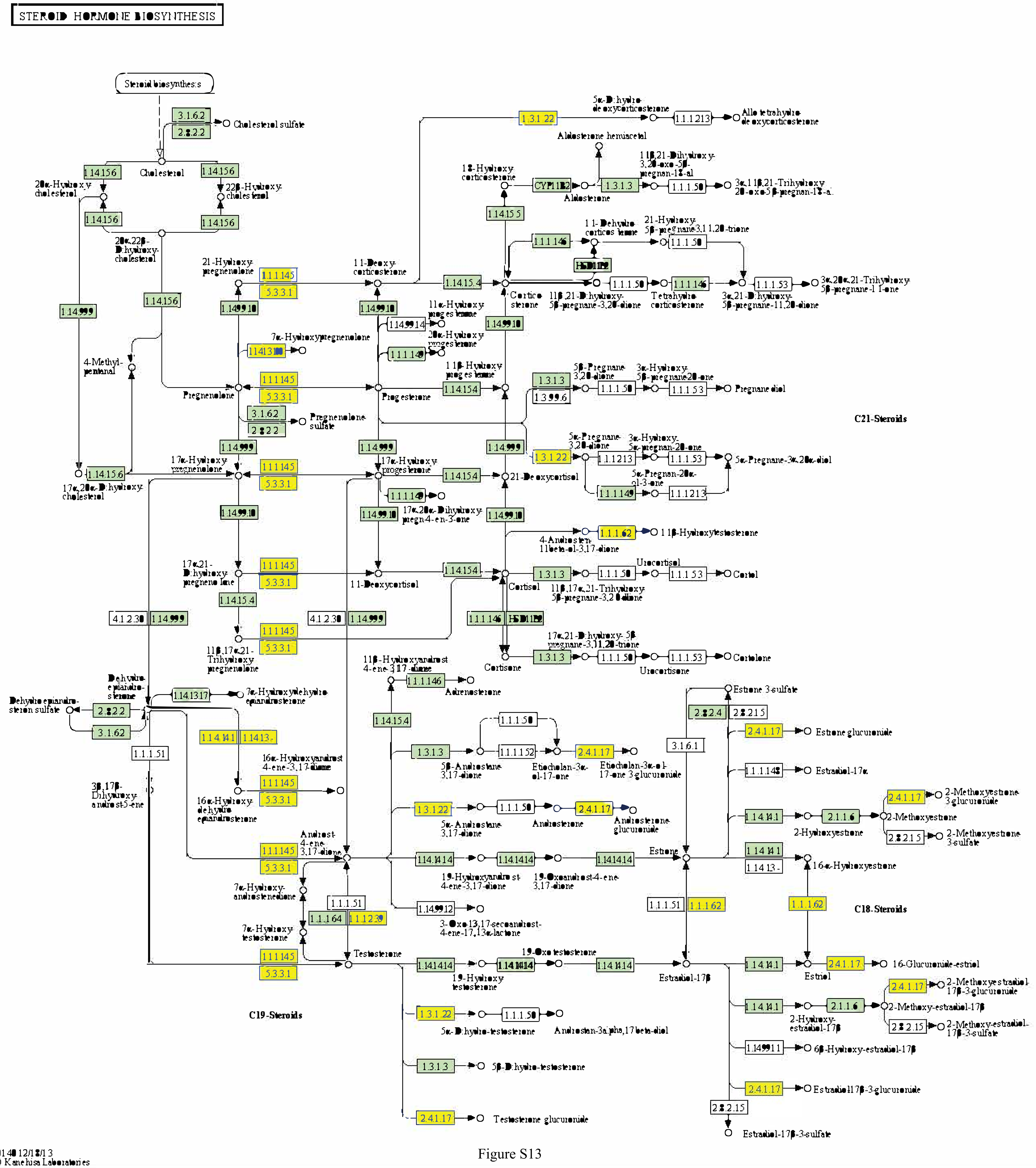
STEROID HORMONE BIOSYNTHESIS
Steroid biosynthesis
3.1.6.2
2.8.2.2
Cholesterol sulfate
Cholesterol
1.14.15.6
20α-Hydroxy-cholesterol
22β-Hydroxy-cholesterol
20α,22β-Dihydroxy-cholesterol
4-Methyl-pentanal
1.14.99.-
17α,20α-Dihydroxy-cholesterol
21-Hydroxy-pregnenolone
1.1.1.145
5.3.3.1
1.14.99.10
11-Deoxy-corticosterone
1.3.1.22
5α-Dihydro-deoxycorticosterone
1.1.1.213
Allo-tetrahydro-deoxycorticosterone
Aldosterone hemiacetal
18-Hydroxy-corticosterone
CYP11B2
1.3.1.3
Aldosterone
1.1.1.50
11β,21-Dihydroxy-3,20-oxo-5β-pregnan-18-al
3α,11β,21-Trihydroxy-20-oxo-5β-pregnan-18-al
1.14.15.5
11-Dehydro-corticosterone
21-Hydroxy-5β-pregnane-3,11,20-trione
1.1.1.146
HSD11B2
1.14.15.4
Corticosterone
11β,21-Dihydroxy-5β-pregnane-3,20-dione
Tetrahydro-corticosterone
3α,21-Dihydroxy-5β-pregnane-11,20-dione
1.1.1.53
3α,20α,21-Trihydroxy-5β-pregnane-11-one
11α-Hydroxy-progesterone
1.14.99.14
20α-Hydroxy-progesterone
1.1.1.149
7α-Hydroxypregnenolone
1.14.13.100
11β-Hydroxy-progesterone
1.14.15.4
5β-Pregnane-3,20-dione
3α-Hydroxy-5β-pregnan-20-one
Pregnanediol
1.3.99.6
Pregnenolone
Progesterone
3.1.6.2
2.8.2.2
Pregnenolone sulfate
C21-Steroids
5α-Pregnane-3,20-dione
3α-Hydroxy-5α-pregnan-20-one
5α-Pregnane-3α,20α-diol
5α-Pregnan-20α-ol-3-one
17α-Hydroxy-pregnenolone
17α-Hydroxy-progesterone
21-Deoxycortisol
17α,20α-Dihydroxy-pregn-4-en-3-one
4-Androsten-11beta-ol-3,17-dione
1.1.1.62
11β-Hydroxytestosterone
17α,21-Dihydroxy-pregnenolone
11-Deoxycortisol
Cortisol
Urocortisol
Cortol
11β,17α,21-Trihydroxy-5β-pregnane-3,20-dione
4.1.2.30
11β,17α,21-Trihydroxy-pregnenolone
Cortisone
17α,21-Dihydroxy-5β-pregnane-3,11,20-trione
Urocortisone
Cortolone
11β-Hydroxyandrost-4-ene-3,17-dione
Adrenosterone
Dehydroepiandrosterone
Dehydroepiandrosterone sulfate
1.14.13.17
7α-Hydroxydehydroepiandrosterone
1.14.14.1
1.14.13.-
1.1.1.51
16α-Hydroxyandrost-4-ene-3,17-dione
16α-Hydroxy-dehydroepiandrosterone
3β,17β-Dihydroxy-androst-5-ene
1.1.1.50
1.1.1.52
5β-Androstane-3,17-dione
Etiocholan-3α-ol-17-one
2.4.1.17
Etiocholan-3α-ol-17-one 3-glucuronide
5α-Androstane-3,17-dione
Androsterone
Androsterone glucuronide
Androst-4-ene-3,17-dione
1.14.14.14
19-Hydroxyandrost-4-ene-3,17-dione
19-Oxoandrost-4-ene-3,17-dione
Estrone
7α-Hydroxy-androstenedione
7α-Hydroxy-testosterone
1.1.1.64
1.1.1.239
1.14.99.12
3-Oxo-13,17-secoandrost-4-ene-17,13α-lactone
Testosterone
19-Oxotestosterone
19-Hydroxy-testosterone
Estradiol-17β
C19-Steroids
5α-Dihydro-testosterone
Androstan-3alpha,17beta-diol
5β-Dihydro-testosterone
Testosterone glucuronide
Estrone 3-sulfate
2.8.2.4
2.8.2.15
3.1.6.1
Estrone glucuronide
1.1.1.148
Estradiol-17α
2.1.1.6
2-Hydroxyestrone
2-Methoxyestrone
2-Methoxyestrone 3-glucuronide
2-Methoxyestrone 3-sulfate
16α-Hydroxyestrone
C18-Steroids
Estriol
16-Glucuronide-estriol
2-Hydroxy-estradiol-17β
2-Methoxy-estradiol-17β
2-Methoxyestradiol-17β-3-glucuronide
2-Methoxy-estradiol-17β-3-sulfate
1.14.99.11
6β-Hydroxy-estradiol-17β
Estradiol-17β-3-glucuronide
Estradiol-17β-3-sulfate
00140 12/18/13
(c) Kanehisa Laboratories

Figure S13

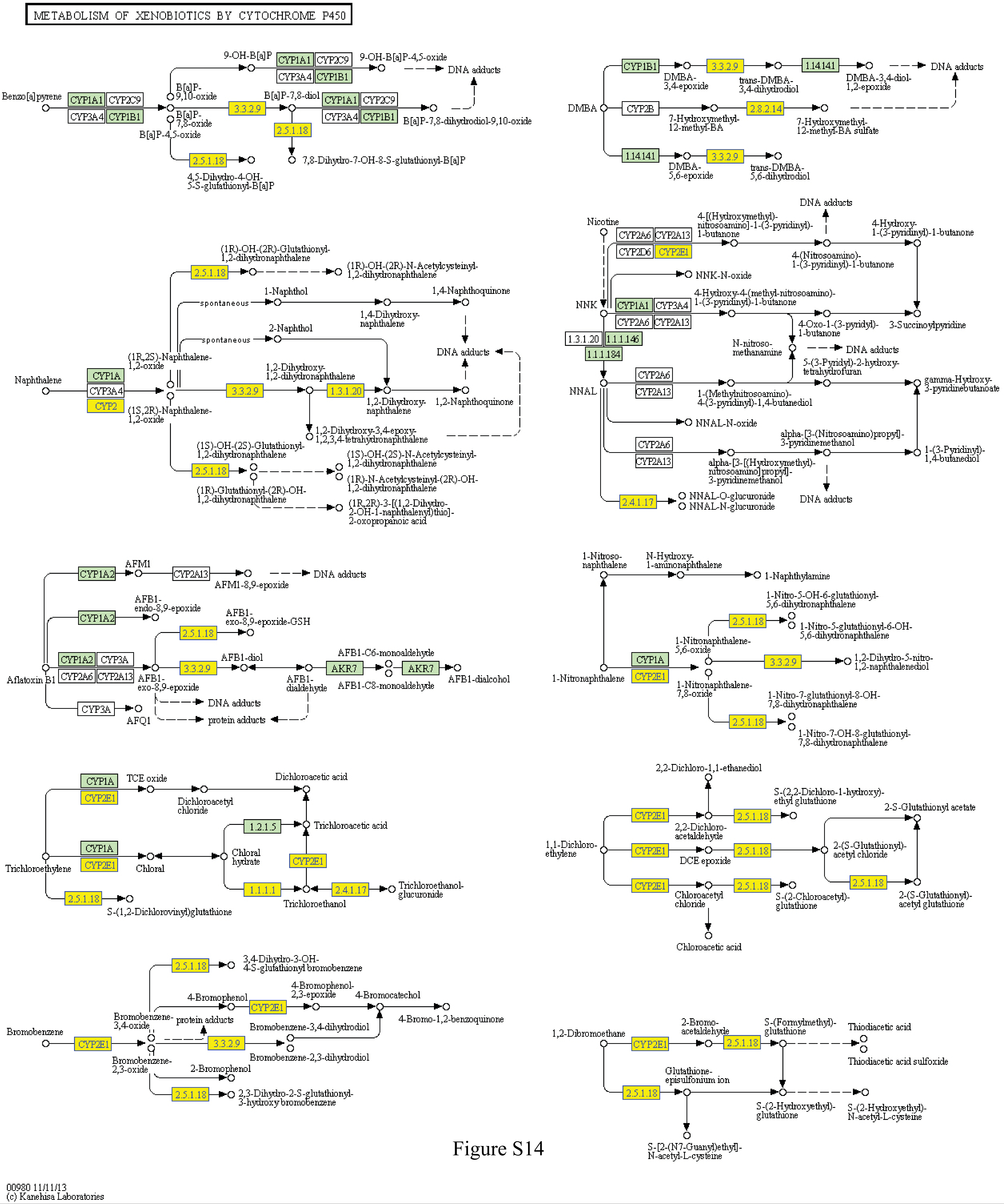

METABOLISM OF XENOBIOTICS BY CYTOCHROME P450
Benzo[a]pyrene
9-OH-B[a]P
9-OH-B[a]P-4,5-oxide
DNA adducts
B[a]P-9,10-oxide
B[a]P-7,8-diol
B[a]P-7,8-oxide
B[a]P-4,5-oxide
B[a]P-7,8-dihydrodiol-9,10-oxide
7,8-Dihydro-7-OH-8-S-glutathionyl-B[a]P
4,5-Dihydro-4-OH-5-S-glutathionyl-B[a]P
DMBA
DMBA-3,4-epoxide
trans-DMBA-3,4-dihydrodiol
DMBA-3,4-diol-1,2-epoxide
7-Hydroxymethyl-12-methyl-BA
7-Hydroxymethyl-12-methyl-BA sulfate
DMBA-5,6-epoxide
trans-DMBA-5,6-dihydrodiol
Nicotine
NNK
NNAL
NNK-N-oxide
NNAL-N-oxide
NNAL-O-glucuronide
NNAL-N-glucuronide
Naphthalene
1-Naphthol
2-Naphthol
1,4-Naphthoquinone
1,2-Naphthoquinone
Aflatoxin B1
AFM1
AFM1-8,9-epoxide
AFB1-exo-8,9-epoxide
AFB1-diol
AFQ1
protein adducts
1-Nitronaphthalene
1-Naphthylamine
Trichloroethylene
Chloral hydrate
Trichloroacetic acid
Dichloroacetic acid
Trichloroethanol
Bromobenzene
4-Bromophenol
2-Bromophenol
4-Bromocatechol
1,1-Dichloroethylene
Chloroacetic acid
1,2-Dibromoethane
Thiodiacetic acid
00980 11/11/13
(c) Kanehisa Laboratories

Figure S14

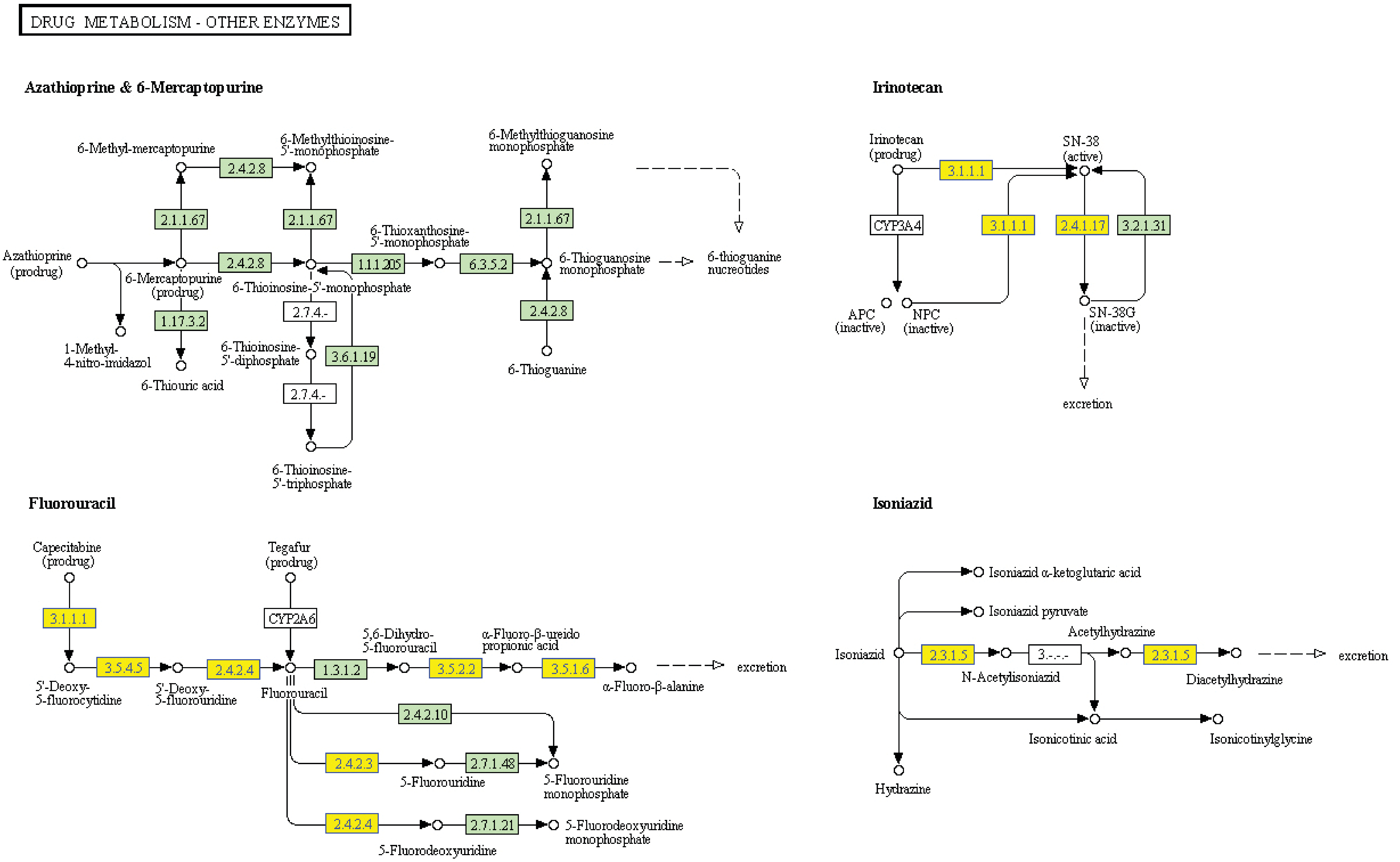

Figure S15

00983 10/28/13
(c) Kanehisa Laboratories

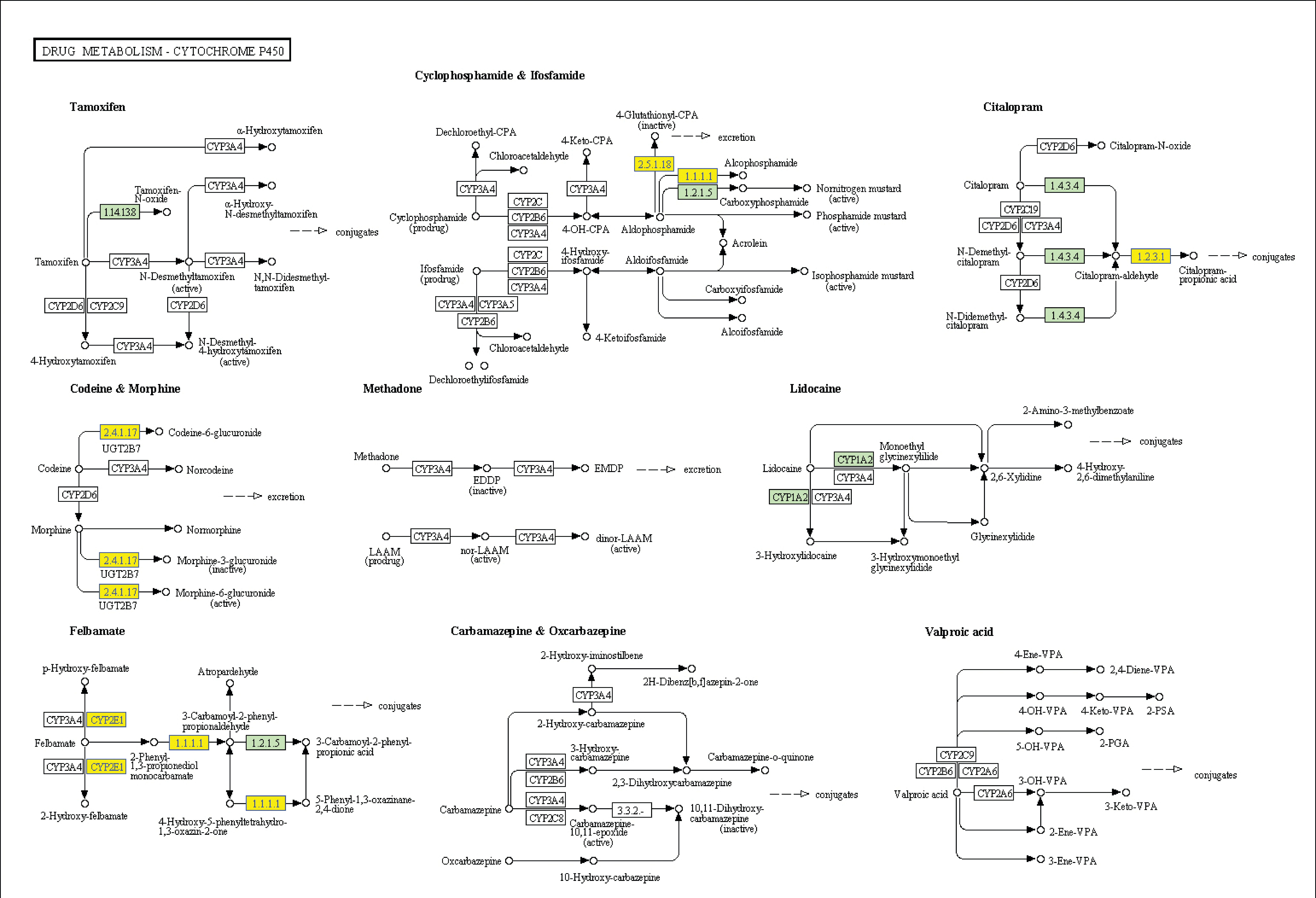

DRUG METABOLISM - CYTOCHROME P450
Tamoxifen
α-Hydroxytamoxifen
CYP3A4
Tamoxifen-N-oxide
1.14.13.8
α-Hydroxy-N-desmethyltamoxifen
conjugates
Tamoxifen
N-Desmethyltamoxifen (active)
N,N-Didesmethyl-tamoxifen
CYP2D6
CYP2C9
4-Hydroxytamoxifen
N-Desmethyl-4-hydroxytamoxifen (active)
Cyclophosphamide & Ifosfamide
Dechloroethyl-CPA
Chloroacetaldehyde
4-Keto-CPA
4-Glutathionyl-CPA (inactive)
excretion
2.5.1.18
1.1.1.1
1.2.1.5
Alcophosphamide
Carboxyphosphamide
Nornitrogen mustard (active)
Cyclophosphamide (prodrug)
CYP2C
CYP2B6
4-OH-CPA
Aldophosphamide
Phosphamide mustard (active)
Acrolein
Ifosfamide (prodrug)
4-Hydroxy-ifosfamide
Aldoifosfamide
Isophosphamide mustard (active)
Carboxyifosfamide
CYP3A5
4-Ketoifosfamide
Alcoifosfamide
Dechloroethylifosfamide
Citalopram
Citalopram-N-oxide
1.4.3.4
CYP2C19
N-Demethyl-citalopram
Citalopram-aldehyde
1.2.3.1
Citalopram-propionic acid
N-Didemethyl-citalopram
Codeine & Morphine
2.4.1.17
UGT2B7
Codeine-6-glucuronide
Codeine
Norcodeine
Morphine
Normorphine
Morphine-3-glucuronide (inactive)
Morphine-6-glucuronide (active)
Methadone
EDDP (inactive)
EMDP
LAAM (prodrug)
nor-LAAM (active)
dinor-LAAM (active)
Lidocaine
2-Amino-3-methylbenzoate
Monoethyl glycinexylilide
CYP1A2
2,6-Xylidine
4-Hydroxy-2,6-dimethylaniline
Glycinexylidide
3-Hydroxylidocaine
3-Hydroxymonoethyl glycinexylidide
Felbamate
p-Hydroxy-felbamate
Atropardehyde
CYP2E1
3-Carbamoyl-2-phenyl-propionaldehyde
2-Phenyl-1,3-propionediol monocarbamate
3-Carbamoyl-2-phenyl-propionic acid
5-Phenyl-1,3-oxazinane-2,4-dione
2-Hydroxy-felbamate
4-Hydroxy-5-phenyltetrahydro-1,3-oxazin-2-one
Carbamazepine & Oxcarbazepine
2-Hydroxy-iminostilbene
2H-Dibenz[b,f]azepin-2-one
2-Hydroxy-carbamazepine
3-Hydroxy-carbamazepine
Carbamazepine-o-quinone
2,3-Dihydroxycarbamazepine
CYP2C8
3.3.2.-
Carbamazepine
Carbamazepine-10,11-epoxide (active)
10,11-Dihydroxy-carbamazepine (inactive)
Oxcarbazepine
10-Hydroxy-carbazepine
Valproic acid
4-Ene-VPA
2,4-Diene-VPA
4-OH-VPA
4-Keto-VPA
2-PSA
5-OH-VPA
2-PGA
CYP2C9
CYP2A6
3-OH-VPA
3-Keto-VPA
Valproic acid
2-Ene-VPA
3-Ene-VPA
00982 11/11/13
(c) Kanehisa Laboratories

Figure S16

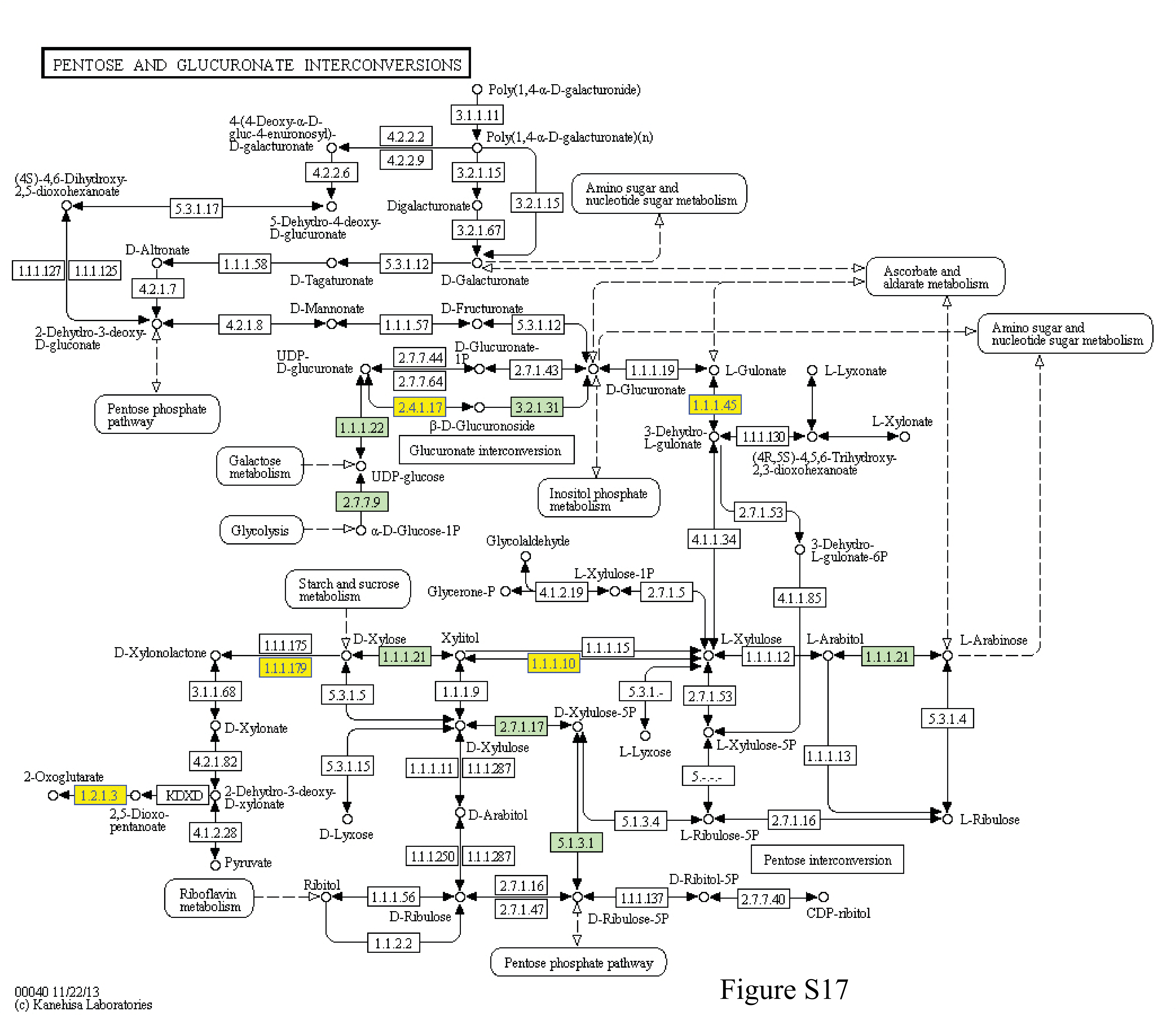
PENTOSE AND GLUCURONATE INTERCONVERSIONS
Poly(1,4-α-D-galacturonide)
3.1.1.11
Poly(1,4-α-D-galacturonate)(n)
4-(4-Deoxy-α-D-gluc-4-enuronosyl)-D-galacturonate
4.2.2.2
4.2.2.9
4.2.2.6
3.2.1.15
3.2.1.15
(4S)-4,6-Dihydroxy-2,5-dioxohexanoate
5.3.1.17
5-Dehydro-4-deoxy-D-glucuronate
Digalacturonate
3.2.1.67
Amino sugar and nucleotide sugar metabolism
D-Altronate
1.1.1.58
5.3.1.12
1.1.1.127
1.1.1.125
4.2.1.7
D-Tagaturonate
D-Galacturonate
Ascorbate and aldarate metabolism
2-Dehydro-3-deoxy-D-gluconate
4.2.1.8
D-Mannonate
1.1.1.57
D-Fructuronate
5.3.1.12
Amino sugar and nucleotide sugar metabolism
UDP-D-glucuronate
2.7.7.44
2.7.7.64
D-Glucuronate-1P
2.7.1.43
1.1.1.19
L-Gulonate
L-Lyxonate
D-Glucuronate
2.4.1.17
3.2.1.31
1.1.1.45
Pentose phosphate pathway
1.1.1.22
β-D-Glucuronoside
3-Dehydro-L-gulonate
1.1.1.130
L-Xylonate
Glucuronate interconversion
(4R,5S)-4,5,6-Trihydroxy-2,3-dioxohexanoate
Galactose metabolism
UDP-glucose
Inositol phosphate metabolism
2.7.7.9
2.7.1.53
Glycolysis
α-D-Glucose-1P
4.1.1.34
3-Dehydro-L-gulonate-6P
Glycolaldehyde
L-Xylulose-1P
Starch and sucrose metabolism
Glycerone-P
4.1.2.19
2.7.1.5
4.1.1.85
1.1.1.175
D-Xylose
Xylitol
1.1.1.15
L-Xylulose
L-Arabitol
L-Arabinose
D-Xylonolactone
1.1.1.179
1.1.1.21
1.1.1.10
1.1.1.12
1.1.1.21
3.1.1.68
5.3.1.5
1.1.1.9
5.3.1.-
2.7.1.53
5.3.1.4
D-Xylonate
2.7.1.17
D-Xylulose-5P
L-Xylulose-5P
D-Xylulose
L-Lyxose
4.2.1.82
5.3.1.15
1.1.1.11
1.1.1.287
5.-.-.-
1.1.1.13
2-Oxoglutarate
1.2.1.3
KDXD
2-Dehydro-3-deoxy-D-xylonate
2,5-Dioxo-pentanoate
D-Arabitol
5.1.3.4
2.7.1.16
L-Ribulose
4.1.2.28
D-Lyxose
L-Ribulose-5P
5.1.3.1
Pyruvate
Pentose interconversion
1.1.1.250
1.1.1.287
D-Ribitol-5P
Riboflavin metabolism
Ribitol
1.1.1.56
2.7.1.16
2.7.1.47
1.1.1.137
2.7.7.40
CDP-ribitol
D-Ribulose
D-Ribulose-5P
1.1.2.2
Pentose phosphate pathway
00040 11/22/13
(c) Kanehisa Laboratories

Figure S17

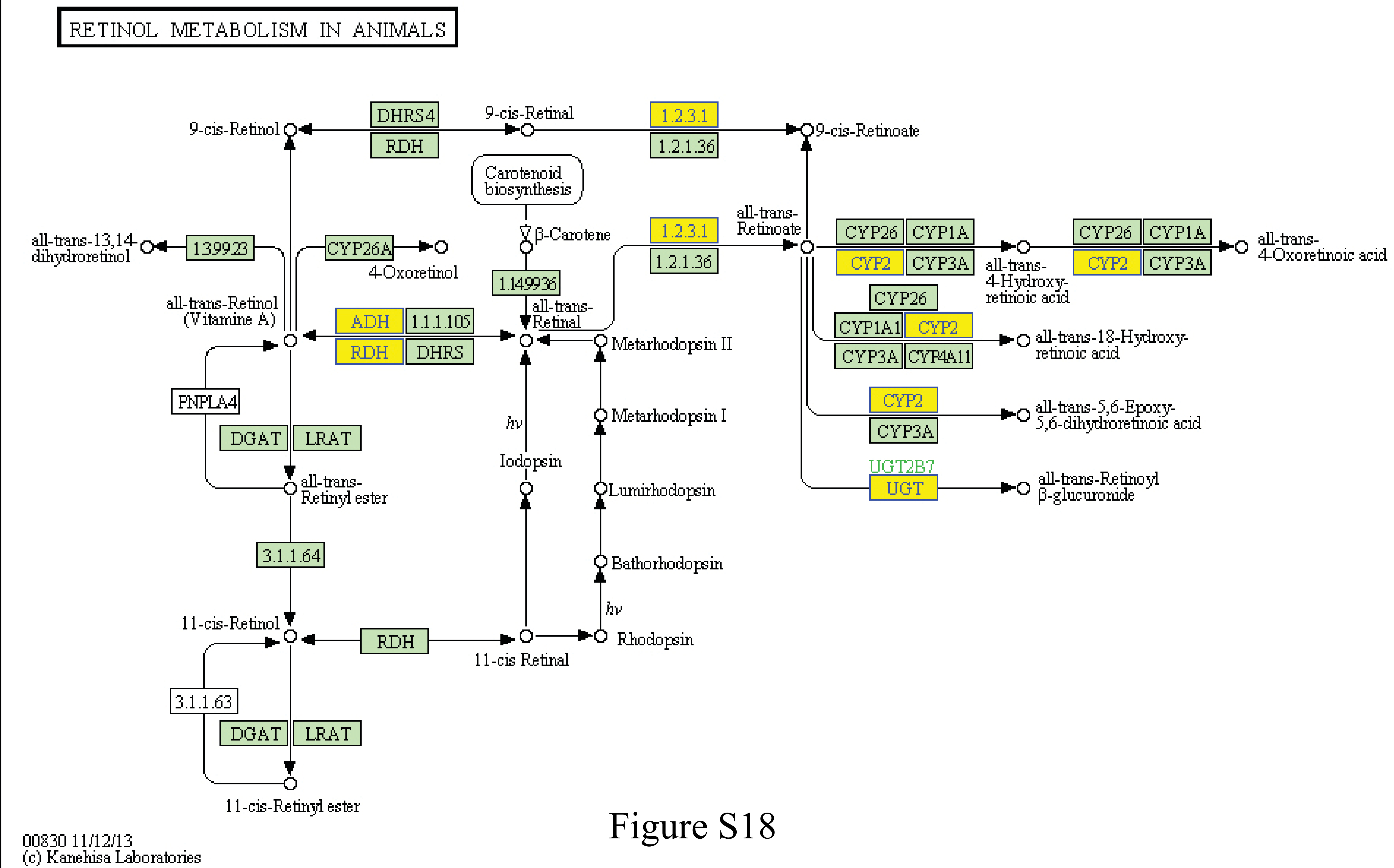
RETINOL METABOLISM IN ANIMALS
9-cis-Retinol
DHRS4
RDH
9-cis-Retinal
1.2.3.1
1.2.1.36
9-cis-Retinoate
Carotenoid biosynthesis
β-Carotene
all-trans-13,14-dihydroretinol
139923
CYP26A
4-Oxoretinol
1.14.99.36
all-trans-Retinal
1.2.3.1
1.2.1.36
all-trans-Retinoate
CYP26
CYP1A
CYP2
CYP3A
all-trans-4-Hydroxy-retinoic acid
CYP26
CYP1A
CYP2
CYP3A
all-trans-4-Oxoretinoic acid
all-trans-Retinol (Vitamine A)
ADH
1.1.1.105
RDH
DHRS
Metarhodopsin II
CYP26
CYP1A1
CYP2
CYP3A
CYP4A11
all-trans-18-Hydroxy-retinoic acid
PNPLA4
DGAT
LRAT
hν
Metarhodopsin I
CYP2
CYP3A
all-trans-5,6-Epoxy-5,6-dihydroretinoic acid
Iodopsin
UGT2B7
UGT
all-trans-Retinoyl β-glucuronide
all-trans-Retinyl ester
Lumirhodopsin
3.1.1.64
Bathorhodopsin
hν
11-cis-Retinol
RDH
11-cis Retinal
Rhodopsin
3.1.1.63
DGAT
LRAT
11-cis-Retinyl ester
00830 11/12/13
(c) Kanehisa Laboratories

Figure S18

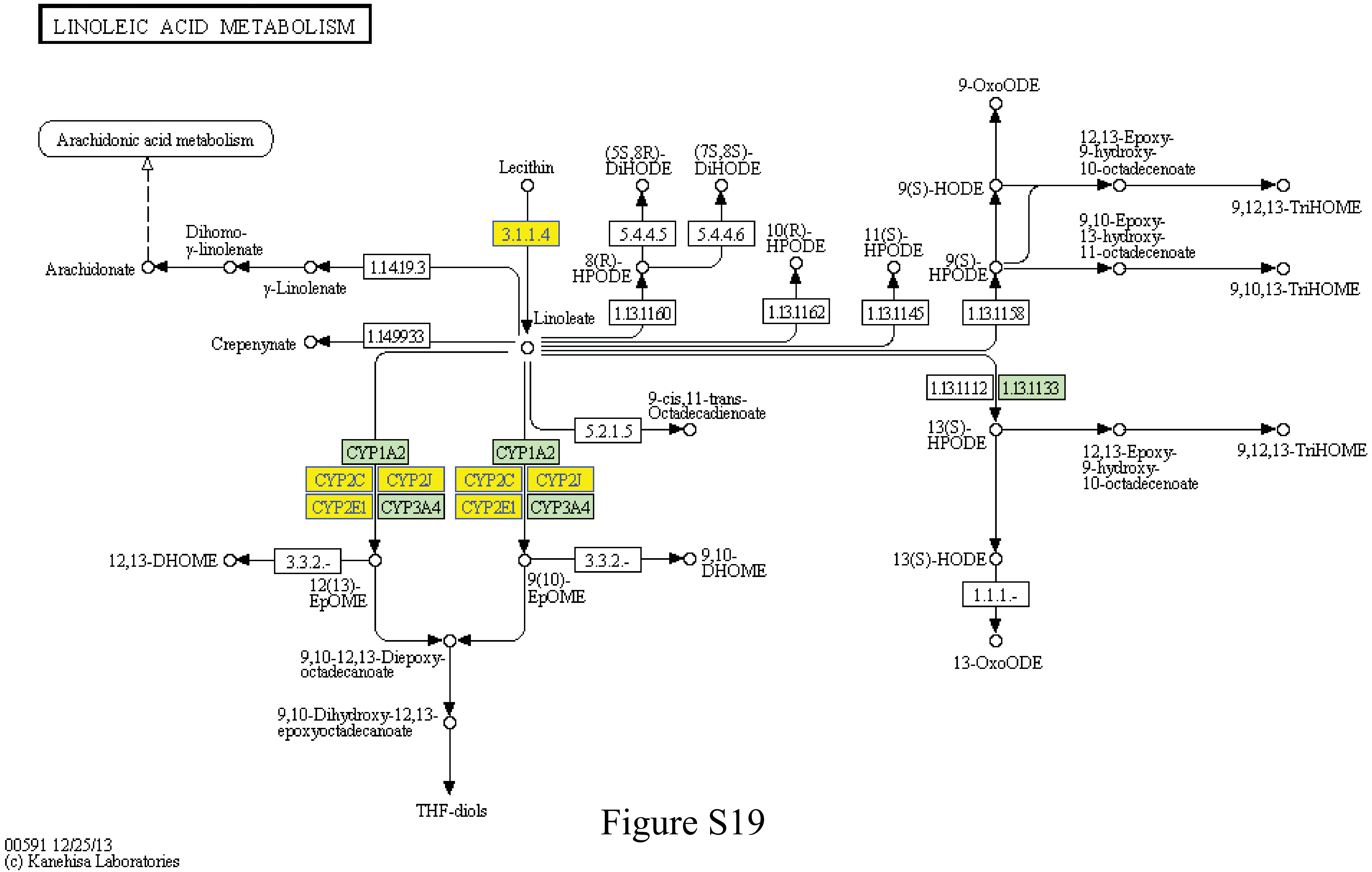
LINOLEIC ACID METABOLISM
Arachidonic acid metabolism
Arachidonate
Dihomo-γ-linolenate
γ-Linolenate
1.14.19.3
Lecithin
3.1.1.4
Linoleate
Crepenynate
1.14.99.33
(5S,8R)-DiHODE
(7S,8S)-DiHODE
5.4.4.5
5.4.4.6
8(R)-HPODE
1.13.11.60
10(R)-HPODE
1.13.11.62
11(S)-HPODE
1.13.11.45
9-OxoODE
9(S)-HODE
9(S)-HPODE
1.13.11.58
12,13-Epoxy-9-hydroxy-10-octadecenoate
9,12,13-TriHOME
9,10-Epoxy-13-hydroxy-11-octadecenoate
9,10,13-TriHOME
1.13.11.12
1.13.11.33
13(S)-HPODE
12,13-Epoxy-9-hydroxy-10-octadecenoate
9,12,13-TriHOME
13(S)-HODE
1.1.1.-
13-OxoODE
9-cis,11-trans-Octadecadienoate
5.2.1.5
CYP1A2
CYP2C
CYP2J
CYP2E1
CYP3A4
CYP1A2
CYP2C
CYP2J
CYP2E1
CYP3A4
12,13-DHOME
3.3.2.-
12(13)-EpOME
9(10)-EpOME
3.3.2.-
9,10-DHOME
9,10-12,13-Diepoxy-octadecanoate
9,10-Dihydroxy-12,13-epoxyoctadecanoate
THF-diols
00591 12/25/13
(c) Kanehisa Laboratories

Figure S19

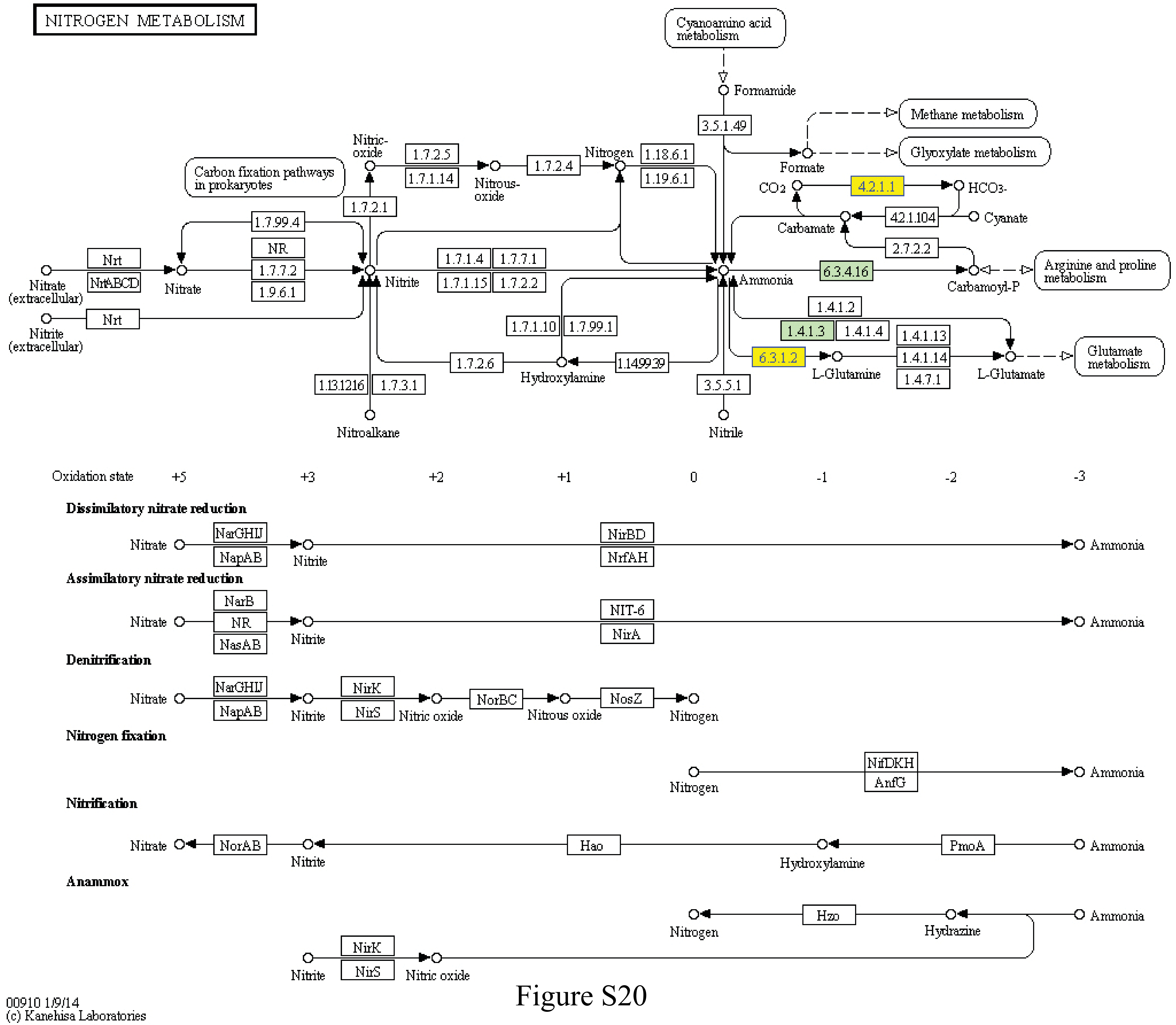
NITROGEN METABOLISM
Cyanoamino acid metabolism
Formamide
3.5.1.49
Methane metabolism
Glyoxylate metabolism
Formate
CO2
4.2.1.1
HCO3-
4.2.1.104
Cyanate
Carbamate
2.7.2.2
Nitric-oxide
1.7.2.5
1.7.1.14
Nitrous-oxide
1.7.2.4
Nitrogen
1.18.6.1
1.19.6.1
Carbon fixation pathways in prokaryotes
1.7.2.1
1.7.99.4
NR
1.7.7.2
1.9.6.1
Nrt
NrtABCD
Nitrate (extracellular)
Nitrate
Nitrite (extracellular)
Nrt
Nitrite
1.7.1.4
1.7.7.1
1.7.1.15
1.7.2.2
Ammonia
6.3.4.16
Carbamoyl-P
Arginine and proline metabolism
1.4.1.2
1.4.1.3
1.4.1.4
1.4.1.13
1.4.1.14
1.4.7.1
6.3.1.2
L-Glutamine
L-Glutamate
Glutamate metabolism
1.7.1.10
1.7.99.1
1.7.2.6
Hydroxylamine
1.14.99.39
1.13.12.16
1.7.3.1
Nitroalkane
3.5.5.1
Nitrile
Oxidation state +5 +3 +2 +1 0 -1 -2 -3
Dissimilatory nitrate reduction
Nitrate
NarGHIJ
NapAB
Nitrite
NirBD
NrfAH
Ammonia
Assimilatory nitrate reduction
Nitrate
NarB
NR
NasAB
Nitrite
NIT-6
NirA
Ammonia
Denitrification
Nitrate
NarGHIJ
NapAB
Nitrite
NirK
NirS
Nitric oxide
NorBC
Nitrous oxide
NosZ
Nitrogen
Nitrogen fixation
Nitrogen
NifDKH
AnfG
Ammonia
Nitrification
Nitrate
NorAB
Nitrite
Hao
Hydroxylamine
PmoA
Ammonia
Anammox
Nitrogen
Hzo
Hydrazine
Ammonia
Nitrite
NirK
NirS
Nitric oxide
00910 1/9/14
(c) Kanehisa Laboratories

Figure S20

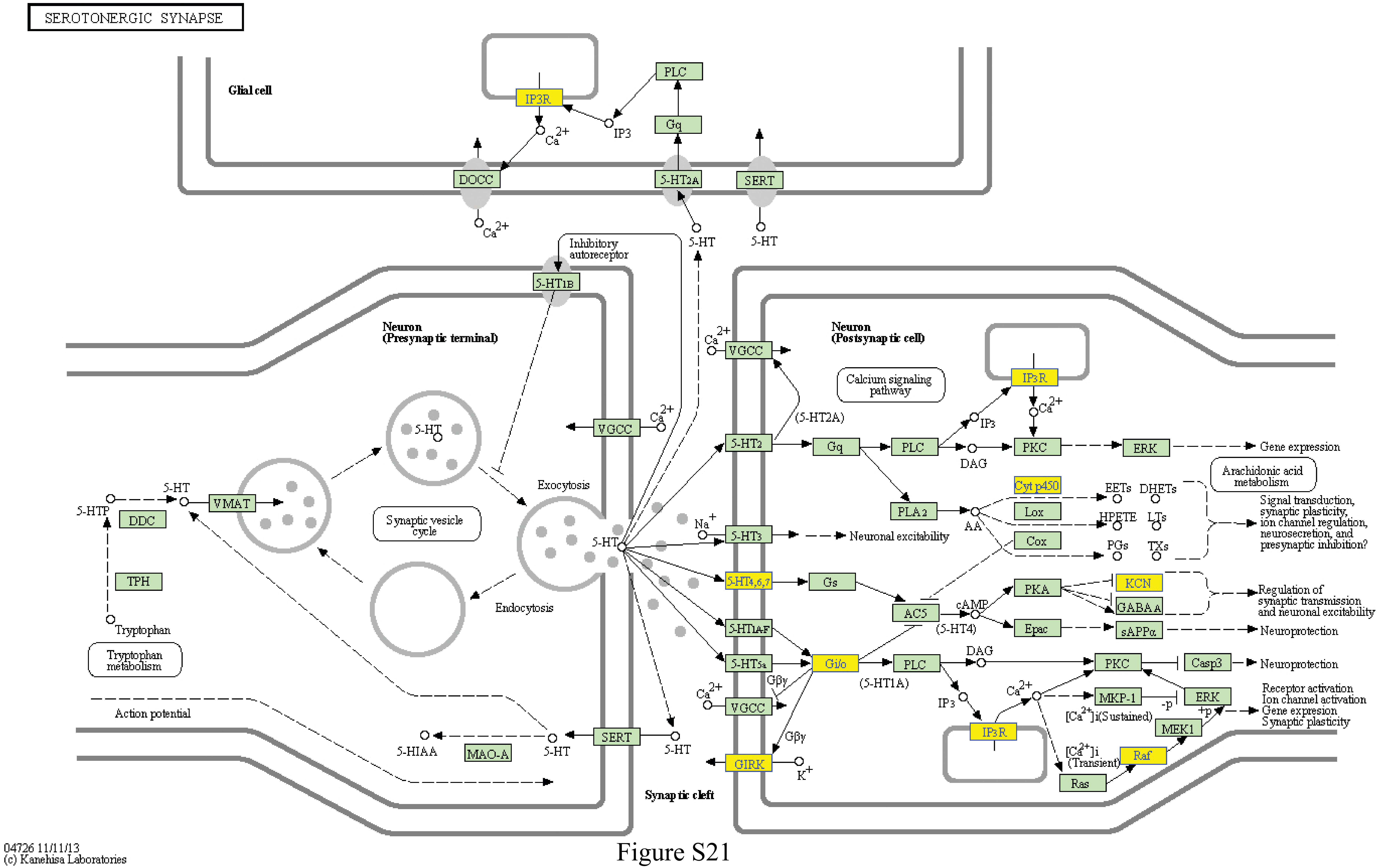

SEROTONERGIC SYNAPSE
Glial cell
IP3R
PLC
Gq
IP3
Ca2+
DOCC
5-HT2A
SERT
5-HT
Inhibitory autoreceptor
5-HT1B
Neuron (Presynaptic terminal)
Neuron (Postsynaptic cell)
VGCC
5-HT2
5-HT3
5-HT4,6,7
5-HT1A-F
5-HT5a
GIRK
Gi/o
Gs
AC5
cAMP
PKA
Epac
KCN
GABAA
sAPPα
PLA2
AA
Cyt p450
Lox
Cox
EETs
DHETs
HPETE
LTs
PGs
TXs
DAG
PKC
ERK
MKP-1
MEK1
Raf
Ras
Casp3
Calcium signaling pathway
Arachidonic acid metabolism
Gene expression
Neuronal excitability
Signal transduction, synaptic plasticity, ion channel regulation, neurosecretion, and presynaptic inhibition?
Regulation of synaptic transmission and neuronal excitability
Neuroprotection
Receptor activation Ion channel activation Gene expression Synaptic plasticity
VMAT
DDC
TPH
5-HTP
Tryptophan
Tryptophan metabolism
Synaptic vesicle cycle
Exocytosis
Endocytosis
Action potential
MAO-A
5-HIAA
Synaptic cleft
Gβγ
Na+
K+
(5-HT2A)
(5-HT4)
(5-HT1A)
[Ca2+]i(Sustained)
[Ca2+]i (Transient)
04726 11/11/13
(c) Kanehisa Laboratories

Figure S21

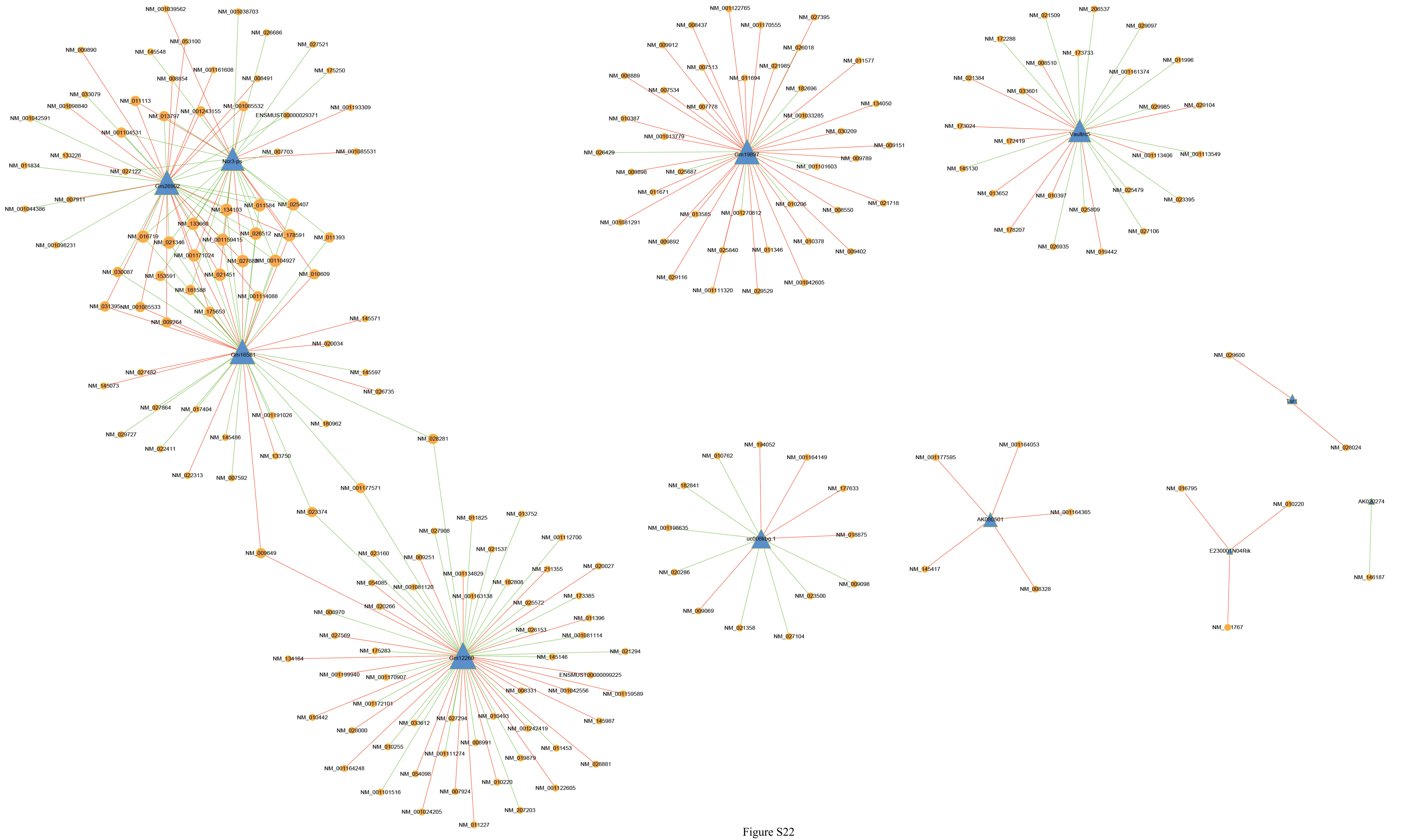
Ncr3-ps
Gm26902
Gm16581
Gm12260
Gm19897
Vaultrc5
Tsr1
uc008kbg.1
AK080501
E230001N04Rik
AK020274

Figure S22
